# SCSEQ: A web tool for analyzing single-cell RNA-seq data

**DOI:** 10.1093/gigascience/giag029

**Published:** 2026-05-05

**Authors:** Shiyu Du, Pengcheng Sun, Li Shen, Jian He

**Affiliations:** Qingdao Institute of Software, College of Computer Science and Technology, China University of Petroleum (East China), Shandong Key Laboratory of Intelligent Oil & Gas Industrial Software, Qingdao 266580, China; Qingdao Institute of Software, College of Computer Science and Technology, China University of Petroleum (East China), Shandong Key Laboratory of Intelligent Oil & Gas Industrial Software, Qingdao 266580, China; State Key Laboratory of Systems Medicine for Cancer, Center for Single-Cell Omics, School of Public Health, Shanghai Jiao Tong University School of Medicine, Shanghai 200025, China; State Key Laboratory of Systems Medicine for Cancer, Center for Single-Cell Omics, School of Public Health, Shanghai Jiao Tong University School of Medicine, Shanghai 200025, China

**Keywords:** single-cell RNA sequencing, data analysis platform, web-based tool, machine learning

## Abstract

**Background:**

Single-cell RNA sequencing has emerged as a powerful approach to reveal cellular heterogeneity within biological systems. With the continuous advancement of high-throughput sequencing technologies, studies are generating vast amounts of complex data, posing a significant challenge for researchers in effective data processing and analysis.

**Results:**

To address this issue, we developed SCSEQ, an interactive web-based bioinformatics analysis platform. This platform enables even users without programming expertise to conveniently process and analyze sequencing data. SCSEQ provides a comprehensive workflow encompassing data preprocessing, normalization, clustering, dimension reduction, differential expression analysis, cell type identification, and downstream analyses. The downstream analysis tasks include gene enrichment analysis, transcription factor analysis, cell–cell communication analysis, copy number variation detection, trajectory inference, and pan-cancer analysis. SCSEQ facilitates information transfer between different workflows, accepts various input formats, and generates graphical and tabular outputs. As a user-friendly platform, we enhance user experience through detailed parameter settings and dynamic interactions. This enables users to precisely regulate research processes and customize result figures. Additionally, we provide comprehensive user manuals to assist with parameter configuration and workflow execution.

**Conclusions:**

SCSEQ provides an intuitive and convenient solution for single-cell transcriptome sequencing data analysis. Our platform has successfully completed full-process analyses on real-world data with reliable results, demonstrating its applicability in practical scenarios. The platform is available at https://scseq.com.cn/.

## Key Points

- SCSEQ provides a no-code pipeline for single-cell transcriptome data analysis from raw data to publication-quality visualizations.
- A highly integrated system that enables flexible fine-tuning and real-time interactive visualization guarantees reliable downstream data analysis.
- Cell-type annotation is supported with models trained on user datasets.
- Cell-type annotation is improved with RAG-enhanced large language models.

## Introduction

Single-cell sequencing technology, as a major breakthrough in modern life sciences, enables high-throughput sequencing analysis of genomes, transcriptomes, and epigenomes at the individual cell level. This technology goes beyond traditional bulk sequencing by effectively uncovering cellular heterogeneity and precisely delineating gene expression profiles. It provides novel insights and tools for advancing precision medicine and personalized therapy [1]. It can reveal gene expression profiles at single-cell resolution, thereby identifying cell types, states, and intercellular interactions, and has enabled the construction of comprehensive cell atlases across tissues and developmental stages [2]. This provides powerful tools for differential gene expression analysis and alternative splicing studies at the transcriptome level, making it a hot research topic. Currently, single-cell RNA sequencing (scRNA-seq) has become a robust technique for obtaining gene expression profiles at single-cell resolution [3], offering new perspectives for uncovering cellular heterogeneity [4].

Since the pioneering work of Tang et al. [5], which first applied high-throughput sequencing to single cells, the field has rapidly expanded with the development of diverse single-cell omics techniques, such as scWGS (Single-cell Whole Genome Sequencing)[6], scBS-seq (Single-cell Bisulfite Sequencing)[7], and scGRO-seq (Single-cell Nascent RNA sequencing)[8]. However, the rapid accumulation of complex and large-scale datasets poses significant challenges for effective data analysis. In 2021, Zappia and Theis reported that the scRNA tools database had cataloged over a thousand single-cell analysis tools [9]. Among these, 2 computational ecosystems dominate the single-cell analysis landscape: Seurat [10] for R users and Scanpy [11] for Python users. However, their command-line interfaces and requirement for programming expertise pose significant barriers for many researchers lacking extensive coding experience. Moreover, these tools are confined to packages developed in their respective programming languages [12], which hinders the broader adoption of sequencing technologies. In contrast, tools with intuitive graphical user interfaces can significantly facilitate data analysis for researchers and clinicians. Recently, several studies have begun to provide interactive visualization of single-cell datasets through web applications built with frameworks such as Shiny, enabling users to explore published datasets more conveniently [13]. However, these implementations are typically designed for specific datasets and lack scalable computing resources and integrated analytical workflows. As a result, they remain limited compared with cloud-based platforms that support flexible data upload, large-scale computation, and comprehensive analysis pipelines.

To address this gap, we developed SCSEQ, an integrated and user-friendly web server. It enables comprehensive analyses of single-cell transcriptome data without requiring any programming knowledge. By offering intuitive workflows, extensive parameter customization, and detailed user guidance, SCSEQ aims to make advanced single-cell transcriptome sequencing data analyses accessible to more researchers and clinicians. This platform not only facilitates routine analytical workflows but also provides extensive, specialized downstream functions. It integrates a wide array of benchmark-validated tools—including Seurat, Harmony [14], SCENIC [15], CellChat [16], InferCNV [17], Monocle [18], and CellTypist [19]—and offers an expanded suite of downstream analyses such as differential expression, gene enrichment, transcription factor analysis, cell–cell communication, copy number variation, trajectory inference, and pan-cancer analysis. This integrated and updatable design makes SCSEQ a more thorough and versatile solution for single-cell transcriptomic studies, significantly enhancing its value in the rapidly evolving fields of single-cell biology and related disciplines.

The main advantages of SCSEQ are as follows:

1. We have integrated more methods, including benchmark-validated methods and state-of-the-art methods, and are able to continuously update and add more excellent methods for users to use, so we are equipped to tackle a broader spectrum of downstream analytical tasks.
2. We introduce an advanced cell annotation algorithm based on machine learning, which allows users to upload their own datasets, train models, and annotate cells using custom or built-in models.
3. We have explored artificial intelligence (AI) tools in single-cell transcriptomics analysis, integrating existing methods and leveraging large models. A Retrieval-Augmented Generation (RAG)–enhanced large language model can be used to assist in cell-type decision-making.
4. We have designed our platform with numerous adjustable parameters. This allows users to process and analyze their data according to their specific requirements.
5. We offer diverse visualization options. Users can choose what to display and adjust parameters. Real-time adjustments and previews are supported, and visualization results can be saved locally for research or sharing.

## Related Work

The computational analysis of scRNA-seq data is dominated by powerful programming frameworks such as Seurat and Scanpy, which provide comprehensive analytical pipelines. Recent efforts such as SeuratExtend further streamline single-cell workflows by integrating extended analytical capabilities within a unified framework [20]. In parallel, recent methodological advances in single-cell analysis, including deep learning–based representation approaches such as scGraph2Vec [21], have further improved the ability to capture complex gene relationships from high-dimensional data. However, their command-line interfaces and dependency on specific programming languages restrict their usability for nonspecialists. In response, both academic and commercial efforts have led to the development of web-based servers with graphical user interfaces [22–24]. While platforms such as ASAP [25], ICARUS [26], and CELLAR [27] have matured in handling basic analytical tasks—including data preprocessing, quality control, and cell clustering—their capabilities in advanced downstream analyses remain limited. Particularly for advanced requirements like copy number variation analysis and pan-cancer analysis, most existing platforms offer limited support. Some platforms have begun integrating specialized functions: for instance, ASAP, ICARUS, and CELLAR support cell annotation; OmicStudio [28] incorporates gene set enrichment analysis (GSEA) [29]; SciAp [30] includes trajectory inference; and ezSingleCell [31] offers cell–cell communication analysis. It is worth noting that although these platforms have made valuable attempts in multiomics data analysis, their current analytical capabilities are still insufficient to comprehensively explore single-cell transcriptomic data. There is an urgent need to develop more thorough and professional downstream analysis solutions as a supplement.

These additions are helpful, yet their current analytical capabilities remain insufficient for comprehensive exploration of single-cell transcriptomic data; most servers still leave copy number variation analysis and pan-cancer exploration outside their scope. A single portal that marries routine steps to deep, specialized modules is still missing. SCSEQ was built to close that gap. As mentioned above, we have integrated a large number of advanced tools and can keep the tools updated continuously. These tools enable comprehensive visualization functions and also enable more complete downstream analysis. In addition, our platform provides many adjustable parameters, allowing users to modify parameters to adjust the results before analysis and adjust visualization parameters to meet personal aesthetic preferences after analysis. For inexperienced users, we provide default parameters to simplify the operation. All parameters and corresponding results are systematically archived, enabling users to track and analyze the source and compare the results of different parameter settings to determine the best configuration. Our implementation and optimization of data processing and visualization will help researchers analyze sequencing data.

## Methods

**Figure 1:**
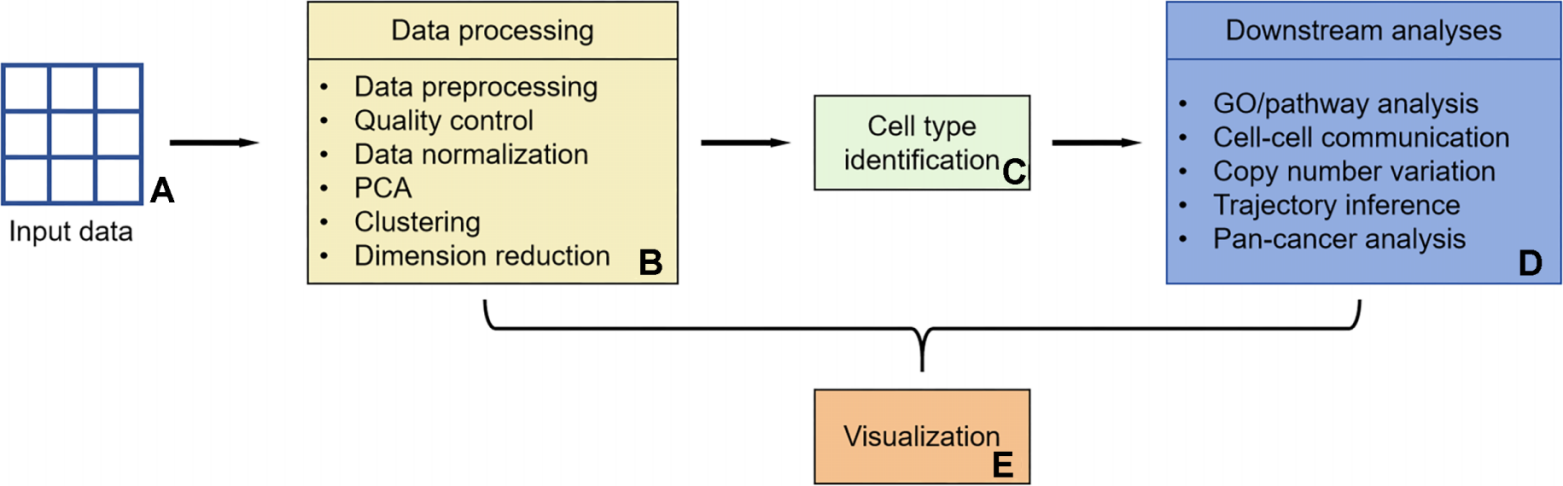
Analysis process. User-submitted data go through 3 key analytical stages: data processing, cell-type identification, and downstream analyses. Visual results from these processes are showcased on the front page.

The platform’s data analysis workflow is shown in Fig. 1. Users start projects by uploading sequencing data. The backend then runs a basic analysis (Fig. 1B) based on the uploaded data and project details. Once the basic analysis is confirmed as accurate, the system moves on to advanced analysis. Since many follow-up tasks rely on cell annotations, we place special emphasis on the cell-type identification step. SCSEQ offers multiple annotation methods, and after users confirm the annotation results, downstream analyses can proceed (Fig. 1D). Both basic and advanced analyses are controlled by user-defined parameters. All results are stored for visualization and displayed to users through the frontend interface. A tutorial for users without professional knowledge is available at [32]. Next, we will introduce the methods used in this platform.

### Bioinformatics software tools

#### Basic analysis

For the basic analysis phase, we primarily employed methods from the Seurat package. Seurat is an R package tailored for scRNA-seq data analysis. It provides a comprehensive toolkit that enables researchers to extract meaningful biological insights from raw data and reveal cellular heterogeneity.

We performed quality control in Seurat by calculating 4 key metrics for each cell: the number of detected genes (nFeature_RNA), UMI counts (nCount_RNA), the percentage of mitochondrial genes (percent.mt), and the expression proportion of hemoglobin genes (percent.hb). Following cell filtration, we normalized the data using log-normalization. Highly variable genes were identified using the FindVariableFeatures function, followed by dimensionality reduction through principal component analysis (PCA). Based on the PCA results, we constructed a K-nearest neighbor (KNN) graph and performed cell clustering using the FindNeighbors and FindClusters functions. Finally, we visualized the cell population clusters by further reducing dimensions with either t-SNE (t-distributed Stochastic Neighbor Embedding) or UMAP (Uniform Manifold Approximation and Projection) methods.

#### Cell-type identification

To identify cell types and lay a foundation for downstream analyses, we offer 2 main annotation methods: SingleR and CellTypist. SingleR is an R package tailored for cell-type annotation of scRNA-seq data. It infers cell types for unannotated single-cell data by comparing it to reference datasets. CellTypist is a Python package for annotating single-cell data, employing stochastic gradient descent to train logistic regression classifiers. Users can not only select CellTypist’s built-in reference models but also upload annotated data as training sets to develop customized reference models. Relative to built-in models, user-defined models typically exhibit improved compatibility with single-cell datasets, demonstrate superior performance in specific cell populations (e.g., rare or newly discovered cell types), and, when adequate training data are available, provide more accurate annotations.

Additionally, recent advances in AI have increasingly supported biological data interpretation and functional inference [33, 34]. A large language model (LLM) is employed as a supplementary annotation component. In the current implementation, the LLM is Qwen-Max (Alibaba Cloud), accessed via an API. To minimize data exposure, only the tissue name and the top 10 marker genes for each cluster are transmitted to the LLM, while raw expression matrices and cell-level data are not shared. All primary data processing and analysis are performed locally within the SCSEQ environment. LLM returns candidate cell-type labels along with supporting rationale. Because general-purpose LLMs may have limited bioinformatics knowledge, a retrieval-augmented generation strategy is adopted. RAG is an AI framework that integrates information retrieval and language generation [35]. By retrieving relevant information from an external knowledge base, RAG can provide corresponding reference for large models and enhance the model’s performance. Within the scRNA-seq workflow, the widely used single-cell transcriptome database (e.g., PanglaoDB [36]) is selected, which is often referred to for manual annotation. After data cleaning to ensure that each record contains the tissue name, marker genes, and cell-type label, the curated corpus serves as the external knowledge base. For each cluster pending annotation, relevant records are retrieved and ranked by semantic similarity computed in an embedding space. Specifically, text records are encoded using a SentenceTransformer model (text2vec-base-chinese), and the embeddings are stored and indexed in ChromaDB for vector-based similarity retrieval. The top candidate records are further reranked using a CrossEncoder model (mmarco-mMiniLMv2-L12-H384) and then supplied to the LLM as contextual references to support cell-type inference.

Nevertheless, the accuracy of this approach cannot be guaranteed, and the results should be regarded as advisory, with final decisions left to the user. The reliability of LLM predictions depends largely on the quality of clustering and the representativeness of the marker genes. When clusters are well defined and marker genes accurately reflect a single-cell population, prediction reliability is improved; however, the outputs are intended only as a supplementary reference rather than definitive annotation results.

#### Downstream analyses

Before diving into other analytical tasks, we routinely carry out differential gene expression analysis using the “FindAllMarkers” and “FindMarkers” functions from the Seurat package. These functions systematically pinpoint genes that show statistically significant expression differences between specific cell populations or conditions. Following this, we delve into Gene Ontology (GO) Enrichment Analysis. This powerful bioinformatics approach aids researchers in understanding the roles of genes or gene sets across biological processes, molecular functions, and cellular components.

To investigate transcriptional regulatory mechanisms, we incorporate SCENIC for transcription factor analysis. SCENIC enables the identification of regulons and the estimation of their activity at single-cell resolution. By integrating regulon activity scores into the analytical workflow, we systematically assess transcription factor activation patterns across different cell populations, providing insights into cell-type–specific regulatory programs.

To explore intercellular communication mechanisms, we leverage the CellChatDB reference database. It offers a comprehensive repository of ligand–receptor interactions and signaling pathways. This resource enables us to systematically analyze and visualize cell–cell communication networks within the biological system under investigation.

To detect genomic abnormalities, we conduct copy number variation (CNV) analysis to identify changes in DNA segment copy numbers. In SCSEQ, we use InferCNV, a software package that effectively distinguishes tumor cells from normal cells based on CNV profiles.

Our analytical pipeline also includes 2 advanced methods: trajectory inference and pan-cancer analysis. For trajectory inference, we use the Monocle package to reconstruct cellular developmental pathways and transitions. For pan-cancer analysis, we utilize The Cancer Genome Atlas (TCGA) data to conduct cross-cancer comparative studies at the gene level. Rather than projecting single-cell clusters onto bulk data, this module enables downstream validation of user-identified genes through expression comparison, immune correlation, and survival analyses across multiple cancer types.

### Application development technologies

#### Front end

The SCSEQ front end is built with Vue, a progressive JavaScript framework. Vue excels in responsive data binding, allowing the page to reflect data changes instantly. This real-time interactivity is ideal for visualizing analytical results and providing immediate feedback. Vue’s component-based approach lets us quickly build efficient, visually appealing web applications. This boosts development speed and enhances the user experience.

#### Back end

The backend of SCSEQ is built with Flask, a lightweight Python web framework. Flask is simple, flexible, and highly extensible. Its streamlined design makes it easy to integrate tools for diverse scenarios and manage complex tasks, which is ideal for scRNA-seq analysis. Flask supports URL parameter parsing and static file serving, ensuring robust request handling and resource access to streamline real-time data exchange.

#### Database

For database management, we selected MySQL, an open-source relational database management system recognized for its high performance, reliability, and user-friendly features. Widely adopted across applications of varying scales, MySQL serves as the backbone for systematically storing information generated by SCSEQ through 3 dedicated tables: User Table, Project Table, and Task Table.

## Implementations

In this section, we introduce the overall architecture of SCSEQ and delve into the architecture and workflow. We offer a thorough explanation of each component’s functionality and a comprehensive overview of the entire workflow, while also showcasing the system’s user-friendly features.

Building a system that is both efficient and user-friendly is of utmost importance in our research. SCSEQ opts for a front-end–back-end separation architecture. This design pattern is widely used in web application development. It separates the front-end user interface from the back-end service logic, allowing for independent deployment and maintenance. The platform design follows principles of reproducibility and standardized data management consistent with recent guidelines for computational biological systems [37]. Additionally, the use of interfaces for communication between the front end and back end simplifies functional expansion and service upgrades, thereby considerably improving the system’s scalability.

**Figure 2:**
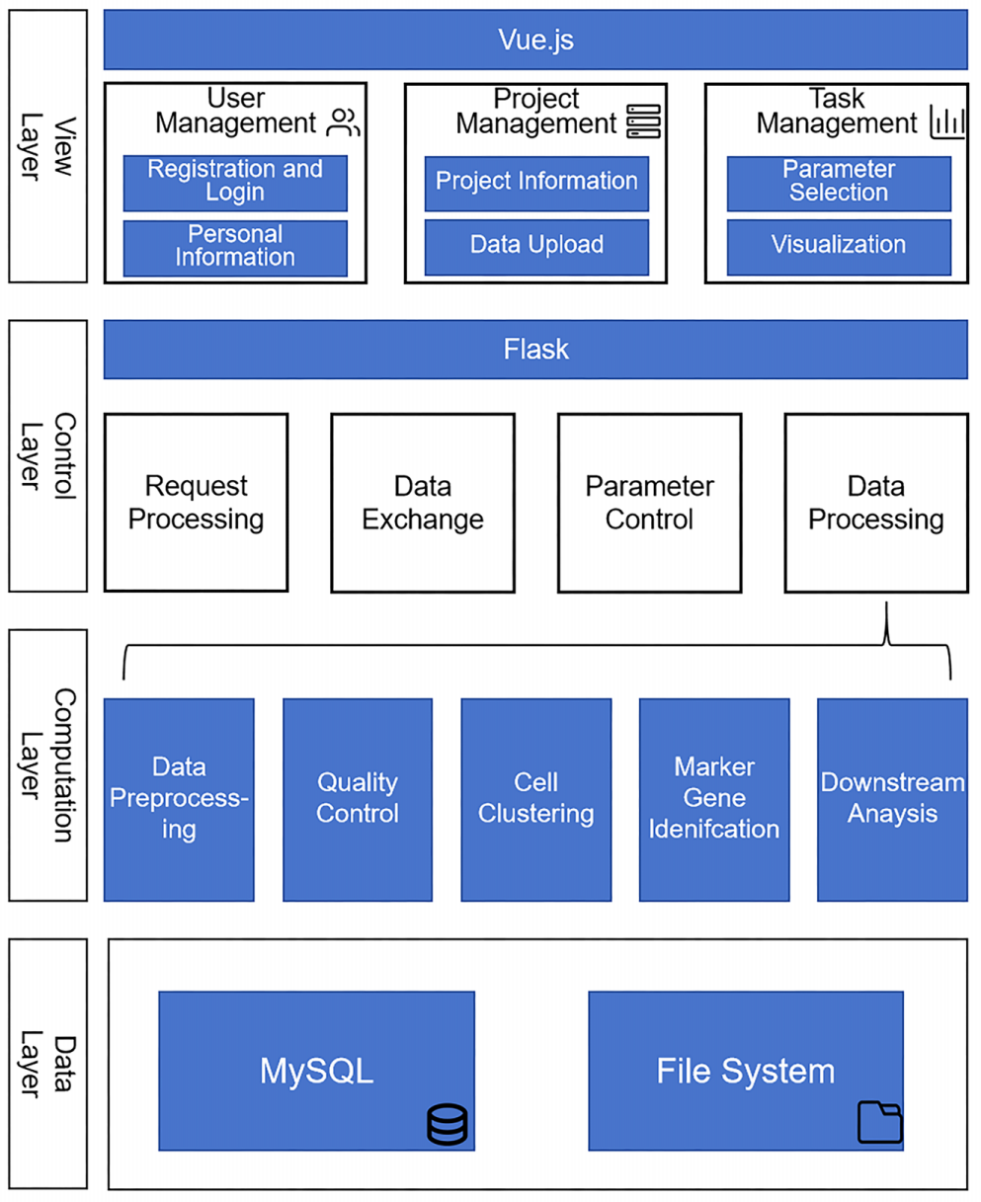
System overview. SCSEQ is composed of 4 main components. The View Layer handles user interactions. The Control Layer receives data and parameters from the View Layer and passes them to the Computation Layer to execute algorithms or functions. The Data Layer stores all relevant data and task records.

Figure 2 provides an overview of our application. SCSEQ primarily consists of 4 key components: the View Layer, Control Layer, Computation Layer, and Data Layer. The View Layer, implemented as a responsive web interface, serves as the primary interaction portal where researchers can visualize analytical results and configure parameters through intuitive graphical components. The User Management module provides centralized administration of authentication details and account preferences, while the Project Management module shows all projects under the user account. Our platform architecture supports concurrent multiproject workflows; however, each project is assigned an independent workspace and maintains strict data isolation at both the user and project levels. All project resources are logically scoped using user identifiers and project identifiers, ensuring that data access is restricted exclusively within the corresponding project context. Initial data ingestion is limited to the project initialization phase to support computational reproducibility and version control integrity. Within the Project Management interface, users can comprehensively administer existing projects while also initiating new analytical workflows by submitting requisite data files through our standardized upload protocol. After selecting their desired project, users can configure relevant parameters according to their needs and submit analysis tasks. Upon task completion, the platform will present visualization results on the interface. Users can either view these results online or download them for local storage. All chart results displayed on this platform are available for direct download and saving.

The Control Layer handles interactions between the front end and back end, as well as some back-end functions in the web server. Its main role is to process requests and data, enabling communication and data exchange between components. This layer receives files and parameter settings from the client interface, directs the Computation Layer to perform customized data processing that meets each user’s specific needs, and returns the results to the client interface. For users without programming experience, the Control Layer acts as a capable assistant. It stores uploaded data locally and passes user-defined parameters to the corresponding functions in the Computation Layer for processing and visualization. Researchers can simply interact with intuitive form fields and selection menus on the front-end interface without needing to understand the underlying technical details. After submitting tasks, users can easily monitor progress and receive visualization outputs. All analysis jobs are executed as asynchronous background tasks on the server. This design prevents the web interface from blocking during long-running computations and allows multiple tasks from different users or projects to be processed concurrently and independently. This streamlined workflow significantly reduces the technical barriers to biological data analysis. The Computation Layer contains all data-processing methods and downstream analysis algorithms. This component integrates several high-quality solutions, which will be detailed in the Methods section.

The Data Layer manages data storage and is split into database and file system parts. For the database, we use MySQL and set up 3 tables:

- User Table: This stores personal user details, including login credentials.
- Project Table: This holds all project data and links to the User Table via user_id. Besides basic info like project_id, user_id, project_name, and creation_time, it also keeps key analysis data such as species studied, user notes, raw data paths, and work directories. Each project entry is uniquely associated with a single user through user_id, and all database queries are constrained by both user_id and project_id to enforce strict separation between users and projects. This setup allows users to view and manage only their own projects within the Project Management interface.
- Task Table: This records all tasks linked to projects via project_id. It includes details like task_id, task_type, parameters, result paths, submission time, and jobid for tracking. In SCSEQ, this table does 3 main things: lets users review all past tasks, logs parameters for each task, and notes where task results are stored. All task records are scoped to their corresponding projects, preventing cross-project access. In addition, submitted tasks are scheduled and executed independently at the project level, ensuring that multiple analysis jobs can run concurrently without interfering with each other. These features make analyses traceable and reproducible, boosting SCSEQ’s usefulness.

The file system stores various files generated during platform operations. Strategies vary by file type:

- Raw Files: These are user-uploaded sequencing data, often in matrix format and storage-heavy. They are mainly used for preprocessing, after which the data are stored as RDS files. Raw files are stored within project-specific directories and are not shared across projects or users. The system has been tested on internal datasets containing more than 80,000 cells; although larger datasets require longer computation time, the analysis workflow remains stable without server timeouts or task failures.
- Intermediate Files: Generated during analysis (e.g., InferCNV creates intermediate files at each step). As they are regenerated with each task, we do not specially retain them.
- Result Files: These include charts and visualization data, which take up little space. We keep all result files, naming them with the task type and timestamp for easy comparison. Users can delete task records via the front end, which also deletes corresponding result files. Deleting a project triggers the removal of all associated files under the corresponding project directory.

## Results and Discussion

### Benchmarking SCSEQ against existing platforms

SCSEQ specializes in single-cell transcriptomics analysis, completing a complete data analysis pipeline. Throughout the analytical process, we have integrated multiple excellent methods and provided numerous analysis tools. Using these methods, users can perform basic data processing as well as advanced downstream analyses. Inspired by ezSingleCell, the integrated tools and their comparisons with other similar platforms are shown in Table 1. To provide a fair comparison, we clarify that SCSEQ focuses specifically on transcriptomic analyses and does not currently include modules for spatial transcriptomics or other multiomics analyses. In comparison, SCSEQ offers a broader range of advanced downstream analytical functionalities. For these tools, we provide default parameters while also supporting user-defined parameter inputs, ultimately obtaining high-quality visualization results.

**Table 1:** A comparative analysis of SCSEQ and current academic web platforms for single-cell analysis tasks.

Web server	Ours	ezSingle-Cell	ICARUS	ASAP	alona	Cellar	SCiAp	NASQAR	SCTK	Asc-Seurat
Clustering and dimension reduction	$\checkmark$	$\checkmark$	$\checkmark$	$\checkmark$	$\checkmark$	$\checkmark$	$\checkmark$	$\checkmark$	$\checkmark$	$\checkmark$
Cell-type identification	$\checkmark$	$\checkmark$	$\checkmark$	$\checkmark$	$\times$	$\checkmark$	$\checkmark$	$\times$	$\checkmark$	$\times$
GO/pathway analysis	$\checkmark$	$\checkmark$	$\checkmark$	$\checkmark$	$\times$	$\checkmark$	$\checkmark$	$\checkmark$	$\checkmark$	$\checkmark$
Cell–cell communication	$\checkmark$	$\checkmark$	$\times$	$\times$	$\times$	$\times$	$\times$	$\times$	$\times$	$\times$
Copy number variation	$\checkmark$	$\times$	$\times$	$\times$	$\times$	$\times$	$\times$	$\times$	$\times$	$\times$
Trajectory inference	$\checkmark$	$\times$	$\times$	$\times$	$\times$	$\times$	$\times$	$\times$	$\times$	$\times$
Pan-cancer analysis	$\checkmark$	$\times$	$\times$	$\times$	$\times$	$\times$	$\times$	$\times$	$\times$	$\times$

Note: $\checkmark$ and $\times$ denote whether the web server supports the functionality.

### Advantages of SCSEQ

Benefiting from the reasonable system architecture described previously, SCSEQ has numerous user-friendly and practical features:

#### Multitask concurrency and flexible task scheduling

**Figure 3:**
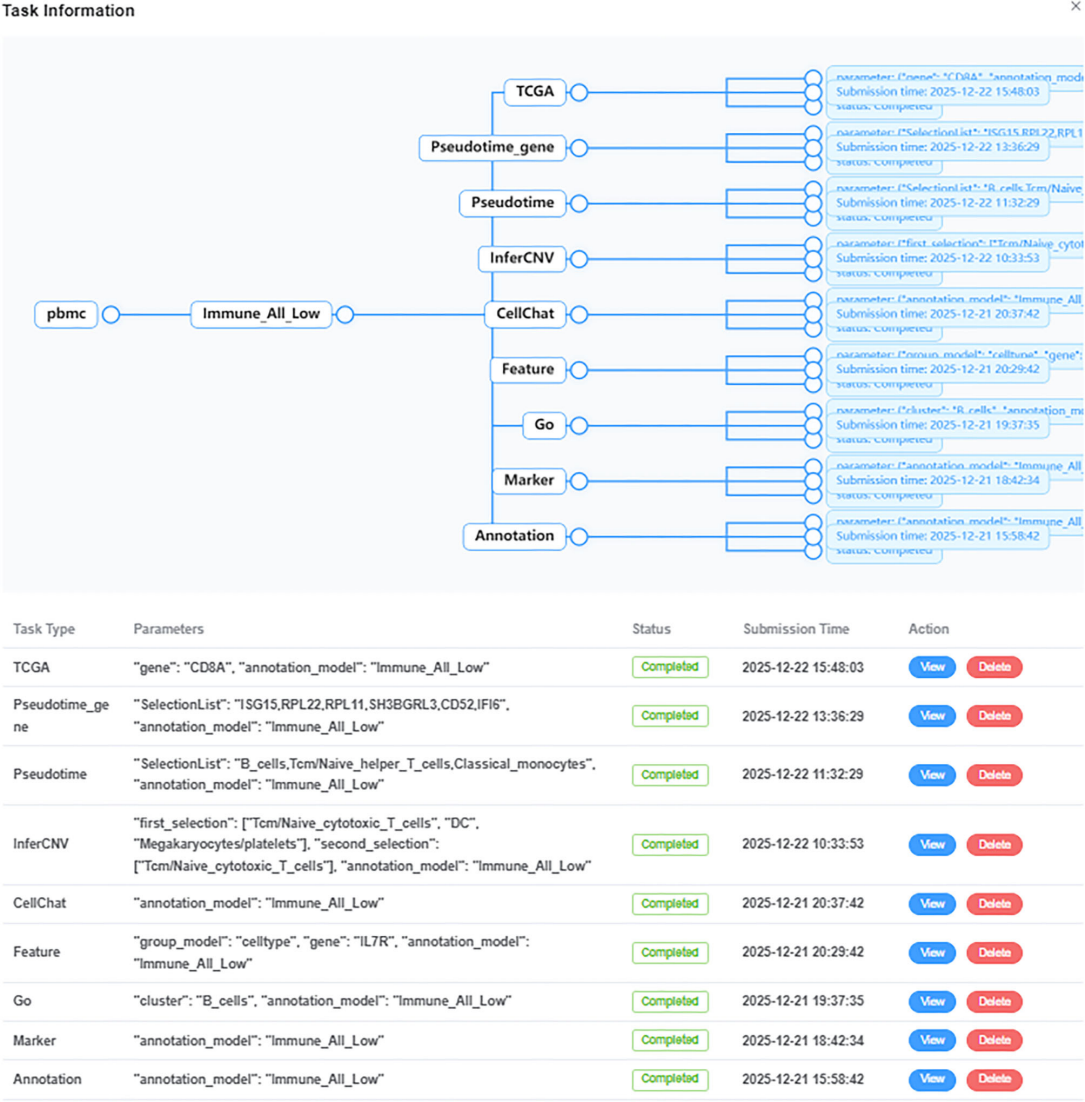
Task Information Dialog. This page displays task information under the current project and builds a tree view based on the annotation method.

The system extends task management functionality on the project page (Fig. 3),implemented as a dialog interface. This allows users to view comprehensive task information, including task type, relevant parameters, execution status, and submission time—all queried from the Task Table in the database. The operation panel allows users to review results or delete records for any task. This design improves task scheduling. Users can submit tasks, shut down their computers temporarily, and check results later. It also helps in planning follow-up analysis workflows. Additionally, the system supports multitask concurrency. Users can run multiple projects at the same time without waiting for current tasks to finish. They can monitor all task statuses and access results through the unified task management dialog.

#### Comprehensive parameter configuration

SCSEQ incorporates a wide range of adjustable parameters, enabling users to precisely control the analytical process. Thanks to the well-designed task table, SCSEQ can save the parameters configured by users for each submitted task. This allows for retrospective analysis and comparative evaluation of results obtained with different parameter settings. Additionally, the system provides default parameter sets optimized for most analytical workflows, simplifying user operations. For visualization outputs, parameter controls are implemented, allowing users to customize elements such as font sizes and axis ranges, thus achieving personalized data visualization.

#### Interactive visualization and diverse visualization outputs

To enhance user experience and deliver richer insights, SCSEQ uses interactive visualizations with ECharts components. For example, in marker gene dotplots, users can hover over nodes to see detailed information like “Cell Type,” “Gene Type,” “avg exp,” and “pct1” values. SCSEQ also offers various visualization types, including violin plots, scatterplots, bar charts, dotplots, circle plots, heatmaps, box plots, and forest plots. These options allow users to select the most intuitive representation for their analytical tasks.

#### Real-time updates and immediate feedback

The platform’s cloud tools allow real-time updates to existing results. For example, users can choose to display the number of genes per cell population in the marker gene results, and the chart will update immediately. For cloud analyses requiring computation to produce results, users need to wait until the analysis task completes to view the visualization outcomes. Once a task is complete, the results are instantly visible on the current page. This design minimizes debugging time for users, aids in understanding how parameters affect outcomes, helps identify more suitable parameters, and ultimately leads to better results.

### Data analysis

**Figure 4:**
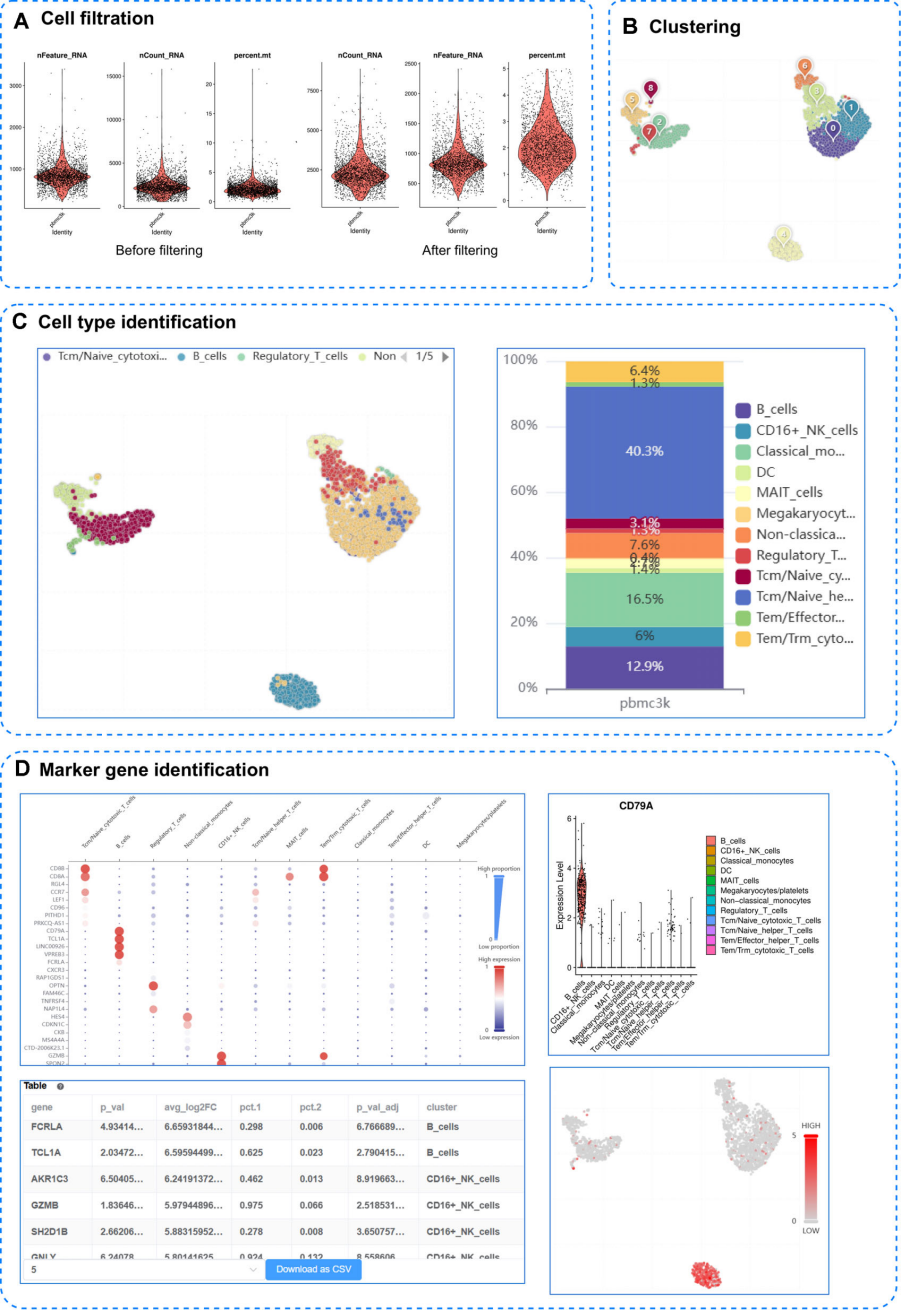
Basic analysis and cell annotation. (A) Comparison before and after cell filtration. (B) Visualization of clustering results. (C) CellTypist annotation results and cell proportion plots. (D) Marker gene tables and dotplot visualizations. Distribution of individual genes across all cell populations.

**Figure 5:**
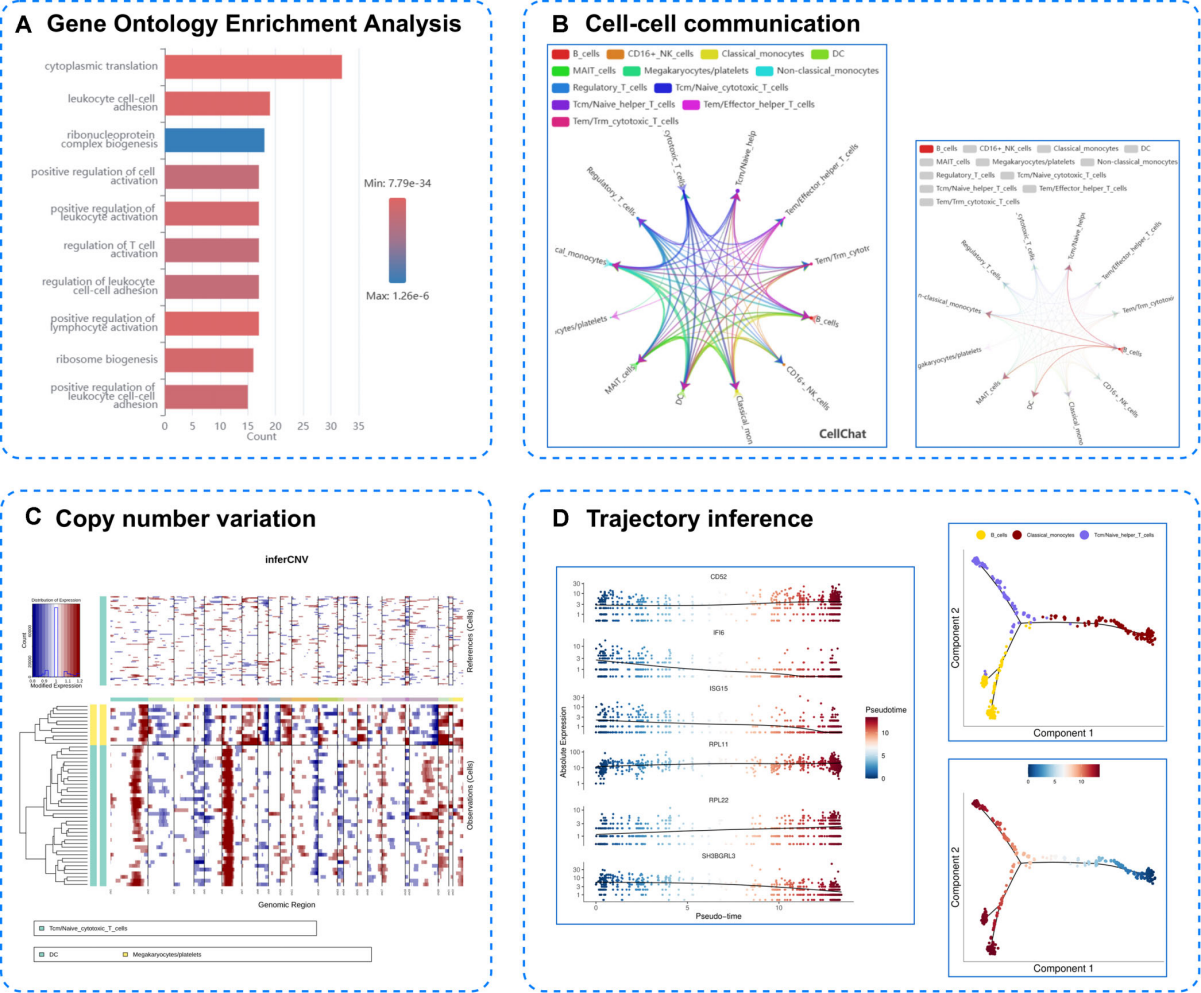
Advanced analysis. (A) GO enrichment results of B cells. (B) Cell–cell communication analysis using CellChatDB. (C) Copy number variation analysis using InferCNV. (D) Cell and gene expression dynamics along trajectories.

**Figure 6:**
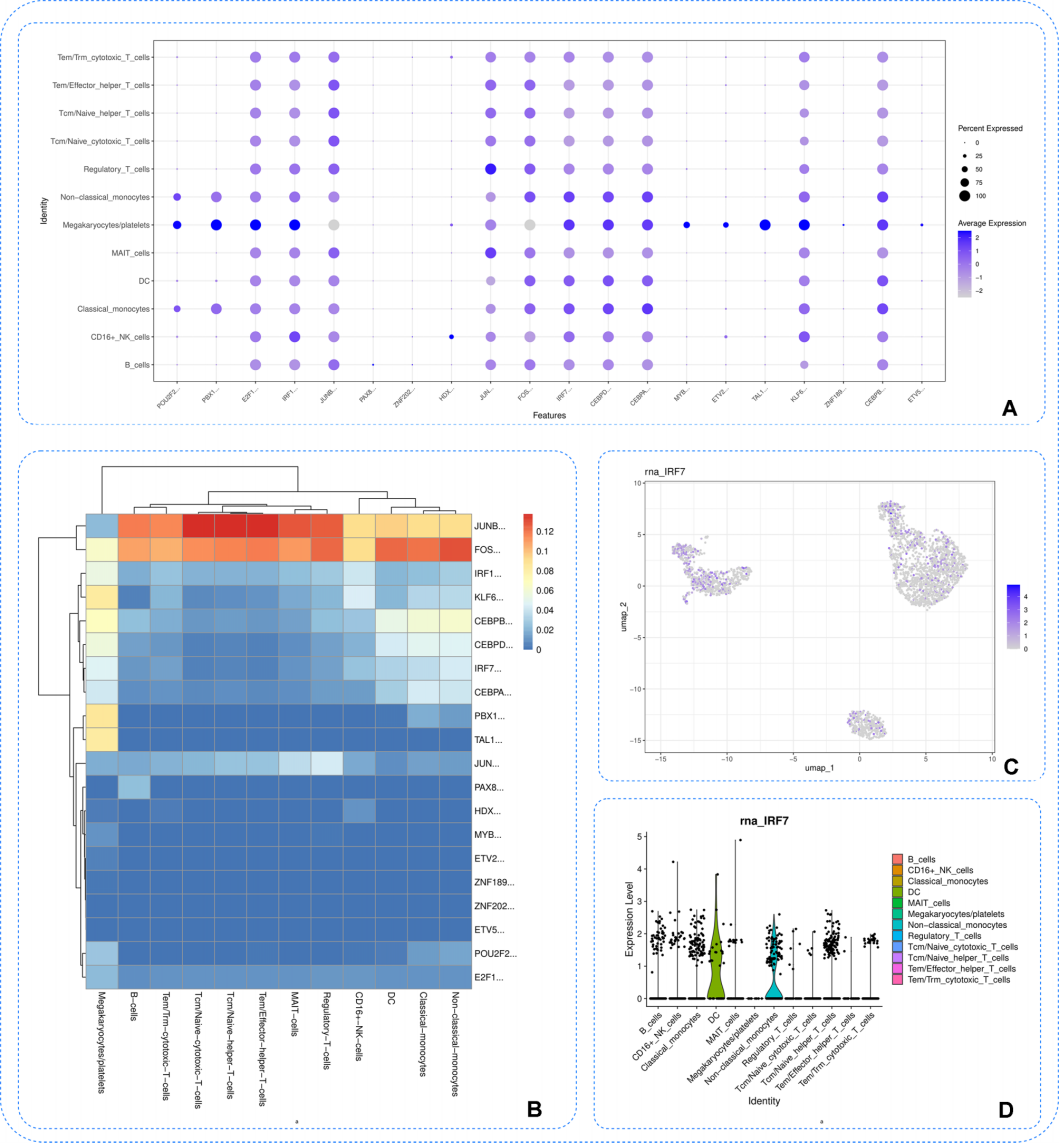
Transcription factor analysis. (A) Dot plot of regulon activity across annotated cell types. (B) Heatmap showing average regulon activity among cell populations. (C) UMAP visualization of IRF7 regulon activity. (D) Violin plot of IRF7 regulon activity across different cell types.

**Figure 7:**
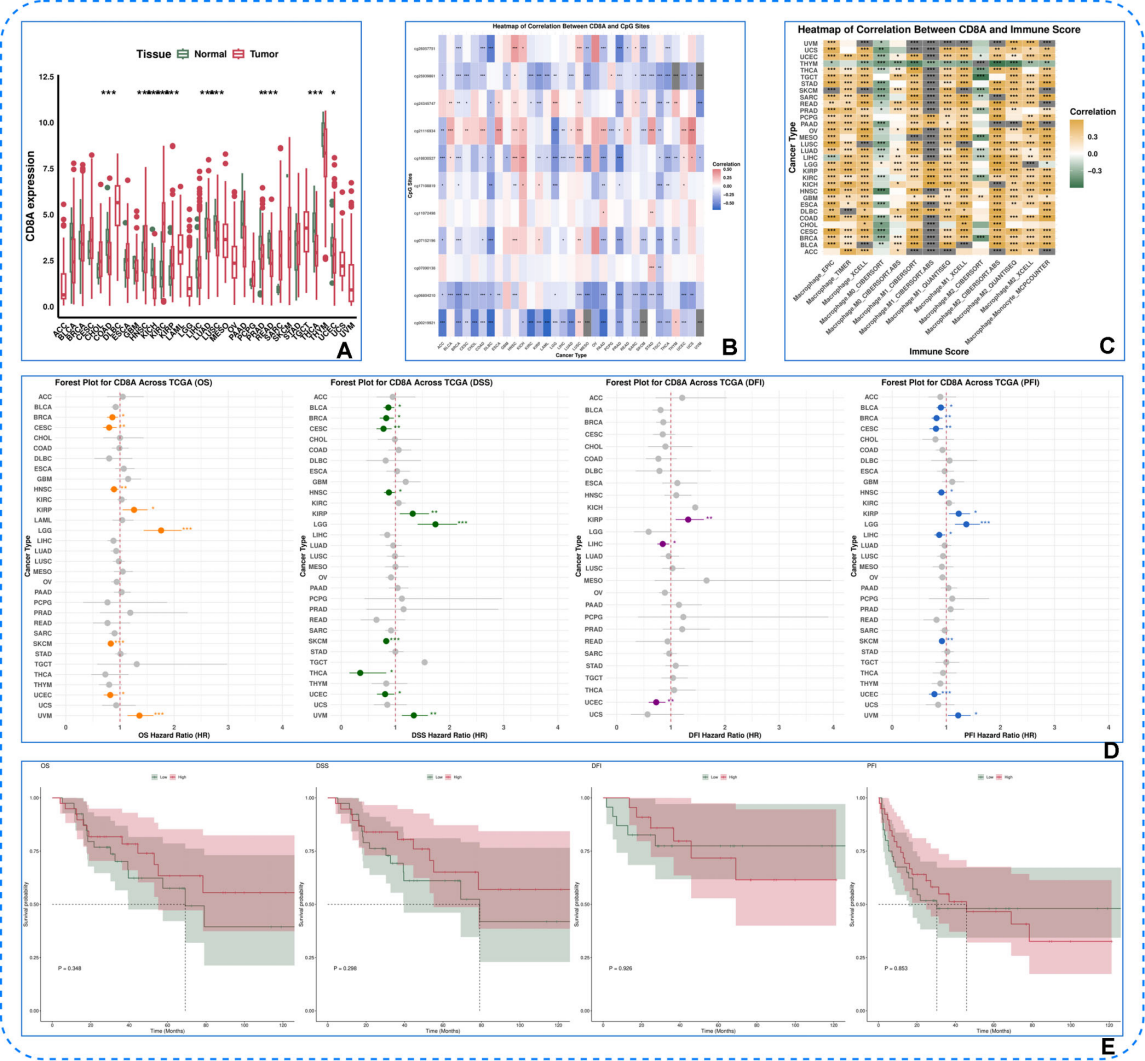
Transcription factor analysis. (A) Pan-cancer boxplot. (B) CpG methylation heatmap. (C) Gene–Immune Infiltration Correlation Heatmap. (D) Pan-cancer univariate Cox regression forest plot. (E) Pan-cancer Kaplan–Meier survival analysis.

To showcase SCSEQ’s capabilities, we analyzed a dataset of 2,700 peripheral blood mononuclear cells (PBMCs) [38] and present the results (Figs. 4, 5, 6, and 7).

After uploading the data, we conducted basic analysis using default parameters from Seurat’s official documentation. SCSEQ initially displayed the overall data distribution and provided quality control metrics such as nFeature_RNA, nCount_RNA, and percent.mt (percent.hb was optional and not used in this case). We used a violin chart to visualize the data before and after cell filtering (Fig. 4A). The subsequent steps involved log-normalization, identification of highly variable genes, data scaling, and PCA. Based on the PCA results, we constructed a KNN graph and performed clustering at a resolution of 0.5 (default), resulting in 9 distinct clusters (Fig. 4B).

In the process of cell annotation, we used celltype’s “Immune_All_Low” reference set. This identified 12 cell populations, including B cells, CD16^+^ natural killer cells, classical monocytes, DCs (Dendritic Cells), MAIT (Mucosal-Associated Invariant T cells) , megakaryocytes/platelets, nonclassical monocytes, regulatory T cells, Tcm/naive cytotoxic T cells, Tcm/naive helper T cells, Tem/effector helper T cells, and Tem/Trm cytotoxic T cells. We also show the proportion of each cell type in the total cell population (Fig. 4C). To aid downstream analysis, we calculated marker genes for each annotated cluster and present them in tables and dot plots (Fig. 4D). Additionally, users can select specific genes to examine their expression patterns across all cell populations. In this study, CD79A was selected as an example, and the same gene was used to generate both the feature plot and the violin plot shown in Fig. 4D.

After basic analysis, advanced analysis can be performed. We first performed GO Enrichment Analysis. We selected biological processes related to B cells and visualized the top 10 terms by “Count” value using bar plots (Fig. 5A).

For cell–cell communication analysis, we employed the CellChatDB database to examine ligand–receptor pairs (Fig. 5B). As shown in the left panel of Fig. 5B, all cell types are selectable, so users can also selectively examine interaction results between specific cell types of interest and other cells. For instance, in the right panel of Fig. 5B, we selected B cells for visualization.

Copy number variation analysis was conducted using InferCNV, with Tcm/Naive_cytotoxic_T_cells as the reference set alongside DC and megakaryocyte/platelet populations (Fig. 5C). For trajectory inference analysis, we selected the 3 most abundant cell populations: B_cells, Tcm/Naive_helper_T_cells, and classical_monocytes. The platform also supports examining gene expression dynamics along trajectories (Fig. 5D). We specifically analyzed the temporal expression patterns of ISG15, RPL22, RPL11, SH3BGRL3, CD52, and IFI6 genes.

We next performed transcription factor analysis to investigate transcription factor regulatory activity. Regulon activity scores were calculated and integrated for downstream analysis. A dot plot summarizes regulon activity across annotated cell types (Fig. 6A), and a heatmap presents average activity patterns among cell populations (Fig. 6B). We then focused on the IRF7 regulon, visualizing its spatial distribution on the UMAP embedding (Fig. 6C). A violin plot further illustrates the distribution of IRF7 regulon activity across different cell types (Fig. 6D).

Pan-cancer analysis of the CD8A gene was performed using TCGA tools, with results presented through boxplots, heatmaps, and forest plots (Fig. 7). The boxplot (Fig. 7A) shows CD8A expression differences between tumor (red) and normal tissues (green) across multiple cancer types. Two heatmaps highlight (i) links between CD8A expression and CpG methylation (Fig. 7B), indicating epigenetic regulation of T-cell infiltration and treatment potential, and (ii) relationships between CD8A expression and macrophage immune scores across cancer types (Fig. 7C), shedding light on immune cell interactions and tumor diversity. A forest plot (Fig. 7D) presents survival analysis results for target gene expression data across different cancer types in TCGA. To further evaluate the prognostic value of CD8A, Kaplan–Meier survival analysis was performed in adrenocortical carcinoma (ACC), with patients stratified into high- and low-expression groups based on the median CD8A level (Fig. 7E). The Kaplan–Meier curves illustrate differences in overall survival between the 2 groups, and statistical significance was assessed using the log-rank test, providing direct evidence of the clinical relevance of CD8A expression in ACC.

## Conclusion

SCSEQ consolidates an unusually broad slice of single-cell science into one coherent web workspace. Cell-type labeling, gene enrichment analysis, transcription factor analysis, cell–cell communication analysis, copy number variation detection, trajectory inference, and pan-cancer analysis and high-resolution visualization are no longer scattered across different packages; they are accessible through a single menu, callable with a few clicks and parameter sliders. Behind the interface sits a containerized back end that wraps rigorously benchmarked tools—Seurat, Harmony, CellChat, SCENIC, InferCNV, Monocle, and CellTypist—so that every step inherits the statistical robustness of the original code while gaining the reproducibility of version-tracked workflows. Users can therefore move from raw count matrix to publication-grade figures without installing interpreters, managing dependencies, or editing scripts. The platform’s parameter logging and real-time visualization further transform exploratory analysis into an intuitive feedback loop: adjust parameters, observe results, and export satisfactory images.

Looking forward, the platform’s impact on bioinformatics will continue to grow, and we are extending the architecture along 2 complementary axes. First, our platform currently focuses solely on single-cell RNA sequencing and does not support spatial transcriptomics or single-cell multiomics (scMultiomics), representing one of our key directions for future development. In addition, the AI approaches applied in our cell-type identification module have demonstrated excellent performance, which inspires us to incorporate more AI analysis tools in future updates to further enhance the performance of SCSEQ and contribute to the rapid development of single-cell biology and related disciplines.

## Supplementary Material

giag029_Authors_Response_To_Reviewer_Comments_original_submission

giag029_GIGA-D-25-00531_original_submission

giag029_GIGA-D-25-00531_revision_1

giag029_Reviewer_1_Report_original_submission Reviewer 1 -- 1/18/2026

giag029_Reviewer_1_Report_revision_1 Reviewer 1 -- 3/6/2026

giag029_Reviewer_2_Report_original_submission Reviewer 2 -- 1/21/2026

giag029_Reviewer_2_Report_revision_1 Reviewer 2 -- 3/2/2026

## Data Availability

The dataset used to illustrate SCSEQ’s performance consists of 2,700 peripheral blood mononuclear cells sequenced on the Illumina NextSeq 500. Raw data and the processed count matrix are available from the 10x Genomics [38].

## Availability of Source Code and Requirements

- Project name: SCSEQ
- Project homepage:  https://github.com/knight-spc/SCSEQ
- Operating system(s): Platform independent
- Programming language: Python, Vue
- Other requirements: Anaconda (Recommended)
- License: GPL-3.0 license

## Abbreviations

ACC, adrenocortical carcinoma; AI, artificial intelligence; CNV: copy number variation; GO: Gene Ontology; GSEA: gene set enrichment analysis; KNN: K-nearest neighbor; LLM: large language model; PBMC: peripheral blood mononuclear cell; PCA: principal component analysis; RAG: Retrieval-Augmented Generation; scRNA-seq: single-cell RNA sequencing; TCGA: The Cancer Genome Atlas.
