# Supplementary material for: SCSEQ: A web tool for analyzing single-cell RNA-seq data
Source: Gigascience. 2026 May 5;15:giag029. doi: 10.1093/gigascience/giag029 (PMC13142160; doi:10.1093/gigascience/giag029)
Supplement: giag029_GIGA-D-25-00531_original_submission [file giag029_giga-d-25-00531_original_submission.pdf]

GigaScience

SCSEQ: A web tool for analyzing single-cell RNA-seq data

--Manuscript Draft--

**Manuscript Number:**	GIGA-D-25-00531
**Full Title:**	SCSEQ: A web tool for analyzing single-cell RNA-seq data
**Article Type:**	Research
**Funding Information:**	National Key Research and Development Program of China (2022YFD2101503) — Prof. Jian He
**Abstract:**	Single-cell RNA sequencing has emerged as a powerful approach to reveal cellular heterogeneity within biological systems. With the continuous advancement of high-throughput sequencing technologies, studies are generating vast amounts of complex data, posing significant challenge for researchers in effective data processing and analysis. To address this issue, we developed SCSEQ, an interactive web-based bioinformatics analysis platform. This platform enables even users without programming expertise to conveniently process and analyze sequencing data. SCSEQ provides a comprehensive workflow encompassing: data preprocessing, normalization, clustering, dimension reduction, differential expression analysis, cell type identification and downstream analyses. The downstream analysis tasks include gene enrichment analysis, cell-cell communication analysis, copy number variation detection, trajectory inference, and pan-cancer analysis. SCSEQ facilitates information transfer between different workflows, accepts various input formats, and generates graphical and tabular outputs. As a user-friendly platform, we enhance user experience through detailed parameter settings and dynamic interactions. This enables users to precisely regulate research processes and customize result figures. Additionally, we provide comprehensive user manuals to assist with parameter configuration and workflow execution. SCSEQ provides an intuitive and convenient solution for single-cell transcriptome sequencing data analysis. Our platform has successfully completed fullprocess analyses on real-world data with reliable results, demonstrating its applicability in practical scenarios.
**Corresponding Author:**	Jian He<br>Shanghai Jiao Tong University School of Medicine<br>Shanghai, CHINA
**Corresponding Author Secondary Information:**	
**Corresponding Author's Institution:**	Shanghai Jiao Tong University School of Medicine
**Corresponding Author's Secondary Institution:**	
**First Author:**	Shiyu Du
**First Author Secondary Information:**	
**Order of Authors:**	Shiyu Du
	Pengcheng Sun
	Li Shen
	Jian He
**Order of Authors Secondary Information:**	
**Additional Information:**	
**Question**	**Response**
Are you submitting this manuscript to a special series or article collection?	No

Powered by Editorial Manager® and ProduXion Manager® from Aries Systems Corporation

**Experimental design and statistics**<br><br>Full details of the experimental design and statistical methods used should be given in the Methods section, as detailed in our Minimum Standards Reporting Checklist. Information essential to interpreting the data presented should be made available in the figure legends.<br><br>Have you included all the information requested in your manuscript?	Yes
**Resources**<br><br>A description of all resources used, including antibodies, cell lines, animals and software tools, with enough information to allow them to be uniquely identified, should be included in the Methods section. Authors are strongly encouraged to cite Research Resource Identifiers (RRIDs) for antibodies, model organisms and tools, where possible.<br><br>Have you included the information requested as detailed in our Minimum Standards Reporting Checklist?	Yes
**Availability of data and materials**<br><br>All datasets and code on which the conclusions of the paper rely must be either included in your submission or deposited in publicly available repositories (where available and ethically appropriate), referencing such data using a unique identifier in the references and in the “Availability of Data and Materials” section of your manuscript.<br><br>Have you have met the above requirement as detailed in our Minimum Standards Reporting Checklist?	Yes

Powered by Editorial Manager® and ProduXion Manager® from Aries Systems Corporation

GigaScience has policies and guidelines in place for the use of generative AI-writing tools such as ChatGPT. If you have used such writing tools to assist with writing the manuscript this must be declared and cited in the text. Authors should not list AI-writing tools and other AI-assisted technologies as an author or co-author and should acknowledge that they are fully responsible for text generated or refined by AI-writing tools.<p><br><br>A summary of use (particularly in the introduction or among methods) needs to be included at the end of the paper, and the outputs should also be included as a supplementary file hosted in GigaDB or other open repositories. Please read our guidelines for more information. <p><br><br>By submitting to GigaScience, you are aware of the journal's AI-writing tools policy, and if you have declared use of such tools below, you have acknowledged this where appropriate in your manuscript and have made a summary of use and outputs available. <p><br><b>AI-assisted writing tools have been used in the preparation of this manuscript?	No

Manuscript

Click here to access/download;Manuscript;Manuscript_clean version.pdf

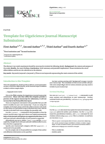

GigaScience, 2023, 1–10

doi: xx.xxxx/xxxx
Manuscript in Preparation
Paper

PAPER

SCSEQ: A web tool for analyzing single-cell RNA-seq data

Shiyu Du[1,*], Pengcheng Sun[1], Li Shen[2] and Jian He[2,*]

[1]Qingdao Institute of Software, College of Computer Science and Technology, China University of Petroleum (East China), Qingdao, 266580, China and [2]State Key Laboratory of Systems Medicine for Cancer, Center for Single-Cell Omics, School of Public Health, Shanghai Jiao Tong University School of Medicine, Shanghai, 200025, China

[*]dushiyu@nimte.ac.cn; jih003@sjtu.edu.cn

Abstract

Single-cell RNA sequencing has emerged as a powerful approach to reveal cellular heterogeneity within biological systems. With the continuous advancement of high-throughput sequencing technologies, studies are generating vast amounts of complex data, posing significant challenge for researchers in effective data processing and analysis. To address this issue, we developed SCSEQ, an interactive web-based bioinformatics analysis platform. This platform enables even users without programming expertise to conveniently process and analyze sequencing data. SCSEQ provides a comprehensive workflow encompassing: data preprocessing, normalization, clustering, dimension reduction, differential expression analysis, cell type identification and downstream analyses. The downstream analysis tasks include gene enrichment analysis, cell-cell communication analysis, copy number variation detection, trajectory inference, and pan-cancer analysis. SCSEQ facilitates information transfer between different workflows, accepts various input formats, and generates graphical and tabular outputs. As a user-friendly platform, we enhance user experience through detailed parameter settings and dynamic interactions. This enables users to precisely regulate research processes and customize result figures. Additionally, we provide comprehensive user manuals to assist with parameter configuration and workflow execution. SCSEQ provides an intuitive and convenient solution for single-cell transcriptome sequencing data analysis. Our platform has successfully completed full-process analyses on real-world data with reliable results, demonstrating its applicability in practical scenarios. The platform is available at `https://ape-closing-tightly.ngrok-free.app`.

Key words: Single-cell RNA sequencing; Data analysis platform; Web-based tool; Machine learning

Introduction

Single-cell sequencing technology, as a major breakthrough in modern life sciences, enables high-throughput sequencing analysis of genomes, transcriptomes, and epigenomes at the individual cell level. This technology goes beyond traditional bulk sequencing by effectively uncovering cellular heterogeneity and precisely delineating gene expression profiles. It provides novel insights and tools for advancing precision medicine and personalized therapy [1]. It can reveal gene expression profiles at single-cell resolution, thereby identifying cell types, states, and intercellular interactions. This provides powerful tools for differential gene expression analysis and alternative splicing studies at the transcriptome level, making it a hot research topic. Currently, single-cell RNA sequencing (scRNA-seq) has become a robust technique for obtaining gene expression profiles at single-cell resolution [2], offering new perspectives for uncovering cellular heterogeneity [3].

Since the pioneering work of Tang et al. [4], which first applied high-throughput sequencing to single cells, the field has rapidly expanded with the development of diverse single-cell omics techniques, such as scWGS [5], scBS-seq [6], and scGRO-seq [7]. However, the rapid accumulation of complex and large-scale datasets poses significant challenges for effective data analysis. In 2021, Zappia and Theis reported that the scRNA tools database had catalogued over a thousand single-cell analysis tools [8]. Among these, two computational ecosystems dominate the single-cell analysis landscape: Seurat [9] for R users and Scanpy [10] for Python users. However, their command-line interfaces and requirement for pro-

Compiled on: January 5, 2026.
Draft manuscript prepared by the author.

Key Points

- SCSEQ provides a no-code pipeline for single-cell transcriptome data analysis from raw data to publication-quality visualizations.
- A highly integrated system that enables flexible fine-tuning and real-time interactive visualization guarantees reliable downstream data analysis.
- Supporting Cell Type Annotation with models trained on User datasets.
- Improving Cell Type Annotation with RAG-enhanced Large Language Models.

gramming expertise pose significant barriers for many researchers lacking extensive coding experience. Moreover, these tools are confined to packages developed in their respective programming languages [11], which hinders the broader adoption of sequencing technologies. In contrast, tools with intuitive graphical user interfaces can significantly facilitate data analysis for researchers and clinicians.

To address this gap, we developed SCSEQ, an integrated and user-friendly web server. It enables comprehensive analyses of single-cell transcriptome data without requiring any programming knowledge. By offering intuitive workflows, extensive parameter customization, and detailed user guidance, SCSEQ aims to make advanced single-cell transcriptome sequencing data analyses accessible to more researchers and clinicians. This platform not only facilitates routine analytical workflows but also provides extensive, specialized downstream functions. It integrates a wide array of benchmark-validated tools—including Seurat, Harmony, CellChat, InferCNV, Monocle, and CellTypist—and offers an expanded suite of downstream analyses such as differential expression, gene enrichment, cell-cell communication, copy number variation, trajectory inference, and pan-cancer analysis. This integrated and updatable design makes SCSEQ a more thorough and versatile solution for single-cell transcriptomic studies, significantly enhancing its value in the rapidly evolving fields of single-cell biology and related disciplines.

The main advantages of SCSEQ are as follows:

1) We have integrated more methods, including benchmark validated methods and state-of-the-art methods, and are able to continuously update and add more excellent methods for users to use. So we are equipped to tackle a broader spectrum of downstream analytical task.
2) We introduce an advanced cell annotation algorithm based on machine learning, which allows users to upload their own datasets, train models, and annotate cells using custom or built-in models.
3) We have explored AI tools in single-cell transcriptomics analysis, integrating existing methods and leveraging large models. A RAG-enhanced large language model can be used to assist in cell-type decision-making.
4) We have designed our platform with numerous adjustable parameters. This allows users to process and analyze their data according to their specific requirements.
5) We offer diverse visualization options. Users can choose what to display and adjust parameters. Real-time adjustments and previews are supported, and visualization results can be saved locally for research or sharing.

Related Work

The computational analysis of scRNA-seq data is dominated by powerful programming frameworks such as Seurat and Scanpy, which provide comprehensive analytical pipelines. However, their command-line interfaces and dependency on specific programming languages restrict their usability for non-specialists. In response, both academic and commercial efforts have led to the development of web-based servers with graphical user interfaces. While platforms such as ASAP [12], ICARUS [13], and CELLAR [14] have matured in handling basic analytical tasks—including data preprocessing, quality control, and cell clustering—their capabilities in advanced downstream analyses remain limited. Particularly for advanced requirements like copy number variation analysis and pan-cancer analysis, most existing platforms offer limited support. Some platforms have begun integrating specialized functions: for instance, ASAP, ICARUS, and CELLAR support cell annotation; OmicStudio [15] incorporates gene set enrichment analysis (GSEA) [16]; SciAp [17] includes trajectory inference; and ezSingleCell [18] offers cell-cell communication analysis. It is worth noting that although these platforms have made valuable attempts in multi omics data analysis, their current analytical capabilities are still insufficient to comprehensively explore single-cell transcriptomic data. There is an urgent need to develop more thorough and professional downstream analysis solutions as a supplement.

These additions are helpful, yet their current analytical capabilities remain insufficient for comprehensive exploration of single-cell transcriptomic data, most servers still leave copy number variation analysis and pan-cancer exploration outside their scope. A single portal that marries routine steps to deep, specialised modules is still missing. SCSEQ was built to close that gap. As mentioned above, we have integrated a large number of advanced tools and can keep the tools updated continuously. These tools enable comprehensive visualization functions and also enable more complete downstream analysis. In addition, our platform provides many adjustable parameters, allowing users to modify parameters to adjust the results before analysis, and adjust visualization parameters to meet personal aesthetic preferences after analysis. For inexperienced users, we provide default parameters to simplify the operation. All parameters and corresponding results are systematically archived, enabling users to track and analyze the source and compare the results of different parameter settings to determine the best configuration. Our implementation and optimization of data processing and visualization will help researchers analyze sequencing data.

Methods

The platform's data analysis workflow is shown in Figure 1. Users start projects by uploading sequencing data. The backend then runs a basic analysis (Fig. 1b) based on the uploaded data and project details. Once the basic analysis is confirmed as accurate, the system moves on to advanced analysis. Since many follow-up tasks rely on cell annotations, we place special emphasis on the cell type identification step. SCSEQ offers multiple annotation methods, and after users confirm the annotation results, downstream analyses can proceed (Fig. 1d). Both basic and advanced analyses are controlled by user-defined parameters. All results are stored for visualization and displayed to users through the frontend interface. Next, we will introduce the methods used in this platform.

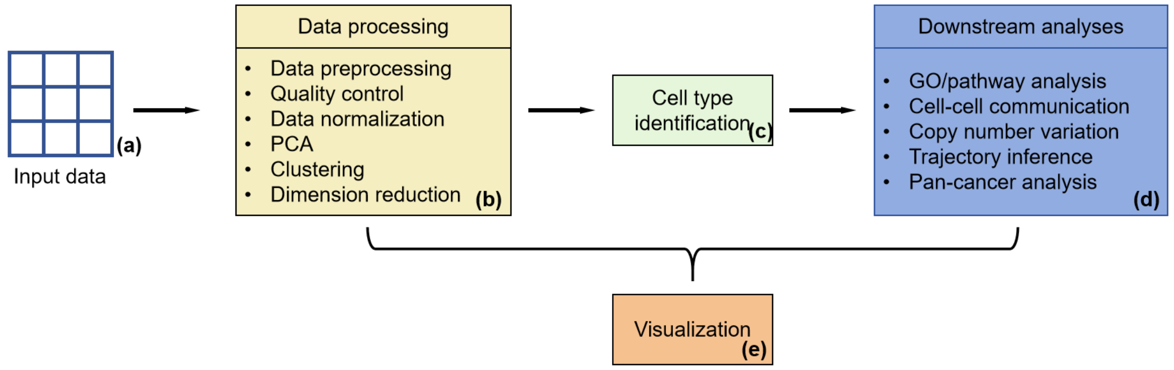

Figure 1. Analysis Process. User-submitted data goes through three key analytical stages: Data Processing, Cell Type Identification, and Downstream Analyses. Visual results from these processes are showcased on the front page.

Bioinformatics software tools

Basic analysis

For the basic analysis phase, we primarily employed methods from the Seurat package. Seurat is an R package tailored for scRNA-seq data analysis. It provides a comprehensive toolkit that enables researchers to extract meaningful biological insights from raw data and reveal cellular heterogeneity.

We performed quality control using Seurat by calculating four key metrics for each cell: the number of detected genes (nFeature_RNA), UMI counts (nCount_RNA), the percentage of mitochondrial genes (percent.mt), and the expression proportion of hemoglobin genes (percent.hb). Following cell filtration, we normalized the data using log-normalization. Highly variable genes were identified using the FindVariableFeatures function, followed by dimensionality reduction through principal component analysis (PCA). Based on the PCA results, we constructed a K-nearest neighbor (KNN) graph and performed cell clustering using the FindNeighbors and FindClusters functions. Finally, we visualized the cell population clusters by further reducing dimensions with either t-SNE or UMAP methods.

Cell type identification

To identify cell types and lay a foundation for downstream analyses, we offer two main annotation methods: SingleR and CellTypist. SingleR is an R package tailored for cell type annotation of scRNA-seq data. It infers cell types for unannotated single-cell data by comparing it to reference datasets. CellTypist is a Python package for annotating single-cell data, employing stochastic gradient descent to train logistic regression classifiers.Users can not only select CellTypist's built-in reference models but also upload annotated data as training sets to develop customized reference models. Relative to built-in models, user-defined models typically exhibit improved compatibility with single-cell datasets, demonstrate superior performance in specific cell populations (e.g., rare or newly discovered cell types), and, when adequate training data are available, provide more accurate annotations.

Additionally, a large language model (LLM) is employed as a supplementary annotation component. For each cluster, the prompt provides the tissue name together with the top 10 marker genes, and the LLM returns candidate cell-type labels along with supporting rationale. Because general-purpose LLMs may have limited bioinformatics knowledge, a retrieval-augmented generation (RAG) strategy is adopted. RAG is an artificial intelligence framework that integrates information retrieval and language generation. By retrieving relevant information from external knowledge base, RAG can provide corresponding reference for large models and enhance the model's performance. Within the scRNA-seq workflow, the widely used single cell transcriptome database (e.g., PanglaoDB) are selected, which is often referred to for manual annotation. Then the data cleaning is completed to ensure that each record contains the tissue name, marker genes, and cell-type label; the curated corpus serves as the external knowledge base. For each cluster pending annotation, relevant records are retrieved and ranked by semantic similarity computed in an embedding space. The ranked results is then supplied to the large language model (LLM) as contextual references, thereby improving performance on the cell-type annotation task. Nevertheless, the accuracy of this approach cannot be guaranteed; results should be regarded as advisory, and adoption remains at the user's discretion. The accuracy of LLM predictions hinges on the marker genes of each cluster. High clustering fidelity boosts prediction reliability. When clusters are accurate and marker genes truly reflect a single cell class, the method's accuracy improves. However, this accuracy isn't guaranteed at present. Results are for reference only, and it's up to users to decide whether to adopt them.

Downstream analyses

Before diving into other analytical tasks, we routinely carry out differential gene expression analysis using the 'FindAllMarkers' and 'FindMarkers' functions from the Seurat package. These functions systematically pinpoint genes that show statistically significant expression differences between specific cell populations or conditions. Following this, we delve into Gene Ontology (GO) Enrichment Analysis. This powerful bioinformatics approach aids researchers in understanding the roles of genes or gene sets across biological processes, molecular functions, and cellular components.

To explore intercellular communication mechanisms, we leverage the CellChatDB reference database. It offers a comprehensive repository of ligand-receptor interactions and signaling pathways. This resource enables us to systematically analyze and visualize cell-cell communication networks within the biological system under investigation.

To detect genomic abnormalities, we conduct Copy Number Variation (CNV) analysis to identify changes in DNA segment copy numbers. In SCSEQ, we use InferCNV, a software package that effectively distinguishes tumor cells from normal cells based on CNV profiles.

Our analytical pipeline also includes two advanced methods: Trajectory Inference and Pan-cancer Analysis. For Trajectory Inference, we use the Monocle package to reconstruct cellular developmental pathways and transitions. For Pan-cancer Analysis, we utilize The Cancer Genome Atlas (TCGA) data to conduct cross-cancer comparative studies. This helps identify common and unique molecular features across different cancer types.

Application development technologies

Front end

The SCSEQ front end is built with Vue, a progressive JavaScript framework. Vue excels in responsive data binding, allowing the page to reflect data changes instantly. This real-time interactivity is ideal for visualizing analytical results and providing immediate feedback. Vue's component-based approach lets us quickly build efficient, visually appealing web applications. This boosts development speed and enhances the user experience.

Back end

The backend of SCSEQ is built with Flask, a lightweight Python web framework. Flask is simple, flexible, and highly extensible. Its streamlined design makes it easy to integrate tools for diverse scenarios and manage complex tasks, which is ideal for scRNA-seq analysis. Flask supports URL parameter parsing and static file serving, ensuring robust request handling and resource access to streamline real-time data exchange.

Database

For database management, we selected MySQL, an open-source relational database management system recognized for its high performance, reliability, and user-friendly features. Widely adopted across applications of varying scales, MySQL serves as the backbone for systematically storing information generated by SCSEQ through three dedicated tables: user table, project table, and task table.

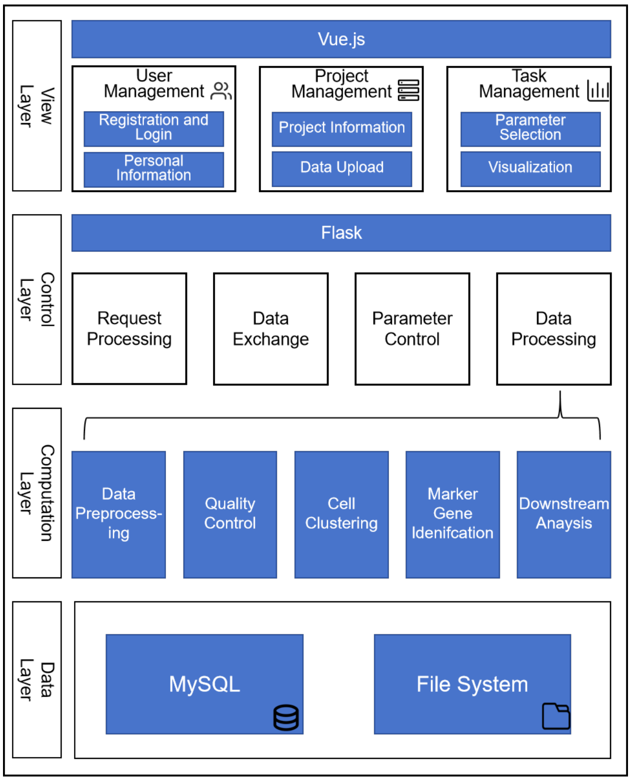

Figure 2. System Overview. SCSEQ is composed of four main components. The View Layer handles user interactions. The Control Layer receives data and parameters from the View Layer and passes them to the Computation Layer to execute algorithms or functions. The Data Layer stores all relevant data and task records.

Implementations

In this section, we introduce the overall architecture of SCSEQ, delve into the architecture and workflow. We offer a thorough explanation of each component's functionality and a comprehensive overview of the entire workflow, while also showcasing the system's user-friendly features.

Building a system that is both efficient and user-friendly is of utmost importance in our research. SCSEQ opts for a front-end–back-end separation architecture. This design pattern is widely used in web application development. It separates the front-end user interface from the back-end service logic, allowing for independent deployment and maintenance. The front-end and back-end can be developed in parallel, which increases development efficiency. Additionally, the use of API interfaces for communication between the front-end and back-end simplifies functional expansion and service upgrades, thereby considerably improving the system's scalability.

Figure 2 provides an overview of our application. SCSEQ primarily consists of four key components: the View Layer, Control Layer, Computation Layer, and Data Layer. The View Layer, implemented as a responsive web interface, serves as the primary interaction portal where researchers can visualize analytical results and configure parameters through intuitive graphical components. The User Management module provides centralized administration of authentication details and account preferences, while the Project Management module shows all projects under the user account . Our platform architecture supports concurrent multi-project workflows, however, each project container maintains strict data isolation, with initial data ingestion restricted exclusively to the project initialization phase to ensure computational reproducibility and version control integrity. Within the Project Management interface, users can comprehensively administer existing projects while also initiating new analytical workflows by submitting requisite data files through our standardized upload protocol. After selecting their desired project, users can configure relevant parameters according to their needs and submit analysis tasks. Upon task completion, the platform will present visualization results on the interface. Users can either view these results online or download them for local storage. All chart results displayed on this platform are available for direct download and saving.

The Control Layer handles interactions between the frontend and backend, as well as some backend functions in the webserver. Its main role is to process requests and data, enabling communication and data exchange between components. This layer receives files and parameter settings from the client interface, directs the Computation Layer to perform customized data processing that meets each user's specific needs, and returns the results to the client interface. For users without programming experience, the Control Layer acts as a capable assistant. It stores uploaded data locally and passes user-defined parameters to the corresponding functions in the Computation Layer for processing and visualization. Researchers can simply interact with intuitive form fields and selection menus on the frontend interface without needing to understand the underlying technical details. After submitting tasks, users can easily monitor progress and receive visualization outputs. This streamlined workflow significantly reduces the technical barriers to biological data analysis. The Computation Layer contains all data processing methods and downstream analysis algorithms. This component integrates several high-quality solutions, which will be detailed in the 'Methods' section.

The Data Layer manages data storage and is split into database and file system parts. For the database, we use MySQL and set up three tables:

- User Table: This stores personal user details, including login credentials.
- Project Table: This holds all project data and links to the User Table via user_id. Besides basic info like project_id, user_id, project_name, and creation_time, it also keeps key analysis data such as species studied, user notes, raw data paths, and work directories. This setup allows users to view their projects in the Project Management interface.

Table 1. A comparative analysis of SCSEQ and current academic web platforms for single-cell analysis tasks.

Web server	Ours	ezSingle-Cell	ICARUS	ASAP	alona	Cellar	SCiAp	NASQAR	SCTK	Asc-Seurat
Clustering and dimension reduction	✓	✓	✓	✓	✓	✓	✓	✓	✓	✓
Cell type identification	✓	✓	✓	✓	×	✓	✓	×	✓	×
GO/pathway analysis	✓	✓	✓	✓	×	✓	✓	✓	✓	✓
Cell-cell communication	✓	✓	×	×	×	×	×	×	×	×
Copy number variation	✓	×	×	×	×	×	×	×	×	×
Trajectory inference	✓	×	×	×	×	×	×	×	×	×
Pan-cancer analysis	✓	×	×	×	×	×	×	×	×	×

Note: ✓ and × denote whether the web server supports the functionality.

- Task Table: This records all tasks linked to projects via project_id. It includes details like task_id, task_type, parameters, result paths, submission time, and jobid for tracking. In SCSEQ, this table does three main things: lets users review all past tasks, logs parameters for each task, and notes where task results are stored. These features make analyses traceable and reproducible, boosting SCSEQ's usefulness.

The file system stores various files generated during platform operations. Strategies vary by file type:

- Raw Files: These are user-uploaded sequencing data, often in matrix format and storage-heavy. They're mainly used for preprocessing, after which data is stored as RDS files.
- Intermediate Files: Generated during analysis (e.g., InferCNV creates intermediate files at each step). As they're regenerated with each task, we don't specially retain them.
- Result Files: These include charts and visualization data, which take up little space. We keep all result files, naming them with task type and timestamp for easy comparison. Users can delete task records via the frontend, which also deletes corresponding results. Deleting a project removes all its files.

Results and discussion

Benchmarking SCSEQ against existing platforms

SCSEQ specializes in single-cell transcriptomics analysis, completing a complete data analysis pipeline. Throughout the analytical process, we have integrated multiple excellent methods and provided numerous analysis tools. Using these methods, users can perform basic data processing as well as advanced downstream analyses. Inspired by ezSingleCell, the integrated tools and their comparisons with other similar platforms are shown in Table 1. In comparison,SCSEQ offers a broader range of advanced downstream analytical functionalities. For these tools, we provide default parameters while also supporting user-defined parameter inputs, ultimately obtaining high-quality visualization results.

Advantages of SCSEQ

Benefiting from the reasonable system architecture described previously, SCSEQ has numerous user-friendly and practical features:

Multi-task concurrency and flexible task scheduling: The system extends task management functionality on the project page(Fig. 3), implemented as a dialog interface. This allows users to view comprehensive task information including task type, relevant parameters, execution status, and submission time - all queried from the Task Table in the database. The operation panel allows users to review results or delete records for any task. This design improves task scheduling. Users can submit tasks, shut down their computers temporarily, and check results later. It also helps in planning follow-up analysis workflows. Additionally, the system supports multi-task concurrency. Users can run multiple projects at the same time without waiting for current tasks to finish. They can monitor all task statuses and access results through the unified task management dialog.

Comprehensive parameter configuration: SCSEQ incorporates a wide range of adjustable parameters, enabling users to precisely control the analytical process. Thanks to the well-designed task table, SCSEQ can save the parameters configured by users for each submitted task. This allows for retrospective analysis and comparative evaluation of results obtained with different parameter settings. Additionally, the system provides default parameter sets optimized for most analytical workflows, simplifying user operations. For visualization outputs, parameter controls are implemented, allowing users to customize elements such as font sizes and axis ranges, thus achieving personalized data visualization.

Interactive visualization and diverse visualization outputs: To enhance user experience and deliver richer insights, SCSEQ uses interactive visualizations with ECharts components. For example, in marker gene dotplots, users can hover over nodes to see detailed information like 'Cell Type', 'Gene Type', 'avg exp', and 'pct1' values. SCSEQ also offers various visualization types, including violin plots, scatter plots, bar charts, dotplots, circle plots, heatmaps, box plots, and forest plots. These options allow users to select the most intuitive representation for their analytical tasks.

Real-time updates and immediate feedback: The platform's cloud tools allow real-time updates to existing results. For example, users can choose to display the number of genes per cell population in the marker gene results, and the chart will update immediately. For cloud analyses requiring computation to produce results, users need to wait until the analysis task completes to view the visualization outcomes. Once a task is complete, the results are instantly visible on the current page. This design minimizes debugging time for users, aids in understanding how parameters affect outcomes, helps identify more suitable parameters, and ultimately leads to better results.

Data Analysis

To showcase SCSEQ's capabilities, we analyzed a dataset of 2,700 peripheral blood mononuclear cells (PBMCs)[19] and present the results (Figs. 4,5).

After uploading the data, we conducted basic analysis using

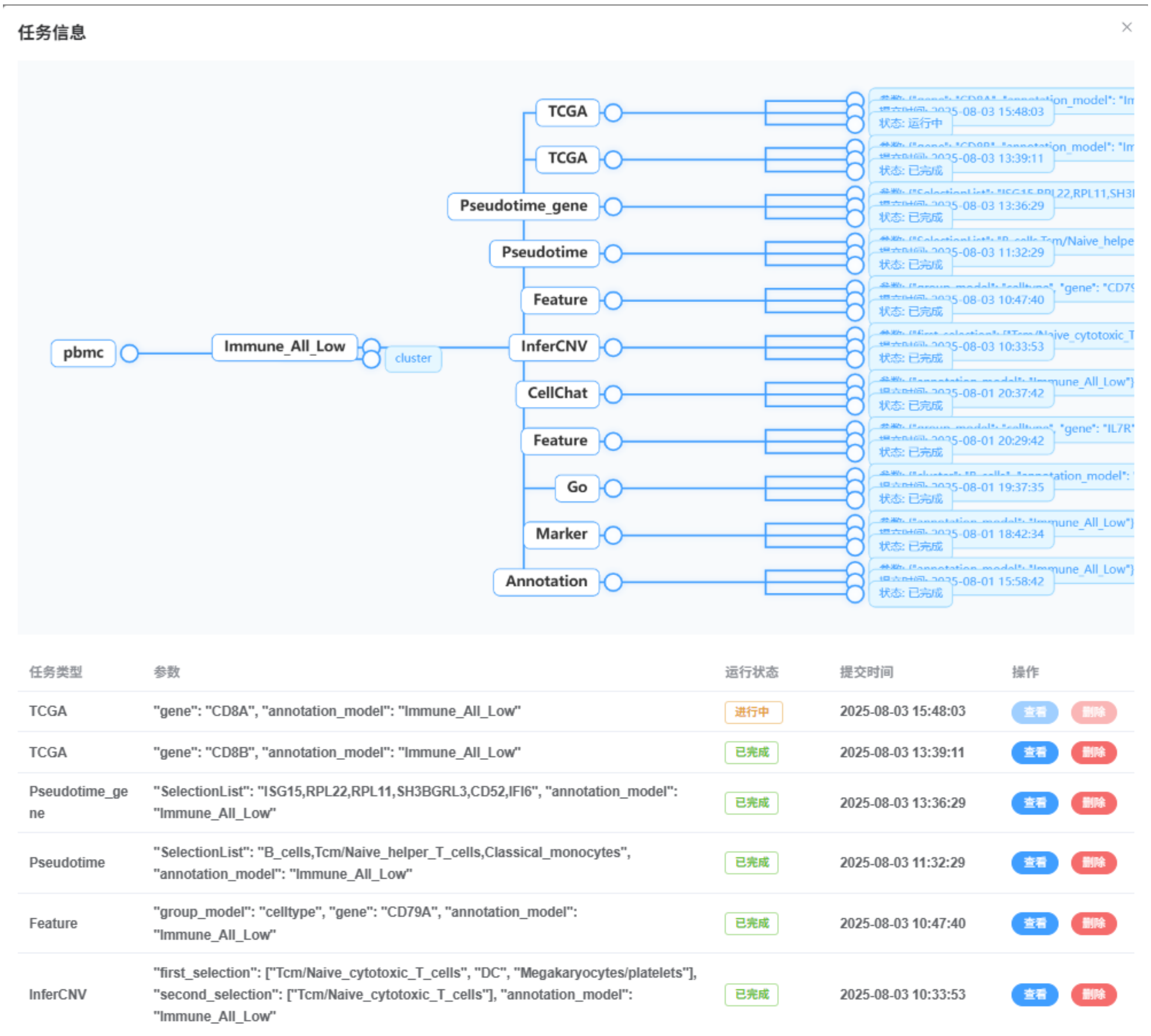

任务类型	参数	运行状态	提交时间	操作
TCGA	"gene": "CD8A", "annotation_model": "Immune_All_Low"	进行中	2025-08-03 15:48:03	查看 删除
TCGA	"gene": "CD8B", "annotation_model": "Immune_All_Low"	已完成	2025-08-03 13:39:11	查看 删除
Pseudotime_gene	"SelectionList": "ISG15,RPL22,RPL11,SH3BGRL3,CD52,IFI6", "annotation_model": "Immune_All_Low"	已完成	2025-08-03 13:36:29	查看 删除
Pseudotime	"SelectionList": "B_cells,Tcm/Naive_helper_T_cells,Classical_monocytes", "annotation_model": "Immune_All_Low"	已完成	2025-08-03 11:32:29	查看 删除
Feature	"group_model": "celltype", "gene": "CD79A", "annotation_model": "Immune_All_Low"	已完成	2025-08-03 10:47:40	查看 删除
InferCNV	"first_selection": ["Tcm/Naive_cytotoxic_T_cells", "DC", "Megakaryocytes/platelets"], "second_selection": ["Tcm/Naive_cytotoxic_T_cells"], "annotation_model": "Immune_All_Low"	已完成	2025-08-03 10:33:53	查看 删除

Figure 3. Task Information Dialog. This page displays task information under the current project and build a tree view based on the annotation method.

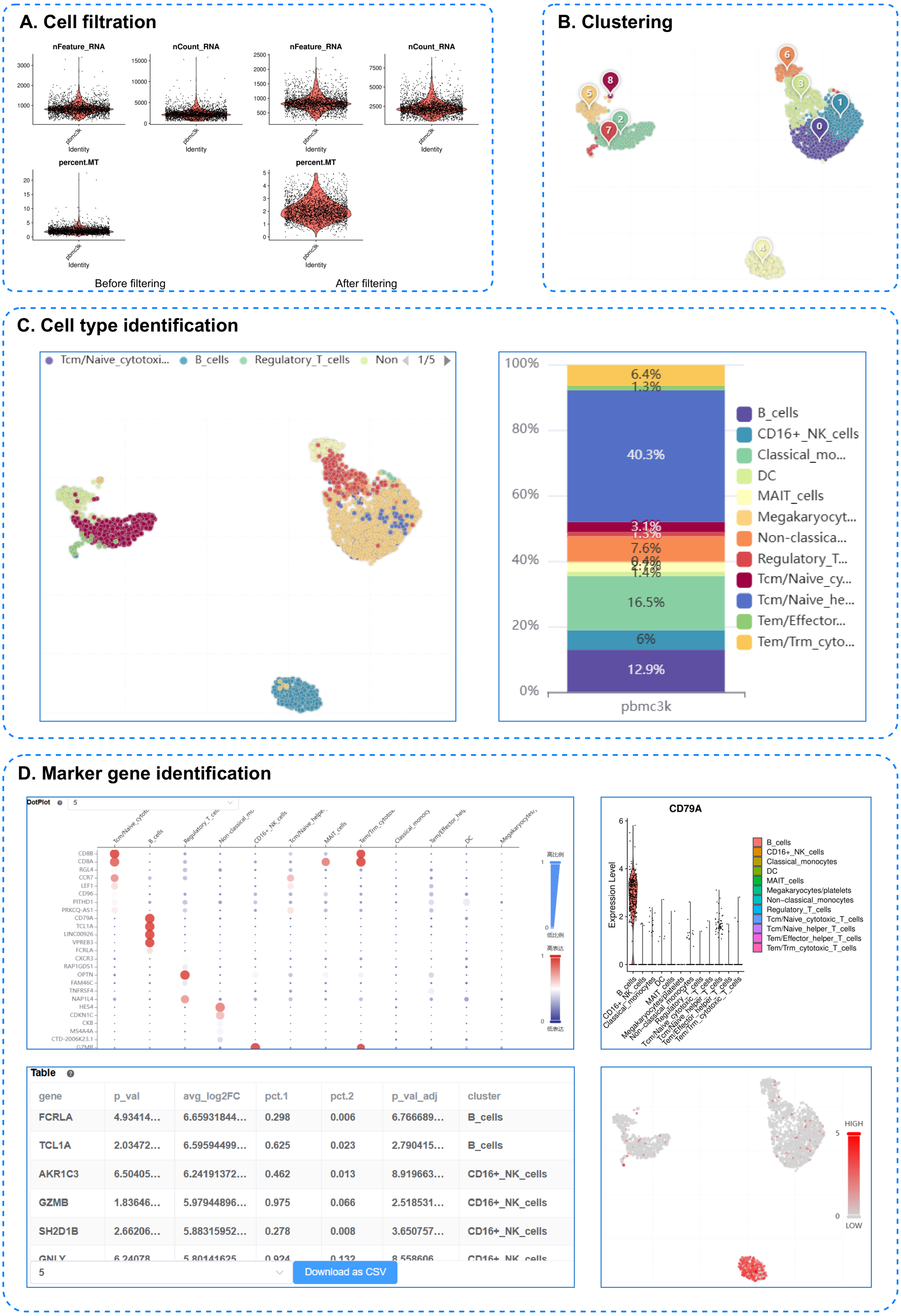

Figure 4. Basic analysis and cell annotation. **(A)** Comparison before and after cell filtration; **(B)** Visualization of clustering results; **(C)** CellTypist annotation results and cell proportion plots; **(D)** Marker gene tables and dotplot visualizations. Distribution of individual genes across all cell populations.

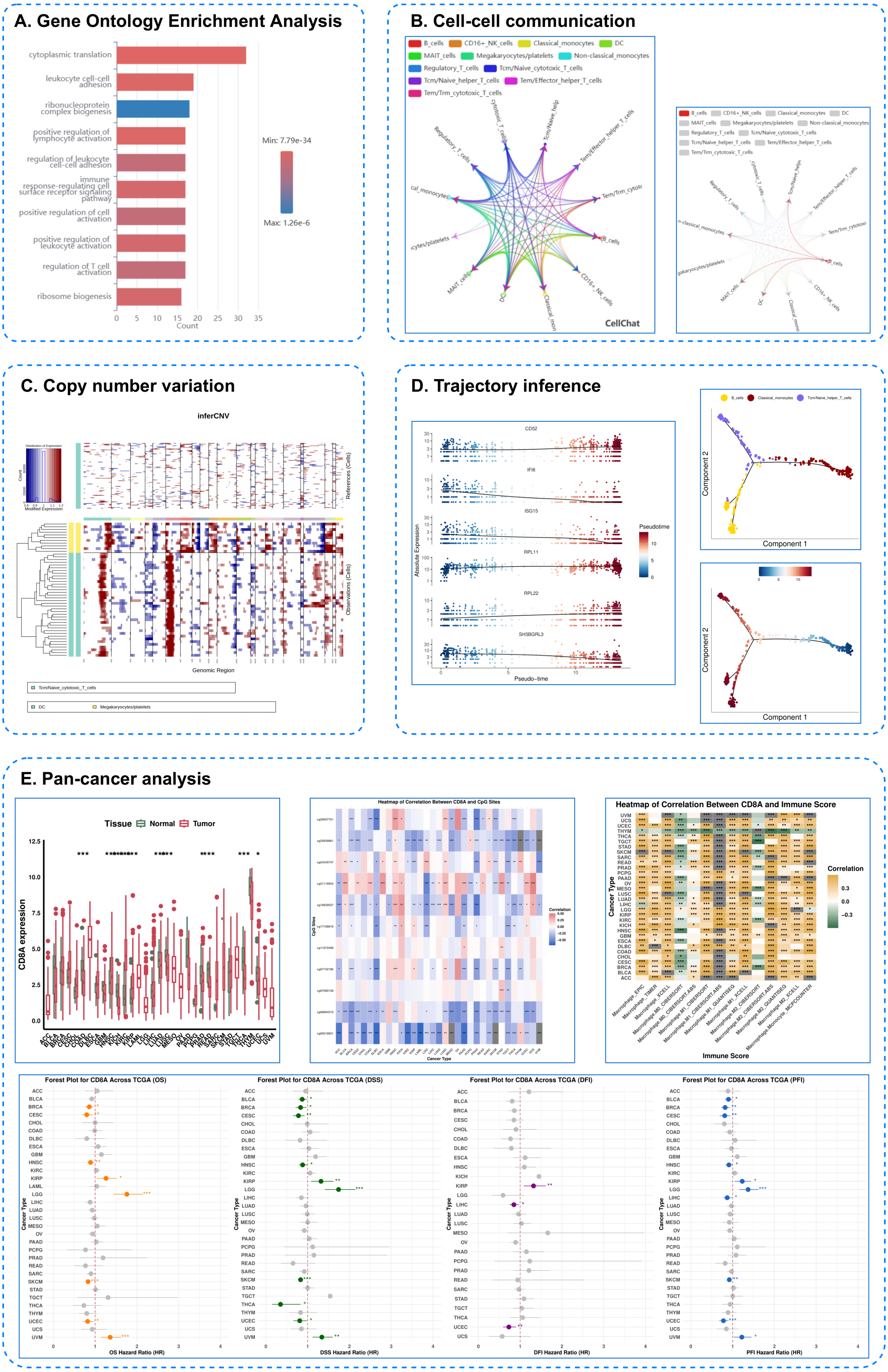

Figure 5. Advanced analysis. **(A)** GO enrichment results of B cells; **(B)** Cell-cell communication analysis using CellChatDB; **(C)** Copy number variation analysis using InferCNV; **(D)** Cell and gene expression dynamics along trajectories; **(D)** Pan-cancer analysis using TCGA data.

default parameters from Seurat's official documentation. SCSEQ initially displayed the overall data distribution and provided quality control metrics such as nFeature_RNA, nCount_RNA, and percent.mt (percent.hb was optional and not used in this case). We used a violin chart to visualize the data before and after cell filtering (Fig. 4A). The subsequent steps involved log-normalization, identification of highly variable genes, data scaling, and PCA. Based on the PCA results, we constructed a KNN graph and performed clustering at a resolution of 0.5 (default), resulting in 9 distinct clusters (Fig. 4B).

In the process of cell annotation, we used celltype's "Immune_All_Low" reference set. This identified 12 cell populations, including B cells, CD16+ NK cells, Classical monocytes, DCs, MAIT cells, Megakaryocytes/platelets, Non-classical monocytes, Regulatory T cells, Tcm/Naive cytotoxic T cells, Tcm/Naive helper T cells, Tem/Effector helper T cells, and Tem/Trm cytotoxic T cells. We also show the proportion of each cell type in the total cell population (Fig. 4C). To aid downstream analysis, we calculated marker genes for each annotated cluster and present them in tables and dot plots (Fig. 4D). Additionally, users can select specific genes to examine their expression patterns across all cell populations, as demonstrated by the scatter plot and violin plot in Fig. 4C.

After basic analysis, advanced analysis can be performed. We first performed GO Enrichment Analysis. We selected biological processes related to B cells and visualized the top 10 terms by 'Count' value using bar plots (Fig. 5A). For cell-cell communication analysis, we employed the CellChatDB database to examine ligand-receptor pairs (Fig. 5B). As shown in the left panel of Fig. 5B, all cell types are selectable, so Users can also selectively examine interaction results between specific cell types of interest and other cells. For instance, in the right panel of Figure 5B, we selected B cells for visualization.

Copy number variation analysis was conducted using InferCNV, with Tcm/Naive_cytotoxic_T_cells as reference set alongside DC and Megakaryocytes/platelets populations (Fig. 5C).For trajectory inference analysis, we selected the three most abundant cell populations: B_cells, Tcm/Naive_helper_T_cells, and Classical_monocytes. The platform also supports examining gene expression dynamics along trajectories (Fig. 5D). We specifically analyzed the temporal expression patterns of ISG15, RPL22, RPL11, SH3BGRL3, CD52, and IFI6 genes.

Pan-cancer analysis of CD8A gene was performed using TCGA tools, with results presented through box plots, heatmaps, and forest plots (Fig. 5E). The boxplot shows CD8A expression differences between tumor (red) and normal tissues (green) across multiple cancer types. Two heatmaps highlight: (1) links between CD8A expression and CpG methylation, indicating epigenetic regulation of T cell infiltration and treatment potential; and (2) relationships between CD8A expression and macrophage immune scores across cancer types, shedding light on immune cell interactions and tumor diversity. Additionally, a forest plot presents survival analysis results for target gene expression data across different cancer types in TCGA.

Conclusion

SCSEQ consolidates an unusually broad slice of single-cell science into one coherent web workspace. Cell-type labelling, gene enrichment analysis, cell-cell communication analysis, copy number variation detection, trajectory inference, and pan-cancer analysis and high-resolution visualisation are no longer scattered across different packages; they are accessible through a single menu, callable with a few clicks and parameter sliders. Behind the interface sits a containerised backend that wraps rigorously benchmarked tools—Seurat, Harmony [20], CellChat [21], InferCNV, Monocle [22], and CellTypist [23]—so that every step inherits the statistical robustness of the original code while gaining the reproducibility of version-tracked workflows. Users can therefore move from raw count matrix to publication-grade figures without installing interpreters, managing dependencies or editing scripts. The platform's parameter logging and real time visualization further transform exploratory analysis into an intuitive feedback loop: adjust parameters, observe results, and export satisfactory images.

Looking forward, , the platform's impact on bioinformatics will continue to grow, and we are extending the architecture along two complementary axes. First, our platform currently focuses solely on single-cell RNA sequencing and does not support Spatial Transcriptomics or single-cell Multiomics (scMultiomics), representing one of our key directions for future development. In addition, the artificial intelligence approaches applied in our cell type identification module have demonstrated excellent performance, which inspires us to incorporate more artificial intelligence analysis tools in future updates to further enhance the performance of SCSEQ and contribute to the rapid development of single-cell biology and related disciplines.

Availability of source code and requirements

- Project name: SCSEQ
- Project home page: `https://github.com/knight-spc/SCSEQ`
- Operating system(s): Platform independent
- Programming language: Python, Vue
- Other requirements: Anaconda (Recommended)
- License: GPL-3.0 license

Data Availability

The dataset used to illustrate SCSEQ's performance consists of 2,700 Peripheral Blood Mononuclear Cells (PBMC) sequenced on the Illumina NextSeq 500. Raw data and the processed count matrix are available from the 10x Genomics [19].

Declaration

Declaration of competing interest

We declare that we have no financial and personal relationships with other people or organizations that can inappropriately influence our work.

Declaration of generative AI and AI-assisted technologies in the manuscript preparation process

During the preparation of this work the author used Kimi K1.5 in order to translate and polish the manuscript text. After using this tool, the author reviewed and edited the content as needed and take full responsibility for the content of the published article.

Acknowledgements

This work was supported by the China University of Petroleum (East China) Discipline Start-up Fund [grant number 1500-05Y23080001].This work was supported by grants from National Key Technologies Research and Development Program of China(2022YFD2101503) of J.H.

References

1. Altschuler SJ, Wu LF. Cellular heterogeneity: Do differences make a difference? Cell 2010;141(4):559–563.

2. Chen G, Ning B, Shi T. Single-cell RNA-seq technologies and related computational data analysis. Front Genet 2019;10:317.
3. Haque A, Engel J, Teichmann SA, Lönnberg T. A practical guide to single-cell RNA-sequencing for biomedical research and clinical applications. Genome Med 2017;9(1):75.
4. Tang F, Barbacioru C, Wang Y, Nordman E, Lee C, Xu N, et al. mRNA-Seq whole-transcriptome analysis of a single cell. Nat Methods 2009;6(5):377–382.
5. Zachariadis V, Cheng H, Andrews N, et al. A highly scalable method for joint whole-genome sequencing and gene-expression profiling of single cells. Mol Cell 2020;80(3):541–553.e5.
6. Smallwood SA, Lee HJ, Angermueller C, et al. Single-cell genome-wide bisulfite sequencing for assessing epigenetic heterogeneity. Nat Methods 2014;11(8):817–820.
7. Mahat DB, Tippens ND, Martin-Rufino JD, et al. Single-cell nascent RNA sequencing unveils coordinated global transcription. Nature 2024;631:216–223.
8. Zappia L, Theis FJ. Over 1000 tools reveal trends in the single-cell RNA-seq analysis landscape. Genome Biol 2021;22:301.
9. Butler A, Hoffman P, Smibert P, Papalexi E, Satija R. Integrating single-cell transcriptomic data across different conditions, technologies, and species. Nat Biotechnol 2018;36:411–420.
10. Wolf FA, Angerer P, Theis FJ. SCANPY: Large-scale single-cell gene expression data analysis. Genome Biol 2018;19:15.
11. Luecken MD, Theis FJ. Current best practices in single-cell RNA-seq analysis: A tutorial. Mol Syst Biol 2019;15:e8746.
12. Gardeux V, David FPA, Shajkofci A, Schwalie PC, Deplancke B. ASAP: A web-based platform for the analysis and interactive visualization of single-cell RNA-seq data. Bioinformatics 2017;33:3123–3125.
13. Jiang A, Lehnert K, You L, Snell RG. ICARUS, an interactive web server for single cell RNA-seq analysis. Nucleic Acids Res 2022;50(W1):W427–W433.
14. Hasanaj E, Wang J, Sarathi A, Ding J, Bar-Joseph Z. Interactive single-cell data analysis using Cellar. Nat Commun 2022;13:1998.
15. Lyu F, Han F, Ge C, Mao W, Chen L, Hu H, et al. OmicStudio: A composable bioinformatics cloud platform with real-time feedback that can generate high-quality graphs for publication. iMeta 2023;2:e85.
16. Subramanian A, Tamayo P, Mootha VK, et al. Gene set enrichment analysis: A knowledge-based approach for interpreting genome-wide expression profiles. Proc Natl Acad Sci USA 2005;102:15545–15550.
17. Moreno P, Huang N, Manning JR, et al. User-friendly, scalable tools and workflows for single-cell RNA-seq analysis. Nat Methods 2021;18:327–328.
18. Sethi R, Ang KS, Li M, Long Y, Ling J, Chen J. ezSingleCell: An integrated one-stop single-cell and spatial omics analysis platform for bench scientists. Nat Commun 2024;15:5600.
19. PBMCs from a healthy donor (v3, 3k cells, 150×150);. https://cf.10xgenomics.com/samples/cell/pbmc3k/pbmc3k_filtered_gene_bc_matrices.tar.gz. Accessed: 2025-11-14. 10x Genomics.
20. Korsunsky I, Millard N, Fan J, et al. Fast, sensitive and accurate integration of single-cell data with Harmony. Nat Methods 2019;16:1289–1296.
21. Jin S, Guerrero-Juarez CF, Zhang L, et al. Inference and analysis of cell-cell communication using CellChat. Nat Commun 2021;12:1088.
22. Qiu X, Mao Q, Tang Y, et al. Reversed graph embedding resolves complex single-cell trajectories. Nat Methods 2017;14:979–982.
23. Domínguez Conde C, et al. Cross-tissue immune cell analysis reveals tissue-specific features in humans. Science 2022;376:eabl5197.

LaTeX - Main Document (TeX file) Click here to access/download;Manuscript;Manuscript.tex

GigaScience, 2023, 1–10

doi: xx.xxxx/xxxx
Manuscript in Preparation
Paper

PAPER

SCSEQ: A web tool for analyzing single-cell RNA-seq data

Shiyu Du[1,*], Pengcheng Sun[1], Li Shen[2] and Jian He[2,*]

[1]Qingdao Institute of Software, College of Computer Science and Technology, China University of Petroleum (East China), Qingdao, 266580, China and [2]State Key Laboratory of Systems Medicine for Cancer, Center for Single-Cell Omics, School of Public Health, Shanghai Jiao Tong University School of Medicine, Shanghai, 200025, China

*dushiyu@nimte.ac.cn; jih003@sjtu.edu.cn

Abstract

Single-cell RNA sequencing has emerged as a powerful approach to reveal cellular heterogeneity within biological systems. With the continuous advancement of high-throughput sequencing technologies, studies are generating vast amounts of complex data, posing significant challenge for researchers in effective data processing and analysis. To address this issue, we developed SCSEQ, an interactive web-based bioinformatics analysis platform. This platform enables even users without programming expertise to conveniently process and analyze sequencing data. SCSEQ provides a comprehensive workflow encompassing: data preprocessing, normalization, clustering, dimension reduction, differential expression analysis, cell type identification and downstream analyses. The downstream analysis tasks include gene enrichment analysis, cell-cell communication analysis, copy number variation detection, trajectory inference, and pan-cancer analysis. SCSEQ facilitates information transfer between different workflows, accepts various input formats, and generates graphical and tabular outputs. As a user-friendly platform, we enhance user experience through detailed parameter settings and dynamic interactions. This enables users to precisely regulate research processes and customize result figures. Additionally, we provide comprehensive user manuals to assist with parameter configuration and workflow execution. SCSEQ provides an intuitive and convenient solution for single-cell transcriptome sequencing data analysis. Our platform has successfully completed full-process analyses on real-world data with reliable results, demonstrating its applicability in practical scenarios. The platform is available at `https://ape-closing-tightly.ngrok-free.app`.

Key words: Single-cell RNA sequencing; Data analysis platform; Web-based tool; Machine learning

Introduction

Single-cell sequencing technology, as a major breakthrough in modern life sciences, enables high-throughput sequencing analysis of genomes, transcriptomes, and epigenomes at the individual cell level. This technology goes beyond traditional bulk sequencing by effectively uncovering cellular heterogeneity and precisely delineating gene expression profiles. It provides novel insights and tools for advancing precision medicine and personalized therapy [1]. It can reveal gene expression profiles at single-cell resolution, thereby identifying cell types, states, and intercellular interactions. This provides powerful tools for differential gene expression analysis and alternative splicing studies at the transcriptome level, making it a hot research topic. Currently, single-cell RNA sequencing (scRNA-seq) has become a robust technique for obtaining gene expression profiles at single-cell resolution [2], offering new perspectives for uncovering cellular heterogeneity [3].

Since the pioneering work of Tang et al. [4], which first applied high-throughput sequencing to single cells, the field has rapidly expanded with the development of diverse single-cell omics techniques, such as scWGS [5], scBS-seq [6], and scGRO-seq [7]. However, the rapid accumulation of complex and large-scale datasets poses significant challenges for effective data analysis. In 2021, Zappia and Theis reported that the scRNA tools database had catalogued over a thousand single-cell analysis tools [8]. Among these, two computational ecosystems dominate the single-cell analysis landscape: Seurat [9] for R users and Scanpy [10] for Python users. However, their command-line interfaces and requirement for pro-

Compiled on: December 29, 2025.
Draft manuscript prepared by the author.

Key Points

- SCSEQ provides a no-code pipeline for single-cell transcriptome data analysis from raw data to publication-quality visualizations.
- A highly integrated system that enables flexible fine-tuning and real-time interactive visualization guarantees reliable downstream data analysis.
- Supporting Cell Type Annotation with models trained on User datasets.
- Improving Cell Type Annotation with RAG-enhanced Large Language Models.

gramming expertise pose significant barriers for many researchers lacking extensive coding experience. Moreover, these tools are confined to packages developed in their respective programming languages [11], which hinders the broader adoption of sequencing technologies. In contrast, tools with intuitive graphical user interfaces can significantly facilitate data analysis for researchers and clinicians.

To address this gap, we developed SCSEQ, an integrated and user-friendly web server. It enables comprehensive analyses of single-cell transcriptome data without requiring any programming knowledge. By offering intuitive workflows, extensive parameter customization, and detailed user guidance, SCSEQ aims to make advanced single-cell transcriptome sequencing data analyses accessible to more researchers and clinicians. This platform not only facilitates routine analytical workflows but also provides extensive, specialized downstream functions. It integrates a wide array of benchmark-validated tools—including Seurat, Harmony, CellChat, InferCNV, Monocle, and CellTypist—and offers an expanded suite of downstream analyses such as differential expression, gene enrichment, cell-cell communication, copy number variation, trajectory inference, and pan-cancer analysis. This integrated and updatable design makes SCSEQ a more thorough and versatile solution for single-cell transcriptomic studies, significantly enhancing its value in the rapidly evolving fields of single-cell biology and related disciplines.

The main advantages of SCSEQ are as follows:

1) We have integrated more methods, including benchmark validated methods and state-of-the-art methods, and are able to continuously update and add more excellent methods for users to use. So we are equipped to tackle a broader spectrum of downstream analytical task.
2) We introduce an advanced cell annotation algorithm based on machine learning, which allows users to upload their own datasets, train models, and annotate cells using custom or built-in models.
3) We have explored AI tools in single-cell transcriptomics analysis, integrating existing methods and leveraging large models. A RAG-enhanced large language model can be used to assist in cell-type decision-making.
4) We have designed our platform with numerous adjustable parameters. This allows users to process and analyze their data according to their specific requirements.
5) We offer diverse visualization options. Users can choose what to display and adjust parameters. Real-time adjustments and previews are supported, and visualization results can be saved locally for research or sharing.

Related Work

The computational analysis of scRNA-seq data is dominated by powerful programming frameworks such as Seurat and Scanpy, which provide comprehensive analytical pipelines. However, their command-line interfaces and dependency on specific programming languages restrict their usability for non-specialists. In response, both academic and commercial efforts have led to the development of web-based servers with graphical user interfaces. While platforms such as ASAP [12], ICARUS [13], and CELLAR [14] have matured in handling basic analytical tasks—including data preprocessing, quality control, and cell clustering—their capabilities in advanced downstream analyses remain limited. Particularly for advanced requirements like copy number variation analysis and pan-cancer analysis, most existing platforms offer limited support. Some platforms have begun integrating specialized functions: for instance, ASAP, ICARUS, and CELLAR support cell annotation; OmicStudio [15] incorporates gene set enrichment analysis (GSEA) [16]; SciAp [17] includes trajectory inference; and ezSingleCell [18] offers cell-cell communication analysis. It is worth noting that although these platforms have made valuable attempts in multi omics data analysis, their current analytical capabilities are still insufficient to comprehensively explore single-cell transcriptomic data. There is an urgent need to develop more thorough and professional downstream analysis solutions as a supplement.

These additions are helpful, yet their current analytical capabilities remain insufficient for comprehensive exploration of single-cell transcriptomic data, most servers still leave copy number variation analysis and pan-cancer exploration outside their scope. A single portal that marries routine steps to deep, specialised modules is still missing. SCSEQ was built to close that gap. As mentioned above, we have integrated a large number of advanced tools and can keep the tools updated continuously. These tools enable comprehensive visualization functions and also enable more complete downstream analysis. In addition, our platform provides many adjustable parameters, allowing users to modify parameters to adjust the results before analysis, and adjust visualization parameters to meet personal aesthetic preferences after analysis. For inexperienced users, we provide default parameters to simplify the operation. All parameters and corresponding results are systematically archived, enabling users to track and analyze the source and compare the results of different parameter settings to determine the best configuration. Our implementation and optimization of data processing and visualization will help researchers analyze sequencing data.

Methods

The platform's data analysis workflow is shown in Figure 1. Users start projects by uploading sequencing data. The backend then runs a basic analysis (Fig. 1b) based on the uploaded data and project details. Once the basic analysis is confirmed as accurate, the system moves on to advanced analysis. Since many follow-up tasks rely on cell annotations, we place special emphasis on the cell type identification step. SCSEQ offers multiple annotation methods, and after users confirm the annotation results, downstream analyses can proceed (Fig. 1d). Both basic and advanced analyses are controlled by user-defined parameters. All results are stored for visualization and displayed to users through the frontend interface. Next, we will introduce the methods used in this platform.

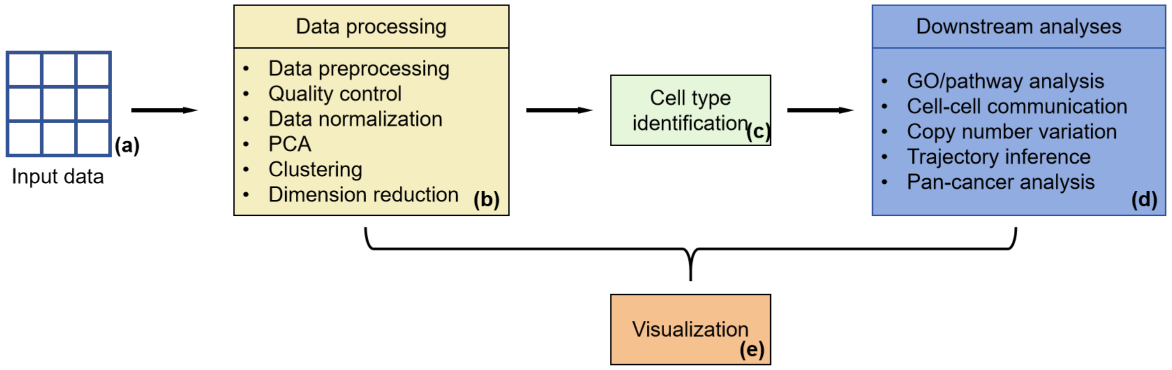

Figure 1. Analysis Process. User-submitted data goes through three key analytical stages: Data Processing, Cell Type Identification, and Downstream Analyses. Visual results from these processes are showcased on the front page.

Bioinformatics software tools

Basic analysis

For the basic analysis phase, we primarily employed methods from the Seurat package. Seurat is an R package tailored for scRNA-seq data analysis. It provides a comprehensive toolkit that enables researchers to extract meaningful biological insights from raw data and reveal cellular heterogeneity.

We performed quality control using Seurat by calculating four key metrics for each cell: the number of detected genes (nFeature_RNA), UMI counts (nCount_RNA), the percentage of mitochondrial genes (percent.mt), and the expression proportion of hemoglobin genes (percent.hb). Following cell filtration, we normalized the data using log-normalization. Highly variable genes were identified using the FindVariableFeatures function, followed by dimensionality reduction through principal component analysis (PCA). Based on the PCA results, we constructed a K-nearest neighbor (KNN) graph and performed cell clustering using the FindNeighbors and FindClusters functions. Finally, we visualized the cell population clusters by further reducing dimensions with either t-SNE or UMAP methods.

Cell type identification

To identify cell types and lay a foundation for downstream analyses, we offer two main annotation methods: SingleR and CellTypist. SingleR is an R package tailored for cell type annotation of scRNA-seq data. It infers cell types for unannotated single-cell data by comparing it to reference datasets. CellTypist is a Python package for annotating single-cell data, employing stochastic gradient descent to train logistic regression classifiers.Users can not only select CellTypist's built-in reference models but also upload annotated data as training sets to develop customized reference models. Relative to built-in models, user-defined models typically exhibit improved compatibility with single-cell datasets, demonstrate superior performance in specific cell populations (e.g., rare or newly discovered cell types), and, when adequate training data are available, provide more accurate annotations.

Additionally, a large language model (LLM) is employed as a supplementary annotation component. For each cluster, the prompt provides the tissue name together with the top 10 marker genes, and the LLM returns candidate cell-type labels along with supporting rationale. Because general-purpose LLMs may have limited bioinformatics knowledge, a retrieval-augmented generation (RAG) strategy is adopted. RAG is an artificial intelligence framework that integrates information retrieval and language generation. By retrieving relevant information from external knowledge base, RAG can provide corresponding reference for large models and enhance the model's performance. Within the scRNA-seq workflow, the widely used single cell transcriptome database (e.g., PanglaoDB) are selected, which is often referred to for manual annotation. Then the data cleaning is completed to ensure that each record contains the tissue name, marker genes, and cell-type label; the curated corpus serves as the external knowledge base. For each cluster pending annotation, relevant records are retrieved and ranked by semantic similarity computed in an embedding space. The ranked results is then supplied to the large language model (LLM) as contextual references, thereby improving performance on the cell-type annotation task. Nevertheless, the accuracy of this approach cannot be guaranteed; results should be regarded as advisory, and adoption remains at the user's discretion. The accuracy of LLM predictions hinges on the marker genes of each cluster. High clustering fidelity boosts prediction reliability. When clusters are accurate and marker genes truly reflect a single cell class, the method's accuracy improves. However, this accuracy isn't guaranteed at present. Results are for reference only, and it's up to users to decide whether to adopt them.

Downstream analyses

Before diving into other analytical tasks, we routinely carry out differential gene expression analysis using the 'FindAllMarkers' and 'FindMarkers' functions from the Seurat package. These functions systematically pinpoint genes that show statistically significant expression differences between specific cell populations or conditions. Following this, we delve into Gene Ontology (GO) Enrichment Analysis. This powerful bioinformatics approach aids researchers in understanding the roles of genes or gene sets across biological processes, molecular functions, and cellular components.

To explore intercellular communication mechanisms, we leverage the CellChatDB reference database. It offers a comprehensive repository of ligand-receptor interactions and signaling pathways. This resource enables us to systematically analyze and visualize cell-cell communication networks within the biological system under investigation.

To detect genomic abnormalities, we conduct Copy Number Variation (CNV) analysis to identify changes in DNA segment copy numbers. In SCSEQ, we use InferCNV, a software package that effectively distinguishes tumor cells from normal cells based on CNV profiles.

Our analytical pipeline also includes two advanced methods: Trajectory Inference and Pan-cancer Analysis. For Trajectory Inference, we use the Monocle package to reconstruct cellular developmental pathways and transitions. For Pan-cancer Analysis, we utilize The Cancer Genome Atlas (TCGA) data to conduct cross-cancer comparative studies. This helps identify common and unique molecular features across different cancer types.

Application development technologies

Front end

The SCSEQ front end is built with Vue, a progressive JavaScript framework. Vue excels in responsive data binding, allowing the page to reflect data changes instantly. This real-time interactivity is ideal for visualizing analytical results and providing immediate feedback. Vue's component-based approach lets us quickly build efficient, visually appealing web applications. This boosts development speed and enhances the user experience.

Back end

The backend of SCSEQ is built with Flask, a lightweight Python web framework. Flask is simple, flexible, and highly extensible. Its streamlined design makes it easy to integrate tools for diverse scenarios and manage complex tasks, which is ideal for scRNA-seq analysis. Flask supports URL parameter parsing and static file serving, ensuring robust request handling and resource access to streamline real-time data exchange.

Database

For database management, we selected MySQL, an open-source relational database management system recognized for its high performance, reliability, and user-friendly features. Widely adopted across applications of varying scales, MySQL serves as the backbone for systematically storing information generated by SCSEQ through three dedicated tables: user table, project table, and task table.

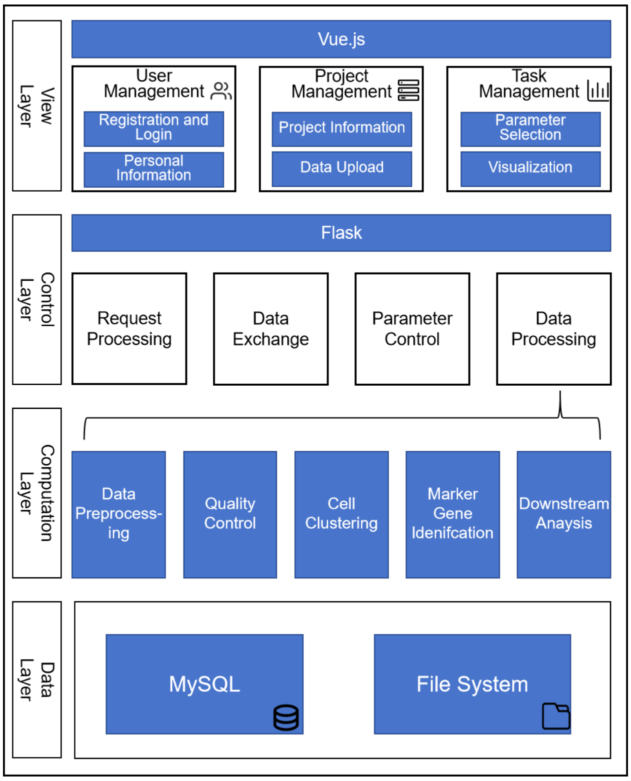

Figure 2. System Overview. SCSEQ is composed of four main components. The View Layer handles user interactions. The Control Layer receives data and parameters from the View Layer and passes them to the Computation Layer to execute algorithms or functions. The Data Layer stores all relevant data and task records.

Implementations

In this section, we introduce the overall architecture of SCSEQ, delve into the architecture and workflow. We offer a thorough explanation of each component's functionality and a comprehensive overview of the entire workflow, while also showcasing the system's user-friendly features.

Building a system that is both efficient and user-friendly is of utmost importance in our research. SCSEQ opts for a front-end–back-end separation architecture. This design pattern is widely used in web application development. It separates the front-end user interface from the back-end service logic, allowing for independent deployment and maintenance. The front-end and back-end can be developed in parallel, which increases development efficiency. Additionally, the use of API interfaces for communication between the front-end and back-end simplifies functional expansion and service upgrades, thereby considerably improving the system's scalability.

Figure 2 provides an overview of our application. SCSEQ primarily consists of four key components: the View Layer, Control Layer, Computation Layer, and Data Layer. The View Layer, implemented as a responsive web interface, serves as the primary interaction portal where researchers can visualize analytical results and configure parameters through intuitive graphical components. The User Management module provides centralized administration of authentication details and account preferences, while the Project Management module shows all projects under the user account . Our platform architecture supports concurrent multi-project workflows, however, each project container maintains strict data isolation, with initial data ingestion restricted exclusively to the project initialization phase to ensure computational reproducibility and version control integrity. Within the Project Management interface, users can comprehensively administer existing projects while also initiating new analytical workflows by submitting requisite data files through our standardized upload protocol. After selecting their desired project, users can configure relevant parameters according to their needs and submit analysis tasks. Upon task completion, the platform will present visualization results on the interface. Users can either view these results online or download them for local storage. All chart results displayed on this platform are available for direct download and saving.

The Control Layer handles interactions between the frontend and backend, as well as some backend functions in the webserver. Its main role is to process requests and data, enabling communication and data exchange between components. This layer receives files and parameter settings from the client interface, directs the Computation Layer to perform customized data processing that meets each user's specific needs, and returns the results to the client interface. For users without programming experience, the Control Layer acts as a capable assistant. It stores uploaded data locally and passes user-defined parameters to the corresponding functions in the Computation Layer for processing and visualization. Researchers can simply interact with intuitive form fields and selection menus on the frontend interface without needing to understand the underlying technical details. After submitting tasks, users can easily monitor progress and receive visualization outputs. This streamlined workflow significantly reduces the technical barriers to biological data analysis. The Computation Layer contains all data processing methods and downstream analysis algorithms. This component integrates several high-quality solutions, which will be detailed in the 'Methods' section.

The Data Layer manages data storage and is split into database and file system parts. For the database, we use MySQL and set up three tables:

- User Table: This stores personal user details, including login credentials.
- Project Table: This holds all project data and links to the User Table via user_id. Besides basic info like project_id, user_id, project_name, and creation_time, it also keeps key analysis data such as species studied, user notes, raw data paths, and work directories. This setup allows users to view their projects in the Project Management interface.

Table 1. A comparative analysis of SCSEQ and current academic web platforms for single-cell analysis tasks.

Web server	Ours	ezSingle-Cell	ICARUS	ASAP	alona	Cellar	SCiAp	NASQAR	SCTK	Asc-Seurat
Clustering and dimension reduction	✓	✓	✓	✓	✓	✓	✓	✓	✓	✓
Cell type identification	✓	✓	✓	✓	×	✓	✓	×	✓	×
GO/pathway analysis	✓	✓	✓	✓	×	✓	✓	✓	✓	✓
Cell-cell communication	✓	✓	×	×	×	×	×	×	×	×
Copy number variation	✓	×	×	×	×	×	×	×	×	×
Trajectory inference	✓	×	×	×	×	×	×	×	×	×
Pan-cancer analysis	✓	×	×	×	×	×	×	×	×	×

Note: ✓ and × denote whether the web server supports the functionality.

- Task Table: This records all tasks linked to projects via project_id. It includes details like task_id, task_type, parameters, result paths, submission time, and jobid for tracking. In SCSEQ, this table does three main things: lets users review all past tasks, logs parameters for each task, and notes where task results are stored. These features make analyses traceable and reproducible, boosting SCSEQ's usefulness.

The file system stores various files generated during platform operations. Strategies vary by file type:

- Raw Files: These are user-uploaded sequencing data, often in matrix format and storage-heavy. They're mainly used for preprocessing, after which data is stored as RDS files.
- Intermediate Files: Generated during analysis (e.g., InferCNV creates intermediate files at each step). As they're regenerated with each task, we don't specially retain them.
- Result Files: These include charts and visualization data, which take up little space. We keep all result files, naming them with task type and timestamp for easy comparison. Users can delete task records via the frontend, which also deletes corresponding results. Deleting a project removes all its files.

Results

Benchmarking SCSEQ against existing platforms

SCSEQ specializes in single-cell transcriptomics analysis, completing a complete data analysis pipeline. Throughout the analytical process, we have integrated multiple excellent methods and provided numerous analysis tools. Using these methods, users can perform basic data processing as well as advanced downstream analyses. Inspired by ezSingleCell, the integrated tools and their comparisons with other similar platforms are shown in Table 1. In comparison,SCSEQ offers a broader range of advanced downstream analytical functionalities. For these tools, we provide default parameters while also supporting user-defined parameter inputs, ultimately obtaining high-quality visualization results.

Advantages of SCSEQ

Benefiting from the reasonable system architecture described previously, SCSEQ has numerous user-friendly and practical features:

Multi-task concurrency and flexible task scheduling: The system extends task management functionality on the project page(Fig. 3), implemented as a dialog interface. This allows users to view comprehensive task information including task type, relevant parameters, execution status, and submission time - all queried from the Task Table in the database. The operation panel allows users to review results or delete records for any task. This design improves task scheduling. Users can submit tasks, shut down their computers temporarily, and check results later. It also helps in planning follow-up analysis workflows. Additionally, the system supports multi-task concurrency. Users can run multiple projects at the same time without waiting for current tasks to finish. They can monitor all task statuses and access results through the unified task management dialog.

Comprehensive parameter configuration: SCSEQ incorporates a wide range of adjustable parameters, enabling users to precisely control the analytical process. Thanks to the well-designed task table, SCSEQ can save the parameters configured by users for each submitted task. This allows for retrospective analysis and comparative evaluation of results obtained with different parameter settings. Additionally, the system provides default parameter sets optimized for most analytical workflows, simplifying user operations. For visualization outputs, parameter controls are implemented, allowing users to customize elements such as font sizes and axis ranges, thus achieving personalized data visualization.

Interactive visualization and diverse visualization outputs: To enhance user experience and deliver richer insights, SCSEQ uses interactive visualizations with ECharts components. For example, in marker gene dotplots, users can hover over nodes to see detailed information like 'Cell Type', 'Gene Type', 'avg exp', and 'pct1' values. SCSEQ also offers various visualization types, including violin plots, scatter plots, bar charts, dotplots, circle plots, heatmaps, box plots, and forest plots. These options allow users to select the most intuitive representation for their analytical tasks.

Real-time updates and immediate feedback: The platform's cloud tools allow real-time updates to existing results. For example, users can choose to display the number of genes per cell population in the marker gene results, and the chart will update immediately. For cloud analyses requiring computation to produce results, users need to wait until the analysis task completes to view the visualization outcomes. Once a task is complete, the results are instantly visible on the current page. This design minimizes debugging time for users, aids in understanding how parameters affect outcomes, helps identify more suitable parameters, and ultimately leads to better results.

Results and discussion

To showcase SCSEQ's capabilities, we analyzed a dataset of 2,700 peripheral blood mononuclear cells (PBMCs) and present the results (Figs. 4,5).

After uploading the data, we conducted basic analysis using

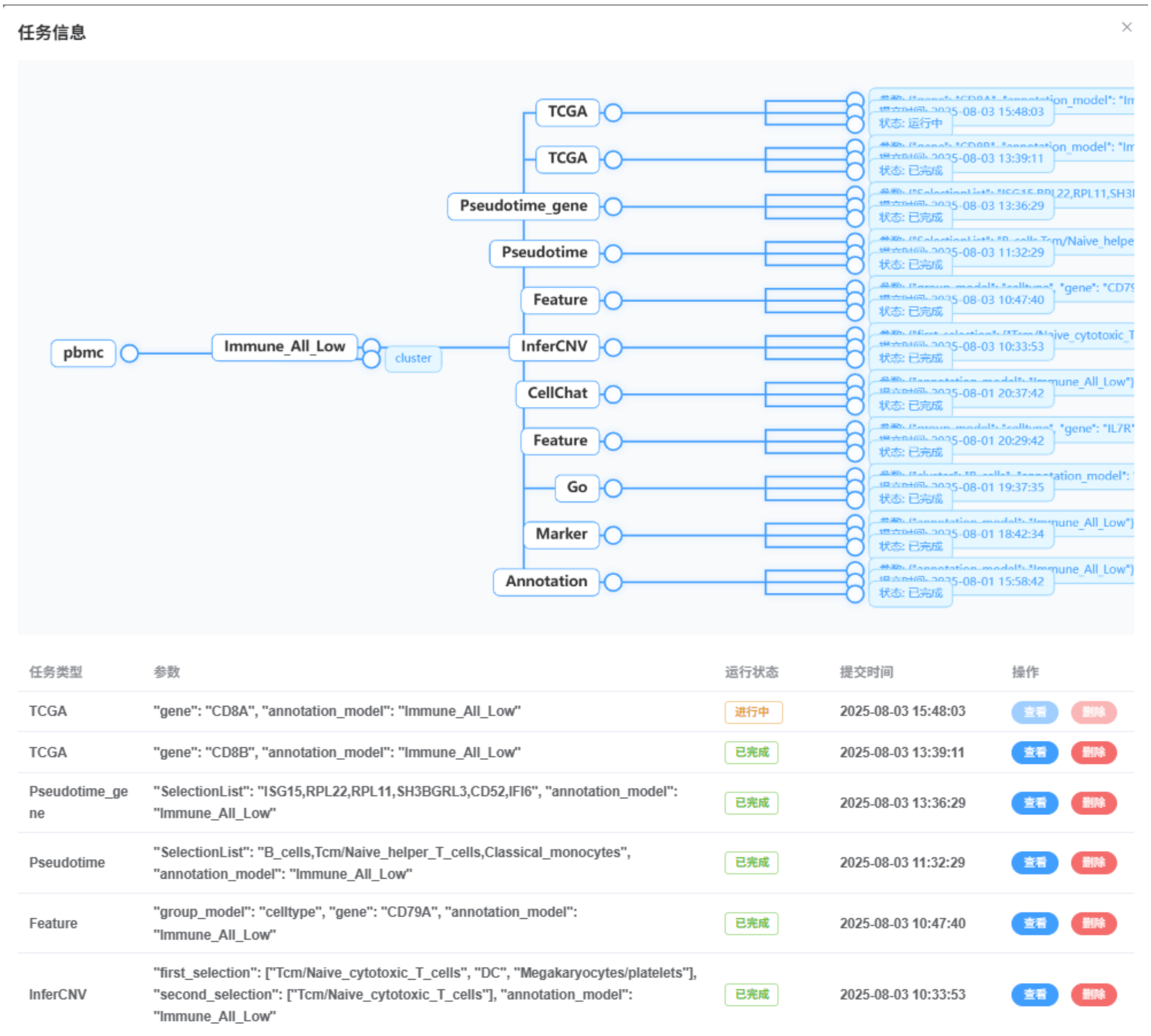

Figure 3. Task Information Dialog. This page displays task information under the current project and build a tree view based on the annotation method.

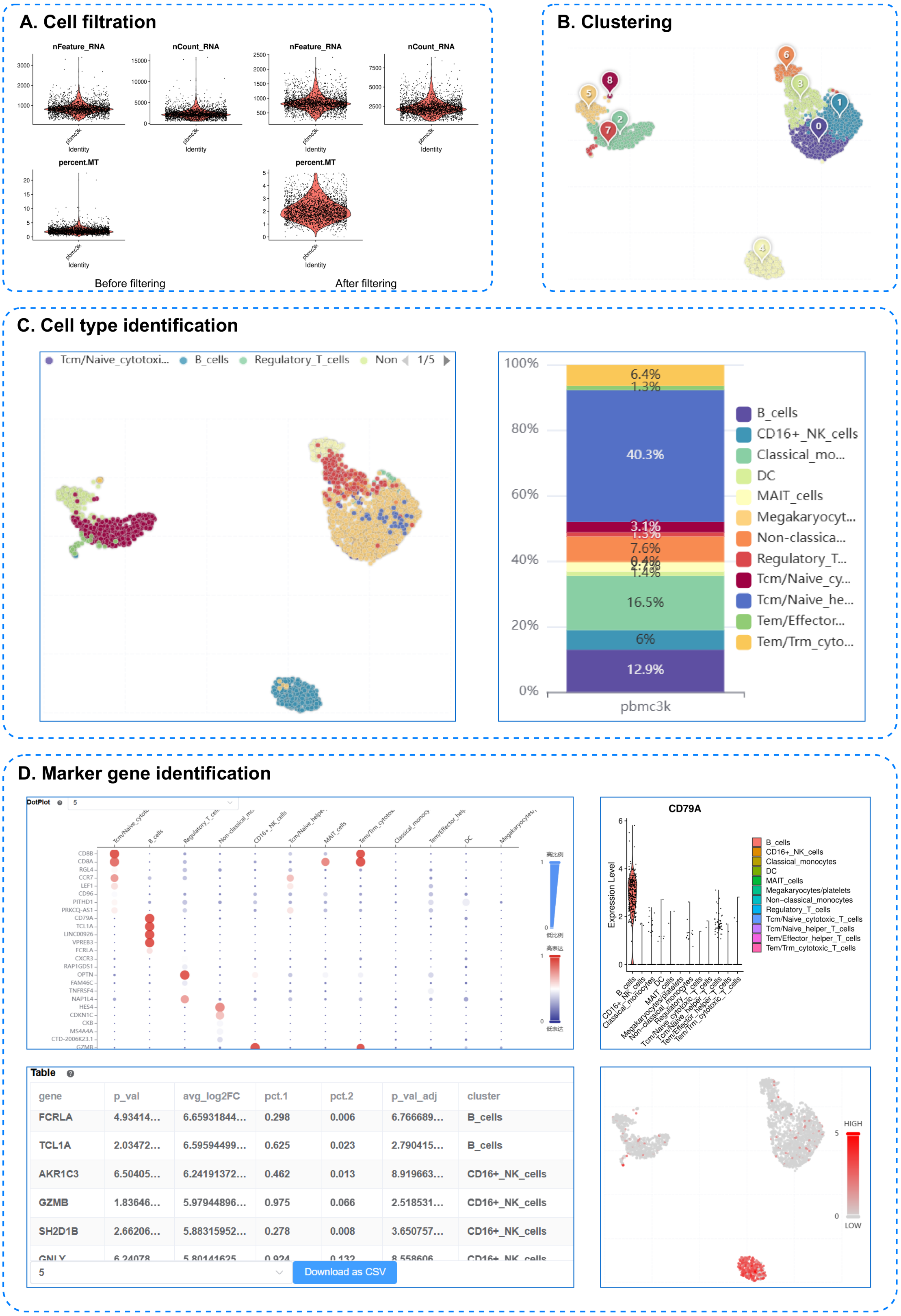

Figure 4. Basic analysis and cell annotation. **(A)** Comparison before and after cell filtration; **(B)** Visualization of clustering results; **(C)** CellTypist annotation results and cell proportion plots; **(D)** Marker gene tables and dotplot visualizations. Distribution of individual genes across all cell populations.

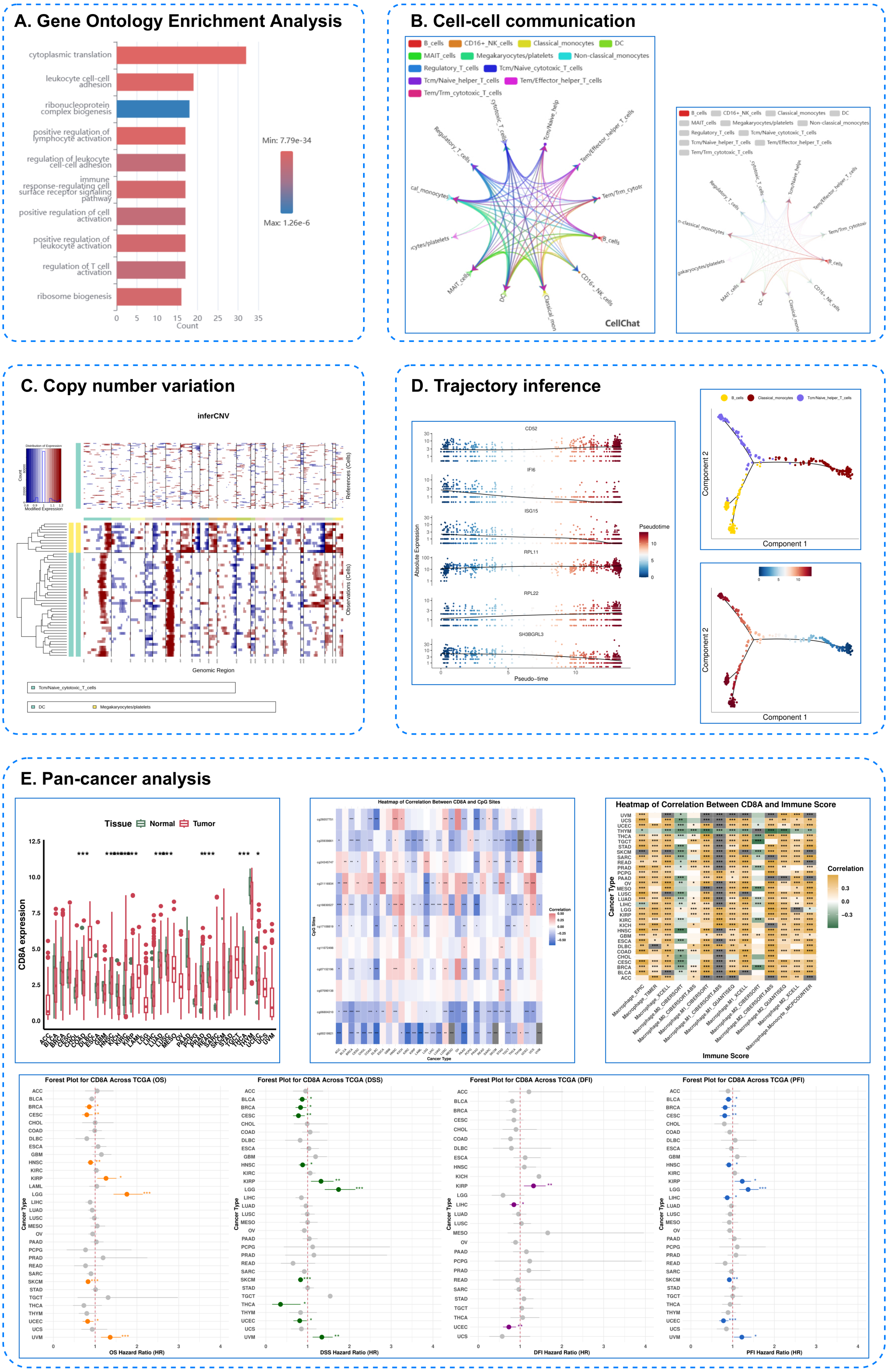

Figure 5. Advanced analysis. **(A)** GO enrichment results of B cells; **(B)** Cell-cell communication analysis using CellChatDB; **(C)** Copy number variation analysis using InferCNV; **(D)** Cell and gene expression dynamics along trajectories; **(D)** Pan-cancer analysis using TCGA data.

default parameters from Seurat's official documentation. SCSEQ initially displayed the overall data distribution and provided quality control metrics such as nFeature_RNA, nCount_RNA, and percent.mt (percent.hb was optional and not used in this case). We used a violin chart to visualize the data before and after cell filtering (Fig. 4A). The subsequent steps involved log-normalization, identification of highly variable genes, data scaling, and PCA. Based on the PCA results, we constructed a KNN graph and performed clustering at a resolution of 0.5 (default), resulting in 9 distinct clusters (Fig. 4B).

In the process of cell annotation, we used celltype's "Immune_All_Low" reference set. This identified 12 cell populations, including B cells, CD16+ NK cells, Classical monocytes, DCs, MAIT cells, Megakaryocytes/platelets, Non-classical monocytes, Regulatory T cells, Tcm/Naive cytotoxic T cells, Tcm/Naive helper T cells, Tem/Effector helper T cells, and Tem/Trm cytotoxic T cells. We also show the proportion of each cell type in the total cell population (Fig. 4C). To aid downstream analysis, we calculated marker genes for each annotated cluster and present them in tables and dot plots (Fig. 4D). Additionally, users can select specific genes to examine their expression patterns across all cell populations, as demonstrated by the scatter plot and violin plot in Fig. 4C.

After basic analysis, advanced analysis can be performed. We first performed GO Enrichment Analysis. We selected biological processes related to B cells and visualized the top 10 terms by 'Count' value using bar plots (Fig. 5A). For cell-cell communication analysis, we employed the CellChatDB database to examine ligand-receptor pairs (Fig. 5B). As shown in the left panel of Fig. 5B, all cell types are selectable, so Users can also selectively examine interaction results between specific cell types of interest and other cells. For instance, in the right panel of Figure 5B, we selected B cells for visualization.

Copy number variation analysis was conducted using InferCNV, with Tcm/Naive_cytotoxic_T_cells as reference set alongside DC and Megakaryocytes/platelets populations (Fig. 5C).For trajectory inference analysis, we selected the three most abundant cell populations: B_cells, Tcm/Naive_helper_T_cells, and Classical_monocytes. The platform also supports examining gene expression dynamics along trajectories (Fig. 5D). We specifically analyzed the temporal expression patterns of ISG15, RPL22, RPL11, SH3BGRL3, CD52, and IFI6 genes.

Pan-cancer analysis of CD8A gene was performed using TCGA tools, with results presented through box plots, heatmaps, and forest plots (Fig. 5E). The boxplot shows CD8A expression differences between tumor (red) and normal tissues (green) across multiple cancer types. Two heatmaps highlight: (1) links between CD8A expression and CpG methylation, indicating epigenetic regulation of T cell infiltration and treatment potential; and (2) relationships between CD8A expression and macrophage immune scores across cancer types, shedding light on immune cell interactions and tumor diversity. Additionally, a forest plot presents survival analysis results for target gene expression data across different cancer types in TCGA.

Conclusion

SCSEQ consolidates an unusually broad slice of single-cell science into one coherent web workspace. Cell-type labelling, gene enrichment analysis, cell-cell communication analysis, copy number variation detection, trajectory inference, and pan-cancer analysis and high-resolution visualisation are no longer scattered across different packages; they are accessible through a single menu, callable with a few clicks and parameter sliders. Behind the interface sits a containerised backend that wraps rigorously benchmarked tools—Seurat, Harmony [19], CellChat [20], InferCNV, Monocle [21], and CellTypist [22]—so that every step inherits the statistical robustness of the original code while gaining the reproducibility of version-tracked workflows. Users can therefore move from raw count matrix to publication-grade figures without installing interpreters, managing dependencies or editing scripts. The platform's parameter logging and real time visualization further transform exploratory analysis into an intuitive feedback loop: adjust parameters, observe results, and export satisfactory images.

Looking forward, , the platform's impact on bioinformatics will continue to grow, and we are extending the architecture along two complementary axes. First, our platform currently focuses solely on single-cell RNA sequencing and does not support Spatial Transcriptomics or single-cell Multiomics (scMultiomics), representing one of our key directions for future development. In addition, the artificial intelligence approaches applied in our cell type identification module have demonstrated excellent performance, which inspires us to incorporate more artificial intelligence analysis tools in future updates to further enhance the performance of SCSEQ and contribute to the rapid development of single-cell biology and related disciplines.

Availability of source code and requirements

- Project name: SCSEQ
- Project home page: `https://github.com/knight-spc/SCSEQ`
- Operating system(s): Platform independent
- Programming language: Python, Vue
- Other requirements: Anaconda (Recommended)
- License: GPL-3.0 license

Data Availability

The dataset used to illustrate SCSEQ's performance consists of 2,700 Peripheral Blood Mononuclear Cells (PBMC) sequenced on the Illumina NextSeq 500. Raw data and the processed count matrix are available from the 10x Genomics [23].

Declaration

Declaration of competing interest

We declare that we have no financial and personal relationships with other people or organizations that can inappropriately influence our work.

Declaration of generative AI and AI-assisted technologies in the manuscript preparation process

During the preparation of this work the author used Kimi K1.5 in order to translate and polish the manuscript text. After using this tool, the author reviewed and edited the content as needed and take full responsibility for the content of the published article.

Acknowledgements

This work was supported by the China University of Petroleum (East China) Discipline Start-up Fund [grant number 1500-05Y23080001].This work was supported by grants from National Key Technologies Research and Development Program of China(2022YFD2101503) of J.H.

References

1. Altschuler SJ, Wu LF. Cellular heterogeneity: Do differences make a difference? Cell 2010;141(4):559–563.

2. Chen G, Ning B, Shi T. Single-cell RNA-seq technologies and related computational data analysis. Front Genet 2019;10:317.
3. Haque A, Engel J, Teichmann SA, Lönnberg T. A practical guide to single-cell RNA-sequencing for biomedical research and clinical applications. Genome Med 2017;9(1):75.
4. Tang F, Barbacioru C, Wang Y, Nordman E, Lee C, Xu N, et al. mRNA-Seq whole-transcriptome analysis of a single cell. Nat Methods 2009;6(5):377–382.
5. Zachariadis V, Cheng H, Andrews N, et al. A highly scalable method for joint whole-genome sequencing and gene-expression profiling of single cells. Mol Cell 2020;80(3):541–553.e5.
6. Smallwood SA, Lee HJ, Angermueller C, et al. Single-cell genome-wide bisulfite sequencing for assessing epigenetic heterogeneity. Nat Methods 2014;11(8):817–820.
7. Mahat DB, Tippens ND, Martin-Rufino JD, et al. Single-cell nascent RNA sequencing unveils coordinated global transcription. Nature 2024;631:216–223.
8. Zappia L, Theis FJ. Over 1000 tools reveal trends in the single-cell RNA-seq analysis landscape. Genome Biol 2021;22:301.
9. Butler A, Hoffman P, Smibert P, Papalexi E, Satija R. Integrating single-cell transcriptomic data across different conditions, technologies, and species. Nat Biotechnol 2018;36:411–420.
10. Wolf FA, Angerer P, Theis FJ. SCANPY: Large-scale single-cell gene expression data analysis. Genome Biol 2018;19:15.
11. Luecken MD, Theis FJ. Current best practices in single-cell RNA-seq analysis: A tutorial. Mol Syst Biol 2019;15:e8746.
12. Gardeux V, David FPA, Shajkofci A, Schwalie PC, Deplancke B. ASAP: A web-based platform for the analysis and interactive visualization of single-cell RNA-seq data. Bioinformatics 2017;33:3123–3125.
13. Jiang A, Lehnert K, You L, Snell RG. ICARUS, an interactive web server for single cell RNA-seq analysis. Nucleic Acids Res 2022;50(W1):W427–W433.
14. Hasanaj E, Wang J, Sarathi A, Ding J, Bar-Joseph Z. Interactive single-cell data analysis using Cellar. Nat Commun 2022;13:1998.
15. Lyu F, Han F, Ge C, Mao W, Chen L, Hu H, et al. OmicStudio: A composable bioinformatics cloud platform with real-time feedback that can generate high-quality graphs for publication. iMeta 2023;2:e85.
16. Subramanian A, Tamayo P, Mootha VK, et al. Gene set enrichment analysis: A knowledge-based approach for interpreting genome-wide expression profiles. Proc Natl Acad Sci USA 2005;102:15545–15550.
17. Moreno P, Huang N, Manning JR, et al. User-friendly, scalable tools and workflows for single-cell RNA-seq analysis. Nat Methods 2021;18:327–328.
18. Sethi R, Ang KS, Li M, Long Y, Ling J, Chen J. ezSingleCell: An integrated one-stop single-cell and spatial omics analysis platform for bench scientists. Nat Commun 2024;15:5600.
19. Korsunsky I, Millard N, Fan J, et al. Fast, sensitive and accurate integration of single-cell data with Harmony. Nat Methods 2019;16:1289–1296.
20. Jin S, Guerrero-Juarez CF, Zhang L, et al. Inference and analysis of cell-cell communication using CellChat. Nat Commun 2021;12:1088.
21. Qiu X, Mao Q, Tang Y, et al. Reversed graph embedding resolves complex single-cell trajectories. Nat Methods 2017;14:979–982.
22. Domínguez Conde C, et al. Cross-tissue immune cell analysis reveals tissue-specific features in humans. Science 2022;376:eabl5197.
23. PBMCs from a healthy donor (v3, 3k cells, 150×150);. https://www.10xgenomics.com/datasets, https://www.10xgenomics.com/datasets. Accessed: 2025-11-14. 10x Genomics.

Cover Letter
Click here to access/download;Personal Cover;Cover Letter.docx

Dear Editors,

On behalf of all co-authors, I am pleased to submit our manuscript entitled “SCSEQ: A web tool for analyzing single-cell RNA-seq data” for consideration in GigaScience.

Single-cell RNA-sequencing outputs are growing faster than most wet-lab teams can analyse. Existing platforms either demand expert coding or stop short of advanced tasks. We therefore built SCSEQ, a knowledge-driven, cloud-native pipeline that wraps benchmarked tools into an intuitive Vue/Flask framework. A retrieval-augmented LLM module draws on curated single-cell corpora to suggest cell-type labels, while a MySQL-backed task manager logs every parameter set and result for full reproducibility. Users can launch downstream analyses from the same dashboard, adjust visual aesthetics in real time, and compare outputs across parameter sets without re-uploading data. All plots are downloadable.

The highlights of this manuscript are:
• SCSEQ provides a no-code pipeline for single-cell transcriptome data analysis from raw data to publication-quality visualizations.
• A highly integrated system that enables flexible fine-tuning and real-time interactive visualization guarantees reliable downstream data analysis.
• Supporting cell type annotation with models trained on user datasets.
• Improving cell type annotation with RAG-enhanced large language models.

Our work is a strong fit for GigaScience. SCSEQ integrates a large amount of single-cell transcriptome sequencing data into a unified pipeline, promoting accessibility and reproducibility. Its use artificial Intelligence to enhance annotation accuracy exemplifies the computational workflows GigaScience values. SCSEQ’s focus on usability and utility aligns with the journal’s criteria for publication.

We would like to recommend the following four reviewers who have profound attainments in this research field and are directly related to our work for your reference:

1. Song Feng (song.feng@pnnl.gov)
Pacific Northwest National Laboratory (DOE)

2. Shuao Wang (shuaowang@suda.edu.cn)
School for Radiological & Interdisciplinary sciences, Soochow University

3. Per Eklund (perek@ifm.liu.sse)
Department of Physics, Linkoping University

4. Jinlong Zhu (jlzhu04@physics.unlv.edu)

High Pressure Science and Engineering Center and Department of Physics and Astronomy, University of Nevada

Neither the manuscript nor the underlying study violates any ethical guidelines; all data are from public repositories. The authors declare no competing interests.

Thank you for considering our work for publication.

Sincerely,

Shiyu Du, Ph.D. (Email:dushiyu@nimte.ac.cn)
School of Materials Science and Engineering,
China University of Petroleum (East China), Qingdao,
Shandong 266580, P.R. China.
School of Computer Science and Technology,
China University of Petroleum (East China), Qingdao,
Shandong 266580, P.R. China.

Jian He, Ph.D. (Email: jih003@sjtu.edu.cn)
Director of Genomics Core, Center for Single-Cell Omics
Associate Professor, State Key Laboratory of Systems Medicine for Cancer
Shanghai Jiao Tong University School of Medicine
Shanghai, PR China
