# Supplementary material for: SCSEQ: A web tool for analyzing single-cell RNA-seq data
Source: Gigascience. 2026 May 5;15:giag029. doi: 10.1093/gigascience/giag029 (PMC13142160; doi:10.1093/gigascience/giag029)
Supplement: giag029_GIGA-D-25-00531_revision_1 [file giag029_giga-d-25-00531_revision_1.pdf]

GigaScience

SCSEQ: A web tool for analyzing single-cell RNA-seq data
--Manuscript Draft--

**Manuscript Number:**	GIGA-D-25-00531R1
**Full Title:**	SCSEQ: A web tool for analyzing single-cell RNA-seq data
**Article Type:**	Research
**Funding Information:**	National Key Research and Development Program of China (2022YFD2101503) — Prof. Jian He
**Abstract:**	Single-cell RNA sequencing has emerged as a powerful approach to reveal cellular heterogeneity within biological systems. With the continuous advancement of high-throughput sequencing technologies, studies are generating vast amounts of complex data,posing a significant challenge for researchers in effective data processing and analysis. To address this issue, we developed SCSEQ,an interactive web-based bioinformatics analysis platform. This platform enables even users without programming expertise to conveniently process and analyze sequencing data. SCSEQ provides a comprehensive workflow encompassing: data preprocessing,normalization, clustering, dimension reduction, differential expression analysis, cell type identification and downstream analyses. The downstream analysis tasks include gene enrichment analysis, transcription factor analysis, cell-cell communication analysis,copy number variation detection, trajectory inference, and pan-cancer analysis. SCSEQ facilitates information transfer between different workflows, accepts various input formats, and generates graphical and tabular outputs. As a user-friendly platform, we enhance user experience through detailed parameter settings and dynamic interactions. This enables users to precisely regulate research processes and customize result figures. Additionally, we provide comprehensive user manuals to assist with parameter configuration and workflow execution. SCSEQ provides an intuitive and convenient solution for single-cell transcriptome sequencing data analysis. Our platform has successfully completed full-process analyses on real-world data with reliable results,demonstrating its applicability in practical scenarios. The platform is available at https://scseq.com.cn.
**Corresponding Author:**	Jian He<br>Shanghai Jiao Tong University School of Medicine<br>Shanghai, CHINA
**Corresponding Author Secondary Information:**	
**Corresponding Author's Institution:**	Shanghai Jiao Tong University School of Medicine
**Corresponding Author's Secondary Institution:**	
**First Author:**	Shiyu Du
**First Author Secondary Information:**	
**Order of Authors:**	Shiyu Du
	Pengcheng Sun
	Li Shen
	Jian He
**Order of Authors Secondary Information:**	
**Response to Reviewers:**	# Responds to the reviewer’s comments:<br><br>Reviewer #1:<br><br>1. Response to comment: Lack of documentation and tutorial resources<br>Since the platform is intended for non-specialists, accessible documentation is

Powered by Editorial Manager® and ProduXion Manager® from Aries Systems Corporation

essential. The manuscript does not show how data should be prepared, how tasks are executed, how parameters affect outputs, or how results are interpreted. Please provide a step-by-step tutorial or link to documentation, this would make the platform substantially more usable.
Response:
We thank the reviewer for highlighting the importance of documentation for non-specialist users. We agree that clear and accessible guidance is essential for a web-based analysis platform. In the revised manuscript, we have added a dedicated tutorial that provides a step-by-step walkthrough of a typical analysis workflow, covering data preparation, task execution, parameter configuration, and basic interpretation of results. A permanent link to this documentation has been added to the Methods section describing the platform implementation. To further improve usability, each major operation step in the platform interface is accompanied by a contextual help button, which directs users to the corresponding section of the tutorial. These revisions are intended to make the platform easier to follow for first-time users without requiring prior computational expertise.

2. Response to comment: Lack of Single-Cell Transcription Factor Analysis Module
Transcription factors participate in regulating genomic DNA accessibility, recruiting RNA polymerase for transcription, and assembling cofactors to modulate specific transcription stages during biological processes. They also govern immune responses and developmental processes in organisms. Therefore, integrating a single-cell transcription factor analysis module into cloud platforms is essential—such as one based on SCENIC.
SCENIC: single-cell regulatory network inference and clustering. https://doi.org/10.1038/nmeth.4463
Response:
We thank the reviewer for emphasizing the importance of incorporating transcription factor analysis into single-cell analysis platforms. We fully agree that transcription factors play a central role in regulating chromatin accessibility, coordinating transcriptional programs, and shaping immune and developmental processes. In response to this suggestion, we have integrated a dedicated transcription factor analysis module into SCSEQ based on the SCENIC framework. This module enables the inference of gene regulatory networks and the assessment of transcription factor activity at single-cell resolution. In the revised manuscript, we have added a description of this functionality in both the Downstream Analyses section and the Data Analysis subsection to clarify its implementation and practical usage within the workflow. We have also included the corresponding reference to SCENIC to appropriately acknowledge the methodological foundation. We believe that this addition further strengthens its downstream analytical capabilities.

3. Response to comment: Validation limited to PBMC data
The evaluation is based solely on a 10x PBMC dataset, which is a minimal and well-behaved example. It does not demonstrate performance under realistic biological scenarios (tumors, multi-sample studies, or disease contexts). I suggest adding at least one dataset involving tumor heterogeneity, multi-sample integration, or disease states to show that the platform can handle more complex settings.
Response :
We thank the reviewer for this valuable suggestion. We agree that validation using only a PBMC dataset is insufficient to demonstrate the applicability of the platform in real-world research scenarios.
In the revised manuscript, we have added an additional analysis based on a real-world liver cancer single-cell dataset containing approximately 80,000 cells. This dataset originates from another study conducted by our research group and represents a biologically complex tumor microenvironment with substantial cellular heterogeneity. The analysis was performed strictly following the standard SCSEQ workflow, and key downstream results are presented to illustrate the platform's performance in a realistic disease context.
For cell annotation, we applied the CellTypist reference model “Healthy_Human_Liver”, which enabled the identification of major liver and immune cell populations. Marker genes for each annotated cell type were calculated and visualized using dot plots, allowing intuitive inspection of cell-type-specific expression patterns. (Related images can be found in the file "Response to Reviewer")

Powered by Editorial Manager® and ProduXion Manager® from Aries Systems Corporation

Figure 1. Cell annotation results with the CellTypist reference model "Healthy_Human_Liver"

Figure 2. Visualization of marker gene expression

To demonstrate downstream functional analysis capability, we selected B cells and performed Gene Enrichment Analysis, presenting the top enriched biological processes.

Figure 3. Gene enrichment analysis of B cells showing the top enriched biological processes.

We further conducted cell–cell communication analysis using CellChat, and visualized the interaction network as circular plots, illustrating the communication landscape among major cell populations.

Figure 4. Cell–cell communication network inferred by CellChat and visualized as circular interaction plots among major cell populations.

To assess genomic instability in tumor-related populations, Copy Number Variation (CNV) analysis was performed using InferCNV. Endothelial_cells were used as the reference population, and CNV patterns were evaluated in Hepatocytes and Macrophages, revealing distinct large-scale chromosomal alterations consistent with tumor-associated changes.

Figure 5. Copy number variation analysis using InferCNV with Endothelial_cells as the reference and CNV patterns evaluated in Hepatocytes and Macrophages.

We also performed pseudotime analysis to investigate potential cellular state transitions within the tumor microenvironment. Three representative cell populations (T_cells, B_cells, and Endothelial_cells) were selected, and their developmental trajectories were reconstructed to illustrate dynamic changes along inferred biological progression.

Figure 6. Pseudotime trajectory analysis of T_cells, B_cells, and Endothelial_cells illustrating dynamic cellular state transitions.

In addition, to demonstrate the platform's clinical analysis capability, we conducted pan-cancer analysis using the gene KRT18. Results include differential expression between tumor and normal tissues across cancer types, correlations with CpG methylation and immune scores, and survival associations (OS, DSS, DFI, and PFI), highlighting the clinical relevance of the analysis.
Together, these results demonstrate that SCSEQ can effectively handle large-scale datasets, tumor heterogeneity, and complex multi-level analyses, supporting its practical utility in disease-oriented single-cell studies.

Figure 7. Pan-cancer analysis of KRT18 across TCGA cohorts showing expression differences, methylation and immune correlations, and survival associations.

4. Response to comment: Data security and multi-user isolation
As SCSEQ is cloud-based, users will reasonably ask how uploaded data are stored, isolated, and protected. The manuscript does not describe project isolation, storage policies or what metadata persist after task deletion. Clarification of these aspects would improve confidence in the platform and support broader adoption.
Response:
We thank the reviewer for highlighting this important point. In the revised manuscript, we have expanded the description of the system architecture to clarify data security and multi-user isolation mechanisms. Specifically, in the Implementations section describing the system architecture, we now state that each user project is assigned an independent workspace, with strict separation enforced at both the file system and database levels through user and project identifiers. Uploaded data are accessible only

within the corresponding project scope, and intermediate files are not shared across projects.
In addition, description of the Data Layer has been updated to explain how user_id and project_id are used jointly to constrain database queries and enforce separation between users and projects. We have also clarified the data storage and deletion policy in the file system management description, specifying that deleting a task removes its associated result files, while deleting a project triggers removal of all files under the corresponding project directory. Together, these revisions clarify how uploaded data are stored, isolated, and removed, and are intended to improve user confidence in the platform.

5. Response to comment: Provide the link or repository for the RAG reference dataset.
Response:
We have added explicit descriptions and corresponding links for the reference datasets used in the RAG module in the revised manuscript (Methods, Cell type identification section), and these resources are now cited to improve transparency and reproducibility.

6. Response to comment: Fix inconsistencies between the citation numbering and the order in which they appear in the text.
Response:
We have reviewed and revised the manuscript to ensure that all references are cited in the correct numerical order.

7. Response to comment: Figure 4 should clearly specify which gene was used to generate the futureplot and violin plot.
Response:
The gene used to generate the FeaturePlot and violin plot in Figure 4 has now been explicitly specified. In the revised manuscript, we state that CD79A was selected, and we clarify in the main text that the same gene was used for both visualizations to ensure consistency.

8. Response to comment: State which LLM model was used and basic deployment configuration.
Response:
The large language model used in the annotation module is now explicitly specified in the manuscript. We used Qwen-Max (Alibaba Tongyi Qianwen series) as the underlying LLM. The model is accessed through the official cloud 1and is used to generate candidate cell-type labels and supporting explanations based on the marker genes and tissue information provided after the RAG retrieval step. Basic implementation details have been added to improve transparency and reproducibility (Methods, Cell type identification).
To minimize data exposure, only the top marker genes and tissue information are sent to the external LLM service during the annotation step. No raw expression matrices, cell-level data, or user-uploaded datasets are transmitted. All primary data processing and analysis are performed locally within the SCSEQ environment.

9. Response to comment: Expand "RAG" on first use as "Retrieval-Augmented Generation".
Response:
The term “Retrieval-Augmented Generation (RAG)” is now defined at its first occurrence in the Introduction.

10. Response to comment: Do not capitalize "User" unless it is defined as a proper entity.
Response:
The inappropriate capitalization of “User” has been corrected at two locations, specifically in the Key Points section and in the Results and Discussion – Data Analysis subsection.

11. Response to comment: Correct the grammar in "posing significant challenge".
Response:
The phrase “posing significant challenge” has been corrected to “posing a significant

challenge" in the Abstract section.

Reviewer #2:
1. Response to comment: The most pressing issue is the availability of the web tool. The manuscript lists the platform URL as https://ape-closing-tightly.ngrok-free.app. This is an ngrok tunnel, typically used for local development and debugging. It is not a persistent, production-grade domain. Such a link relies on the author's local machine being turned on and the tunnel being active. For a web tool publication, a stable, persistent server (institutional or cloud-hosted) with a proper domain is a strict requirement. If the authors intend for users to install this locally, the "web tool" framing is slightly misleading, and the focus should shift to the Docker/local deployment ease. However, the manuscript frames it heavily as a "web-based bioinformatics analysis platform".
Response:
We thank the reviewer for raising this important concern regarding the stability and persistence of the web platform. We fully agree that a temporary ngrok tunnel is not suitable for a production-level web tool publication. In response, we have migrated the system from the temporary development tunnel to a permanently deployed cloud server with a dedicated domain.
The platform is now publicly accessible via a stable HTTPS domain: https://scseq.com.cn. The service is hosted on a persistent cloud server and configured with a valid SSL certificate, ensuring secure encrypted communication (HTTPS) and long-term availability. The domain is officially registered and maintained. The manuscript has been updated accordingly to reflect the new permanent URL.

2. Response to comment: Figure 3 (Task Information Dialog) clearly displays User Interface (UI) elements in Chinese (e.g., "任务信息" for Task Information, "运行状态" for Status, "提交时间" for Submission Time, "操作" for Operation). As this is a submission to an international English-language journal, the software interface shown in the manuscript must be in English. If the tool supports multi-language localization, this should be explicitly stated, but the default figures should be in English to ensure readability for the reviewers and readership.
Response:
We thank the reviewer for pointing out this issue. In the revised manuscript, Figure 3 has been replaced with an English-language interface to ensure readability for an international audience. In addition, a language selection button has been added to the top-right corner of the platform interface, allowing users to switch between Chinese and English, with English set as the default language. This functionality has also been clarified in the manuscript to indicate that SCSEQ supports bilingual localization while maintaining English as the primary interface.

3. Response to comment: The authors claim a "Pan-cancer analysis" feature that distinguishes SCSEQ from competitors. However, the description suggests this is merely a lookup tool for The Cancer Genome Atlas (TCGA) bulk data, rather than an analysis of the user's uploaded single-cell data. Figure 5E shows boxplots of CD8A expression in TCGA samples. Does the tool actually project the user's single-cell clusters onto TCGA bulk data (e.g., deconvolution or similarity scoring)? If it simply allows a user to "search" a gene name and see static TCGA plots, it is misleading to list this as a downstream analysis of the scRNA-seq pipeline. This needs clarification.
Response:
We thank the reviewer for raising this important point and agree that the scope of the "Pan-cancer analysis" module requires clearer explanation.
SCSEQ does not project user-uploaded single-cell clusters directly onto The Cancer Genome Atlas (TCGA) bulk RNA-seq data through deconvolution or similarity scoring. Instead, the Pan-cancer module is designed as an integrated translational analysis component that connects genes identified from the user's single-cell analysis to large-scale TCGA bulk cohorts for downstream validation and clinical relevance assessment. Specifically, once a user identifies genes of interest from scRNA-seq analyses, SCSEQ enables automated pan-cancer evaluation of those genes across TCGA datasets. This includes: (1) expression comparison between tumor and normal tissues, (2) correlation analysis between gene expression and CpG methylation levels, (3) correlation analysis with immune infiltration scores, and (4) univariate Cox regression across cancer types with forest plot visualization. These analyses are dynamically generated based on the

queried gene and are not static images.
In response to the reviewer’s comment, we have further expanded this module by incorporating Kaplan–Meier survival analysis in selected cancer types (e.g., ACC for CD8A). Patients are stratified into high- and low-expression groups based on the median expression level, and performs log-rank testing, providing statistical evidence of prognostic relevance. This addition strengthens the clinical relevance of the Pan-cancer module.
Therefore, the Pan-cancer module should be interpreted as a gene-level translational extension of the scRNA-seq workflow rather than as a projection of single-cell clusters onto bulk data. Its purpose is to facilitate rapid clinical validation and cross-cancer comparison of candidate genes discovered in single-cell analyses.
To avoid misunderstanding, we have revised the manuscript to (i) clarify that SCSEQ does not perform bulk–single-cell deconvolution or similarity projection, and (ii) explicitly describe all analytical steps included in the TCGA-based module, including methylation, immune correlation, Cox regression, and Kaplan–Meier survival analysis. We believe these clarifications more accurately define the functionality and scope of the Pan-cancer analysis feature.

4. Response to comment: The manuscript mentions using a "RAG-enhanced large language model" for cell annotation, utilizing PanglaoDB. However, critical technical details are missing: Which LLM? (e.g., GPT-4, Llama 3, Claude, or a custom model?). How is "semantic similarity computed in an embedding space"? Which embedding model is used? The authors explicitly state, "accuracy... cannot be guaranteed" and "results should be regarded as advisory". While honesty is good, including an experimental feature with no validation metrics (e.g., F1 score comparison against CellTypist) weakens the paper.
Response:
We thank the reviewer for this insightful comment. In the revised manuscript, we have provided additional technical details to clarify the implementation of the RAG-enhanced annotation module and its intended role.
Specifically, the large language model used in this study is Qwen-Max, accessed via an API service. For semantic retrieval, the external knowledge corpus is first segmented into text chunks and encoded using a SentenceTransformer model (text2vec-base-chinese). The resulting embeddings are stored and indexed in ChromaDB, where similarity between query and corpus entries is computed based on vector similarity in the embedding space. For each query, the top candidate records are retrieved and further re-ranked using a CrossEncoder model (mmarco-mMiniLMv2-L12-H384) to improve relevance. The highest-ranked records are then provided to the LLM as contextual references for generation.
We also clarify the intended role of this module. The RAG-based LLM component is designed as an exploratory and advisory tool to assist users in interpreting cluster identities, rather than as a primary or automated annotation method. The main analytical results in SCSEQ still rely on conventional approaches such as clustering-based marker identification and user validation. In our workflow, cells are first grouped into clusters, and the top marker genes (top 10) for each cluster, together with the tissue context, are provided to the LLM to infer a representative cell-type label at the cluster level. This strategy is conceptually similar to recently reported tools such as GPTCelltype and AICelltype, and is intended to provide interpretable suggestions based on biologically meaningful marker patterns.
Because the annotation is performed at the cluster level rather than the individual cell level, each predicted label affects a large group of cells simultaneously. As a result, performance metrics calculated at the cell level (e.g., F1 score) can show large fluctuations and may not accurately reflect the practical usefulness of the method. Moreover, the outcome is highly dependent on clustering quality, cell-type composition, and dataset characteristics. Given the auxiliary nature of this functionality and the variability across biological datasets, we do not position LLM outputs as definitive annotations, and therefore standalone benchmarking metrics against dedicated annotation tools are not included. This intended usage and limitation have now been explicitly stated in the manuscript.

5. Response to additional concern: The backend uses Flask and stores "Raw Files" which are "storage-heavy". The manuscript does not address how the server handles multiple concurrent users uploading large scRNA-seq matrices (which can be

Powered by Editorial Manager® and ProduXion Manager® from Aries Systems Corporation

	gigabytes in size). The demo uses a very small PBMC dataset (2,700 cells). To prove the tool is robust, the authors should benchmark it against a larger dataset (e.g., 50k+ cells) to demonstrate that the web server (and the R backend) does not time out or crash during clustering/integration.<br>Response:<br>We agree that demonstrating SCSEQ's scalability requires more than small PBMC datasets. In response, we performed additional testing on a larger, internal dataset containing over 80,000 cells. While the computation time is longer, the platform completes the workflow stably without timeouts or task failures, demonstrating robust performance under heavier data loads.<br>Furthermore, we have clarified the system's design for handling multiple concurrent users and tasks. SCSEQ supports asynchronous background execution for all analysis jobs, allowing multiple tasks from different users or projects to run independently. Each project has an isolated workspace with strict data separation at both user and project levels. Project-level task scheduling ensures that analyses are executed independently, preventing interference between concurrent tasks. These improvements are described in the revised Implementation section, highlighting multi-task handling, project isolation, and concurrency control.<br><br>6. Response to additional concern: In Table 1, the checkmarks for "Pan-cancer analysis" and "Copy number variation" favor SCSEQ. However, without clarification on how these are implemented (especially Pan-cancer), this comparison may be unfair to other established platforms like ezSingleCell which may have different scopes.<br>Response:<br>We appreciate the reviewer's comment regarding Table 1. To make the comparison fairer, we have clarified how SCSEQ implements features such as Pan-cancer analysis and Copy Number Variation analysis, including the TCGA-related processing steps described in our previous third response. At the same time, we acknowledge that SCSEQ currently does not include modules for spatial transcriptomics or other multi-omics analyses; our platform is focused specifically on single-cell transcriptomic data analysis. These clarifications have been added to the manuscript to ensure that readers understand the scope of SCSEQ and can interpret the comparison with other platforms, such as ezSingleCell, in the proper context.<br><br>7. Response to additional concern: Figure 4D (Marker gene identification) is quite small and dense; the dotplot text is barely legible.<br>Response:<br>We appreciate the reviewer's comment. The marker gene dotplot in Figure 4D has been resized and its layout adjusted to improve readability. Font spacing have been optimized so that both gene names and values are clearly legible, ensuring that the key information is easily interpretable.<br><br>8. Response to additional concern: "Looking forward,," (double comma).<br>Response:<br>All identified typographical issues, including the duplicated panel label in Figure 5 and the double comma in "Looking forward, ,", have been corrected.<br><br># Responds to the editor's comments:<br>Thank you for your letter and for the reviewers' comments concerning our manuscript. Those comments are all valuable for revising and improving our paper and the important guiding significance to our research. We have studied comments and advice carefully and have made corrections.<br>Following your suggestion, we have carefully reviewed and incorporated the five cited articles into the Related Work and Discussion sections of the revised manuscript. Meanwhile, in the 'marked manuscript', the revision are highlighted in yellow. We believe that the inclusion of these studies strengthens the scholarly context of our work and better aligns the manuscript with current methodological developments in the field. We appreciate the guidance, which has helped improve the overall quality and positioning of the manuscript.
**Additional Information:**	
**Question**	**Response**

Are you submitting this manuscript to a special series or article collection?	No
**Experimental design and statistics**<br><br>Full details of the experimental design and statistical methods used should be given in the Methods section, as detailed in our Minimum Standards Reporting Checklist. Information essential to interpreting the data presented should be made available in the figure legends.<br><br>Have you included all the information requested in your manuscript?	Yes
**Resources**<br><br>A description of all resources used, including antibodies, cell lines, animals and software tools, with enough information to allow them to be uniquely identified, should be included in the Methods section. Authors are strongly encouraged to cite Research Resource Identifiers (RRIDs) for antibodies, model organisms and tools, where possible.<br><br>Have you included the information requested as detailed in our Minimum Standards Reporting Checklist?	Yes
**Availability of data and materials**<br><br>All datasets and code on which the conclusions of the paper rely must be either included in your submission or deposited in publicly available repositories (where available and ethically appropriate), referencing such data using a unique identifier in the references and in the "Availability of Data and Materials" section of your manuscript.<br><br>Have you have met the above requirement as detailed in our Minimum	Yes

Standards Reporting Checklist?	
GigaScience has policies and guidelines in place for the use of generative AI-writing tools such as ChatGPT. If you have used such writing tools to assist with writing the manuscript this must be declared and cited in the text. Authors should not list AI-writing tools and other AI-assisted technologies as an author or co-author and should acknowledge that they are fully responsible for text generated or refined by AI-writing tools.<p><br><br>A summary of use (particularly in the introduction or among methods) needs to be included at the end of the paper, and the outputs should also be included as a supplementary file hosted in GigaDB or other open repositories. Please read our guidelines for more information. <p><br><br>By submitting to GigaScience, you are aware of the journal's AI-writing tools policy, and if you have declared use of such tools below, you have acknowledged this where appropriate in your manuscript and have made a summary of use and outputs available. <p><br><b>AI-assisted writing tools have been used in the preparation of this manuscript?	No

Powered by Editorial Manager® and ProduXion Manager® from Aries Systems Corporation

Main text

Click here to access/download;LaTeX - Main Document (TeX file);Manuscript.tex

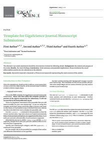

GigaScience, 2023, 1–13

doi: xx.xxxx/xxxx
Manuscript in Preparation
Paper

PAPER

SCSEQ: A web tool for analyzing single-cell RNA-seq data

Shiyu Du[1,*], Pengcheng Sun[1], Li Shen[2] and Jian He[2,*]

[1]Qingdao Institute of Software, College of Computer Science and Technology, China University of Petroleum (East China), Shandong Key Laboratory of Intelligent Oil & Gas Industrial Software, Qingdao, 266580, China and [2]State Key Laboratory of Systems Medicine for Cancer, Center for Single-Cell Omics, School of Public Health, Shanghai Jiao Tong University School of Medicine, Shanghai, 200025, China

[*]dushiyu@nimte.ac.cn; jih003@sjtu.edu.cn

Abstract

Single-cell RNA sequencing has emerged as a powerful approach to reveal cellular heterogeneity within biological systems. With the continuous advancement of high-throughput sequencing technologies, studies are generating vast amounts of complex data, posing a significant challenge for researchers in effective data processing and analysis. To address this issue, we developed SCSEQ, an interactive web-based bioinformatics analysis platform. This platform enables even users without programming expertise to conveniently process and analyze sequencing data. SCSEQ provides a comprehensive workflow encompassing: data preprocessing, normalization, clustering, dimension reduction, differential expression analysis, cell type identification and downstream analyses. The downstream analysis tasks include gene enrichment analysis, transcription factor analysis, cell-cell communication analysis, copy number variation detection, trajectory inference, and pan-cancer analysis. SCSEQ facilitates information transfer between different workflows, accepts various input formats, and generates graphical and tabular outputs. As a user-friendly platform, we enhance user experience through detailed parameter settings and dynamic interactions. This enables users to precisely regulate research processes and customize result figures. Additionally, we provide comprehensive user manuals to assist with parameter configuration and workflow execution. SCSEQ provides an intuitive and convenient solution for single-cell transcriptome sequencing data analysis. Our platform has successfully completed full-process analyses on real-world data with reliable results, demonstrating its applicability in practical scenarios. The platform is available at https://scseq.com.cn/.

Key words: Single-cell RNA sequencing; Data analysis platform; Web-based tool; Machine learning

Introduction

Single-cell sequencing technology, as a major breakthrough in modern life sciences, enables high-throughput sequencing analysis of genomes, transcriptomes, and epigenomes at the individual cell level. This technology goes beyond traditional bulk sequencing by effectively uncovering cellular heterogeneity and precisely delineating gene expression profiles. It provides novel insights and tools for advancing precision medicine and personalized therapy [1]. It can reveal gene expression profiles at single-cell resolution, thereby identifying cell types, states, and intercellular interactions, and has enabled the construction of comprehensive cell atlases across tissues and developmental stages [2]. This provides powerful tools for differential gene expression analysis and alternative splicing studies at the transcriptome level, making it a hot research topic. Currently, single-cell RNA sequencing (scRNA-seq) has become a robust technique for obtaining gene expression profiles at single-cell resolution [3], offering new perspectives for uncovering cellular heterogeneity [4].

Since the pioneering work of Tang et al. [5], which first applied high-throughput sequencing to single cells, the field has rapidly expanded with the development of diverse single-cell omics techniques, such as scWGS [6], scBS-seq [7], and scGRO-seq [8]. However, the rapid accumulation of complex and large-scale datasets poses significant challenges for effective data analysis. In 2021, Zappia and Theis reported that the scRNA tools database had cata-

Compiled on: March 12, 2026.
Draft manuscript prepared by the author.

Key Points

- SCSEQ provides a no-code pipeline for single-cell transcriptome data analysis from raw data to publication-quality visualizations.
- A highly integrated system that enables flexible fine-tuning and real-time interactive visualization guarantees reliable downstream data analysis.
- Supporting Cell Type Annotation with models trained on user datasets.
- Improving Cell Type Annotation with RAG-enhanced Large Language Models.

logued over a thousand single-cell analysis tools [9]. Among these, two computational ecosystems dominate the single-cell analysis landscape: Seurat [10] for R users and Scanpy [11] for Python users. However, their command-line interfaces and requirement for programming expertise pose significant barriers for many researchers lacking extensive coding experience. Moreover, these tools are confined to packages developed in their respective programming languages [12], which hinders the broader adoption of sequencing technologies. In contrast, tools with intuitive graphical user interfaces can significantly facilitate data analysis for researchers and clinicians. Recently, several studies have begun to provide interactive visualization of single-cell datasets through web applications built with frameworks such as Shiny, enabling users to explore published datasets more conveniently [13]. However, these implementations are typically designed for specific datasets and lack scalable computing resources and integrated analytical workflows. As a result, they remain limited compared with cloud-based platforms that support flexible data upload, large-scale computation, and comprehensive analysis pipelines.

To address this gap, we developed SCSEQ, an integrated and user-friendly web server. It enables comprehensive analyses of single-cell transcriptome data without requiring any programming knowledge. By offering intuitive workflows, extensive parameter customization, and detailed user guidance, SCSEQ aims to make advanced single-cell transcriptome sequencing data analyses accessible to more researchers and clinicians. This platform not only facilitates routine analytical workflows but also provides extensive, specialized downstream functions. It integrates a wide array of benchmark-validated tools—including Seurat, Harmony [14], SCENIC [15], CellChat [16], InferCNV [17], Monocle [18], and CellTypist [19]—and offers an expanded suite of downstream analyses such as differential expression, gene enrichment, transcription factor analysis, cell-cell communication, copy number variation, trajectory inference, and pan-cancer analysis. This integrated and updatable design makes SCSEQ a more thorough and versatile solution for single-cell transcriptomic studies, significantly enhancing its value in the rapidly evolving fields of single-cell biology and related disciplines.

The main advantages of SCSEQ are as follows:

1) We have integrated more methods, including benchmark validated methods and state-of-the-art methods, and are able to continuously update and add more excellent methods for users to use. So we are equipped to tackle a broader spectrum of downstream analytical task.
2) We introduce an advanced cell annotation algorithm based on machine learning, which allows users to upload their own datasets, train models, and annotate cells using custom or built-in models.
3) We have explored AI tools in single-cell transcriptomics analysis, integrating existing methods and leveraging large models. A Retrieval-Augmented Generation (RAG)-enhanced large language model can be used to assist in cell-type decision-making.
4) We have designed our platform with numerous adjustable parameters. This allows users to process and analyze their data according to their specific requirements.
5) We offer diverse visualization options. Users can choose what to display and adjust parameters. Real-time adjustments and previews are supported, and visualization results can be saved locally for research or sharing.

Related Work

The computational analysis of scRNA-seq data is dominated by powerful programming frameworks such as Seurat and Scanpy, which provide comprehensive analytical pipelines. Recent efforts such as SeuratExtend further streamline single-cell workflows by integrating extended analytical capabilities within a unified framework [20]. In parallel, recent methodological advances in single-cell analysis, including deep learning–based representation approaches such as scGraph2Vec [21], have further improved the ability to capture complex gene relationships from high-dimensional data. However, their command-line interfaces and dependency on specific programming languages restrict their usability for non-specialists. In response, both academic and commercial efforts have led to the development of web-based servers with graphical user interfaces [22, 23, 24]. While platforms such as ASAP [25], ICARUS [26], and CELLAR [27] have matured in handling basic analytical tasks—including data preprocessing, quality control, and cell clustering—their capabilities in advanced downstream analyses remain limited. Particularly for advanced requirements like copy number variation analysis and pan-cancer analysis, most existing platforms offer limited support. Some platforms have begun integrating specialized functions: for instance, ASAP, ICARUS, and CELLAR support cell annotation; OmicStudio [28] incorporates gene set enrichment analysis (GSEA) [29]; SciAp [30] includes trajectory inference; and ezSingleCell [31] offers cell-cell communication analysis. It is worth noting that although these platforms have made valuable attempts in multi omics data analysis, their current analytical capabilities are still insufficient to comprehensively explore single-cell transcriptomic data. There is an urgent need to develop more thorough and professional downstream analysis solutions as a supplement.

These additions are helpful, yet their current analytical capabilities remain insufficient for comprehensive exploration of single-cell transcriptomic data, most servers still leave copy number variation analysis and pan-cancer exploration outside their scope. A single portal that marries routine steps to deep, specialised modules is still missing. SCSEQ was built to close that gap. As mentioned above, we have integrated a large number of advanced tools and can keep the tools updated continuously. These tools enable comprehensive visualization functions and also enable more complete downstream analysis. In addition, our platform provides many adjustable parameters, allowing users to modify parameters to adjust the results before analysis, and adjust visualization parameters to meet personal aesthetic preferences after analysis. For inexperienced users, we provide default parameters to simplify the operation. All parameters and corresponding results are systematically archived, enabling users to track and analyze the source and compare the results of different parameter settings to determine the best configuration. Our implementation and optimization of data processing

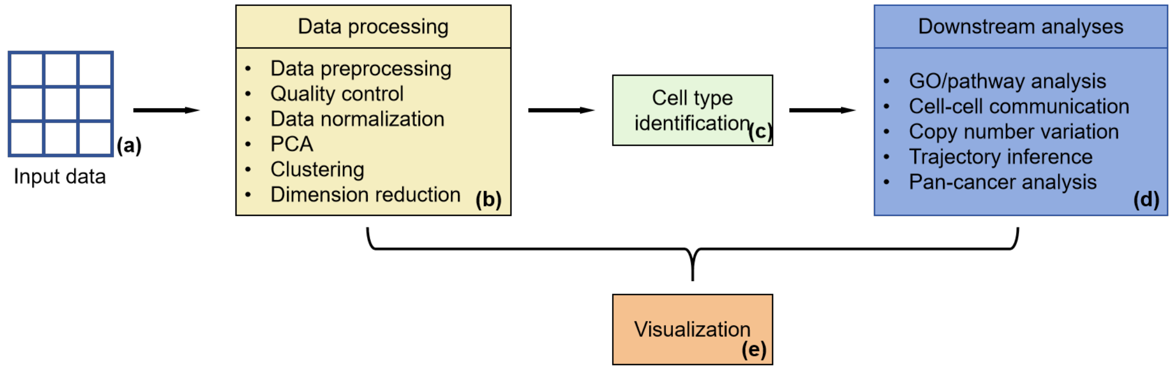

Figure 1. Analysis Process. User-submitted data goes through three key analytical stages: Data Processing, Cell Type Identification, and Downstream Analyses. Visual results from these processes are showcased on the front page.

and visualization will help researchers analyze sequencing data.

Methods

The platform's data analysis workflow is shown in Figure 1. Users start projects by uploading sequencing data. The backend then runs a basic analysis (Fig. 1b) based on the uploaded data and project details. Once the basic analysis is confirmed as accurate, the system moves on to advanced analysis. Since many follow-up tasks rely on cell annotations, we place special emphasis on the cell type identification step. SCSEQ offers multiple annotation methods, and after users confirm the annotation results, downstream analyses can proceed (Fig. 1d). Both basic and advanced analyses are controlled by user-defined parameters. All results are stored for visualization and displayed to users through the frontend interface. A tutorial for users without possess professional knowledge is available at [32]. Next, we will introduce the methods used in this platform.

Bioinformatics software tools

Basic analysis

For the basic analysis phase, we primarily employed methods from the Seurat package. Seurat is an R package tailored for scRNA-seq data analysis. It provides a comprehensive toolkit that enables researchers to extract meaningful biological insights from raw data and reveal cellular heterogeneity.

We performed quality control using Seurat by calculating four key metrics for each cell: the number of detected genes (nFeature_RNA), UMI counts (nCount_RNA), the percentage of mitochondrial genes (percent.mt), and the expression proportion of hemoglobin genes (percent.hb). Following cell filtration, we normalized the data using log-normalization. Highly variable genes were identified using the FindVariableFeatures function, followed by dimensionality reduction through principal component analysis (PCA). Based on the PCA results, we constructed a K-nearest neighbor (KNN) graph and performed cell clustering using the FindNeighbors and FindClusters functions. Finally, we visualized the cell population clusters by further reducing dimensions with either t-SNE or UMAP methods.

Cell type identification

To identify cell types and lay a foundation for downstream analyses, we offer two main annotation methods: SingleR and CellTypist. SingleR is an R package tailored for cell type annotation of scRNA-seq data. It infers cell types for unannotated single-cell data by comparing it to reference datasets. CellTypist is a Python package for annotating single-cell data, employing stochastic gradient descent to train logistic regression classifiers. Users can not only select CellTypist's built-in reference models but also upload annotated data as training sets to develop customized reference models. Relative to built-in models, user-defined models typically exhibit improved compatibility with single-cell datasets, demonstrate superior performance in specific cell populations (e.g., rare or newly discovered cell types), and, when adequate training data are available, provide more accurate annotations.

Additionally, recent advances in artificial intelligence have increasingly supported biological data interpretation and functional inference [33, 34]. A large language model (LLM) is employed as a supplementary annotation component. In the current implementation, the LLM is Qwen-Max (Alibaba Cloud) and is accessed through an API service. To minimize data exposure, only the tissue name and the top 10 marker genes for each cluster are transmitted to the LLM, while raw expression matrices and cell-level data are not shared. All primary data processing and analysis are performed locally within the SCSEQ environment. LLM returns candidate cell-type labels along with supporting rationale. Because general-purpose LLMs may have limited bioinformatics knowledge, a retrieval-augmented generation strategy is adopted. RAG is an artificial intelligence framework that integrates information retrieval and language generation [35]. By retrieving relevant information from external knowledge base, RAG can provide corresponding reference for large models and enhance the model's performance. Within the scRNA-seq workflow, the widely used single-cell transcriptome database (e.g., PanglaoDB[36]) is selected, which is often referred to for manual annotation. After data cleaning to ensure that each record contains the tissue name, marker genes, and cell-type label, the curated corpus serves as the external knowledge base. For each cluster pending annotation, relevant records are retrieved and ranked by semantic similarity computed in an embedding space. Specifically, text records are encoded using a SentenceTransformer model (text2vec-base-chinese), and the embeddings are stored and indexed in ChromaDB for vector-based similarity retrieval. The top candidate records are further re-ranked using a CrossEncoder model (mmarco-mMiniLMv2-L12-H384) and then supplied to the LLM as contextual references to support cell-type inference.

Nevertheless, the accuracy of this approach cannot be guaranteed, and the results should be regarded as advisory, with final decisions left to the user. The reliability of LLM predictions depends largely on the quality of clustering and the representativeness of the marker genes. When clusters are well defined and marker genes accurately reflect a single cell population, prediction reliability is improved; however, the outputs are intended only as a supplementary reference rather than definitive annotation results.

Downstream analyses

Before diving into other analytical tasks, we routinely carry out differential gene expression analysis using the 'FindAllMarkers' and 'FindMarkers' functions from the Seurat package. These functions systematically pinpoint genes that show statistically significant

expression differences between specific cell populations or conditions. Following this, we delve into Gene Ontology (GO) Enrichment Analysis. This powerful bioinformatics approach aids researchers in understanding the roles of genes or gene sets across biological processes, molecular functions, and cellular components.

To investigate transcriptional regulatory mechanisms, we incorporate SCENIC for transcription factor analysis. SCENIC enables the identification of regulons and the estimation of their activity at single-cell resolution. By integrating regulon activity scores into the analytical workflow, we systematically assess transcription factor activation patterns across different cell populations, providing insights into cell-type-specific regulatory programs.

To explore intercellular communication mechanisms, we leverage the CellChatDB reference database. It offers a comprehensive repository of ligand-receptor interactions and signaling pathways. This resource enables us to systematically analyze and visualize cell-cell communication networks within the biological system under investigation.

To detect genomic abnormalities, we conduct Copy Number Variation (CNV) analysis to identify changes in DNA segment copy numbers. In SCSEQ, we use InferCNV, a software package that effectively distinguishes tumor cells from normal cells based on CNV profiles.

Our analytical pipeline also includes two advanced methods: Trajectory Inference and Pan-cancer Analysis. For Trajectory Inference, we use the Monocle package to reconstruct cellular developmental pathways and transitions. For Pan-cancer Analysis, we utilize The Cancer Genome Atlas (TCGA) data to conduct cross-cancer comparative studies at the gene level. Rather than projecting single-cell clusters onto bulk data, this module enables downstream validation of user-identified genes through expression comparison, immune correlation, and survival analyses across multiple cancer types.

Application development technologies

Front end

The SCSEQ front end is built with Vue, a progressive JavaScript framework. Vue excels in responsive data binding, allowing the page to reflect data changes instantly. This real-time interactivity is ideal for visualizing analytical results and providing immediate feedback. Vue's component-based approach lets us quickly build efficient, visually appealing web applications. This boosts development speed and enhances the user experience.

Back end

The backend of SCSEQ is built with Flask, a lightweight Python web framework. Flask is simple, flexible, and highly extensible. Its streamlined design makes it easy to integrate tools for diverse scenarios and manage complex tasks, which is ideal for scRNA-seq analysis. Flask supports URL parameter parsing and static file serving, ensuring robust request handling and resource access to streamline real-time data exchange.

Database

For database management, we selected MySQL, an open-source relational database management system recognized for its high performance, reliability, and user-friendly features. Widely adopted across applications of varying scales, MySQL serves as the backbone for systematically storing information generated by SCSEQ through three dedicated tables: user table, project table, and task table.

Implementations

In this section, we introduce the overall architecture of SCSEQ, delve into the architecture and workflow. We offer a thorough explanation of each component's functionality and a comprehensive overview of the entire workflow, while also showcasing the system's user-friendly features.

Building a system that is both efficient and user-friendly is of utmost importance in our research. SCSEQ opts for a front-end–back-end separation architecture. This design pattern is widely used in web application development. It separates the front-end user interface from the back-end service logic, allowing for independent deployment and maintenance. The platform design follows principles of reproducibility and standardized data management consistent with recent guidelines for computational biological systems [37]. Additionally, the use of interfaces for communication between the front-end and back-end simplifies functional expansion and service upgrades, thereby considerably improving the system's scalability.

Figure 2 provides an overview of our application. SCSEQ primarily consists of four key components: the View Layer, Control Layer, Computation Layer, and Data Layer. The View Layer, implemented as a responsive web interface, serves as the primary interaction portal where researchers can visualize analytical results and configure parameters through intuitive graphical components. The User Management module provides centralized administration of authentication details and account preferences, while the Project Management module shows all projects under the user account. Our platform architecture supports concurrent multi-project workflows; however, each project is assigned an independent workspace and maintains strict data isolation at both the user and project levels. All project resources are logically scoped using user identifiers and project identifiers, ensuring that data access is restricted exclusively within the corresponding project context. Initial data ingestion is limited to the project initialization phase to support computational reproducibility and version control integrity. Within the Project Management interface, users can comprehensively administer existing projects while also initiating new analytical workflows by submitting requisite data files through our standardized upload protocol. After selecting their desired project, users can configure relevant parameters according to their needs and submit analysis tasks. Upon task completion, the platform will present visualization results on the interface. Users can either view these results online or download them for local storage. All chart results displayed on this platform are available for direct download and saving.

The Control Layer handles interactions between the frontend and backend, as well as some backend functions in the webserver. Its main role is to process requests and data, enabling communication and data exchange between components. This layer receives files and parameter settings from the client interface, directs the Computation Layer to perform customized data processing that meets each user's specific needs, and returns the results to the client interface. For users without programming experience, the Control Layer acts as a capable assistant. It stores uploaded data locally and passes user-defined parameters to the corresponding functions in the Computation Layer for processing and visualization. Researchers can simply interact with intuitive form fields and selection menus on the frontend interface without needing to understand the underlying technical details. After submitting tasks, users can easily monitor progress and receive visualization outputs. All analysis jobs are executed as asynchronous background tasks on the server. This design prevents the web interface from blocking during long-running computations and allows multiple tasks from different users or projects to be processed concurrently and independently. This streamlined workflow significantly reduces the technical barriers to biological data analysis. The Computation Layer contains all data processing methods and downstream analysis algorithms. This component integrates several high-quality solutions, which will be detailed in the 'Methods' section.

The Data Layer manages data storage and is split into database and file system parts. For the database, we use MySQL and set up three tables:

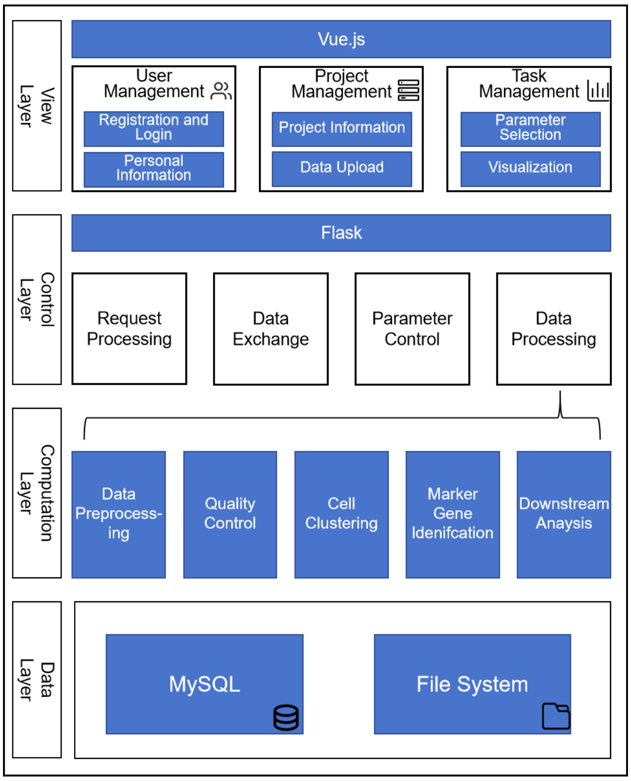

Figure 2. System Overview. SCSEQ is composed of four main components. The View Layer handles user interactions. The Control Layer receives data and parameters from the View Layer and passes them to the Computation Layer to execute algorithms or functions. The Data Layer stores all relevant data and task records.

- User Table: This stores personal user details, including login credentials.
- Project Table: This holds all project data and links to the User Table via user_id. Besides basic info like project_id, user_id, project_name, and creation_time, it also keeps key analysis data such as species studied, user notes, raw data paths, and work directories. Each project entry is uniquely associated with a single user through user_id, and all database queries are constrained by both user_id and project_id to enforce strict separation between users and projects. This setup allows users to view and manage only their own projects within the Project Management interface.
- Task Table: This records all tasks linked to projects via project_id. It includes details like task_id, task_type, parameters, result paths, submission time, and jobid for tracking. In SCSEQ, this table does three main things: lets users review all past tasks, logs parameters for each task, and notes where task results are stored. All task records are scoped to their corresponding projects, preventing cross-project access. In addition, submitted tasks are scheduled and executed independently at the project level, ensuring that multiple analysis jobs can run concurrently without interfering with each other. These features make analyses traceable and reproducible, boosting SCSEQ's usefulness.

The file system stores various files generated during platform operations. Strategies vary by file type:

- Raw Files: These are user-uploaded sequencing data, often in matrix format and storage-heavy. They're mainly used for preprocessing, after which data is stored as RDS files. Raw files are stored within project-specific directories and are not shared across projects or users. The system has been tested on internal datasets containing more than 80,000 cells; although larger datasets require longer computation time, the analysis workflow remains stable without server timeouts or task failures.
- Intermediate Files: Generated during analysis (e.g., InferCNV creates intermediate files at each step). As they're regenerated with each task, we don't specially retain them.
- Result Files: These include charts and visualization data, which take up little space. We keep all result files, naming them with task type and timestamp for easy comparison. Users can delete task records via the frontend, which also deletes corresponding result files. Deleting a project triggers removal of all associated files under the corresponding project directory.

Results and discussion

Benchmarking SCSEQ against existing platforms

SCSEQ specializes in single-cell transcriptomics analysis, completing a complete data analysis pipeline. Throughout the analytical process, we have integrated multiple excellent methods and provided numerous analysis tools. Using these methods, users can perform basic data processing as well as advanced downstream analyses. Inspired by ezSingleCell, the integrated tools and their comparisons with other similar platforms are shown in Table 1. To provide a fair comparison, we clarify that SCSEQ focuses specifically on transcriptomic analyses and does not currently include modules for spatial transcriptomics or other multi-omics analyses. In comparison,SCSEQ offers a broader range of advanced downstream analytical functionalities. For these tools, we provide default parameters while also supporting user-defined parameter inputs, ultimately obtaining high-quality visualization results.

Advantages of SCSEQ

Benefiting from the reasonable system architecture described previously, SCSEQ has numerous user-friendly and practical features:

Multi-task concurrency and flexible task scheduling: The system extends task management functionality on the project page(Fig. 3), implemented as a dialog interface. This allows users to view comprehensive task information including task type, relevant parameters, execution status, and submission time - all queried from the Task Table in the database. The operation panel allows users to review results or delete records for any task. This design improves task scheduling. Users can submit tasks, shut down their computers temporarily, and check results later. It also helps in planning follow-up analysis workflows. Additionally, the system supports multi-task concurrency. Users can run multiple projects at the same time without waiting for current tasks to finish. They can monitor all task statuses and access results through the unified task management dialog.

Comprehensive parameter configuration: SCSEQ incorporates a wide range of adjustable parameters, enabling users to precisely control the analytical process. Thanks to the well-designed task table, SCSEQ can save the parameters configured by users for each submitted task. This allows for retrospective analysis and comparative evaluation of results obtained with different parameter settings. Additionally, the system provides default parameter sets optimized for most analytical workflows, simplifying user operations. For visualization outputs, parameter controls are implemented, allowing users to customize elements such as font sizes and axis ranges, thus achieving personalized data visualization.

Interactive visualization and diverse visualization outputs: To enhance user experience and deliver richer insights, SCSEQ uses interactive visualizations with ECharts components. For example, in marker gene dotplots, users can hover over nodes to see detailed information like 'Cell Type', 'Gene Type', 'avg exp', and 'pct1' values. SCSEQ also offers various visualization types, including violin

Table 1. A comparative analysis of SCSEQ and current academic web platforms for single-cell analysis tasks.

Web server	Ours	ezSingle-Cell	ICARUS	ASAP	alona	Cellar	SCiAp	NASQAR	SCTK	Asc-Seurat
Clustering and dimension reduction	✓	✓	✓	✓	✓	✓	✓	✓	✓	✓
Cell type identification	✓	✓	✓	✓	×	✓	✓	×	✓	×
GO/pathway analysis	✓	✓	✓	✓	×	✓	✓	✓	✓	✓
Cell-cell communication	✓	✓	×	×	×	×	×	×	×	×
Copy number variation	✓	×	×	×	×	×	×	×	×	×
Trajectory inference	✓	×	×	×	×	×	×	×	×	×
Pan-cancer analysis	✓	×	×	×	×	×	×	×	×	×

Note: ✓ and × denote whether the web server supports the functionality.

plots, scatter plots, bar charts, dotplots, circle plots, heatmaps, box plots, and forest plots. These options allow users to select the most intuitive representation for their analytical tasks.

Real-time updates and immediate feedback: The platform's cloud tools allow real-time updates to existing results. For example, users can choose to display the number of genes per cell population in the marker gene results, and the chart will update immediately. For cloud analyses requiring computation to produce results, users need to wait until the analysis task completes to view the visualization outcomes. Once a task is complete, the results are instantly visible on the current page. This design minimizes debugging time for users, aids in understanding how parameters affect outcomes, helps identify more suitable parameters, and ultimately leads to better results.

Data Analysis

To showcase SCSEQ's capabilities, we analyzed a dataset of 2,700 peripheral blood mononuclear cells (PBMCs)[38] and present the results (Figs. 4,5,6,7).

After uploading the data, we conducted basic analysis using default parameters from Seurat's official documentation. SCSEQ initially displayed the overall data distribution and provided quality control metrics such as nFeature_RNA, nCount_RNA, and percent.mt (percent.hb was optional and not used in this case). We used a violin chart to visualize the data before and after cell filtering (Fig. 4A). The subsequent steps involved log-normalization, identification of highly variable genes, data scaling, and PCA. Based on the PCA results, we constructed a KNN graph and performed clustering at a resolution of 0.5 (default), resulting in 9 distinct clusters (Fig. 4B).

In the process of cell annotation, we used celltype's "Immune_All_Low" reference set. This identified 12 cell populations, including B cells, CD16+ NK cells, Classical monocytes, DCs, MAIT cells, Megakaryocytes/platelets, Non-classical monocytes, Regulatory T cells, Tcm/Naive cytotoxic T cells, Tcm/Naive helper T cells, Tem/Effector helper T cells, and Tem/Trm cytotoxic T cells. We also show the proportion of each cell type in the total cell population (Fig. 4C). To aid downstream analysis, we calculated marker genes for each annotated cluster and present them in tables and dot plots (Fig. 4D). Additionally, users can select specific genes to examine their expression patterns across all cell populations. In this study, CD79A was selected as an example, and the same gene was used to generate both the feature plot and the violin plot shown in Fig. 4D.

After basic analysis, advanced analysis can be performed. We first performed GO Enrichment Analysis. We selected biological processes related to B cells and visualized the top 10 terms by 'Count' value using bar plots (Fig. 5A).

For cell-cell communication analysis, we employed the CellChatDB database to examine ligand-receptor pairs (Fig. 5B). As shown in the left panel of Fig. 5B, all cell types are selectable, so users can also selectively examine interaction results between specific cell types of interest and other cells. For instance, in the right panel of Figure 5B, we selected B cells for visualization.

Copy number variation analysis was conducted using InferCNV, with Tcm/Naive_cytotoxic_T_cells as reference set alongside DC and Megakaryocytes/platelets populations (Fig. 5C).For trajectory inference analysis, we selected the three most abundant cell populations: B_cells, Tcm/Naive_helper_T_cells, and Classical_monocytes. The platform also supports examining gene expression dynamics along trajectories (Fig. 5D). We specifically analyzed the temporal expression patterns of ISG15, RPL22, RPL11, SH3BGRL3, CD52, and IFI6 genes.

We next performed transcription factor analysis to investigate transcription factor regulatory activity. Regulon activity scores were calculated and integrated for downstream analysis. A dot plot summarizes regulon activity across annotated cell types (Fig. 6A), and a heatmap presents average activity patterns among cell populations (Fig. 6B). We then focused on the IRF7 regulon, visualizing its spatial distribution on the UMAP embedding (Fig. 6C). A violin plot further illustrates the distribution of IRF7 regulon activity across different cell types (Fig. 6D).

Pan-cancer analysis of CD8A gene was performed using TCGA tools, with results presented through box plots, heatmaps, and forest plots (Fig. 7). The boxplot(Fig. 7A) shows CD8A expression differences between tumor (red) and normal tissues (green) across multiple cancer types. Two heatmaps highlight: (1) links between CD8A expression and CpG methylation(Fig. 7B), indicating epigenetic regulation of T cell infiltration and treatment potential; and (2) relationships between CD8A expression and macrophage immune scores across cancer types(Fig. 7C), shedding light on immune cell interactions and tumor diversity. A forest plot(Fig. 7D) presents survival analysis results for target gene expression data across different cancer types in TCGA. To further evaluate the prognostic value of CD8A, Kaplan–Meier survival analysis was performed in Adrenocortical Carcinoma (ACC), with patients stratified into high- and low-expression groups based on the median CD8A level (Fig. 7E). The Kaplan–Meier curves illustrate differences in overall survival between the two groups, and statistical significance was assessed using the log-rank test, providing direct evidence of the clinical relevance of CD8A expression in ACC.

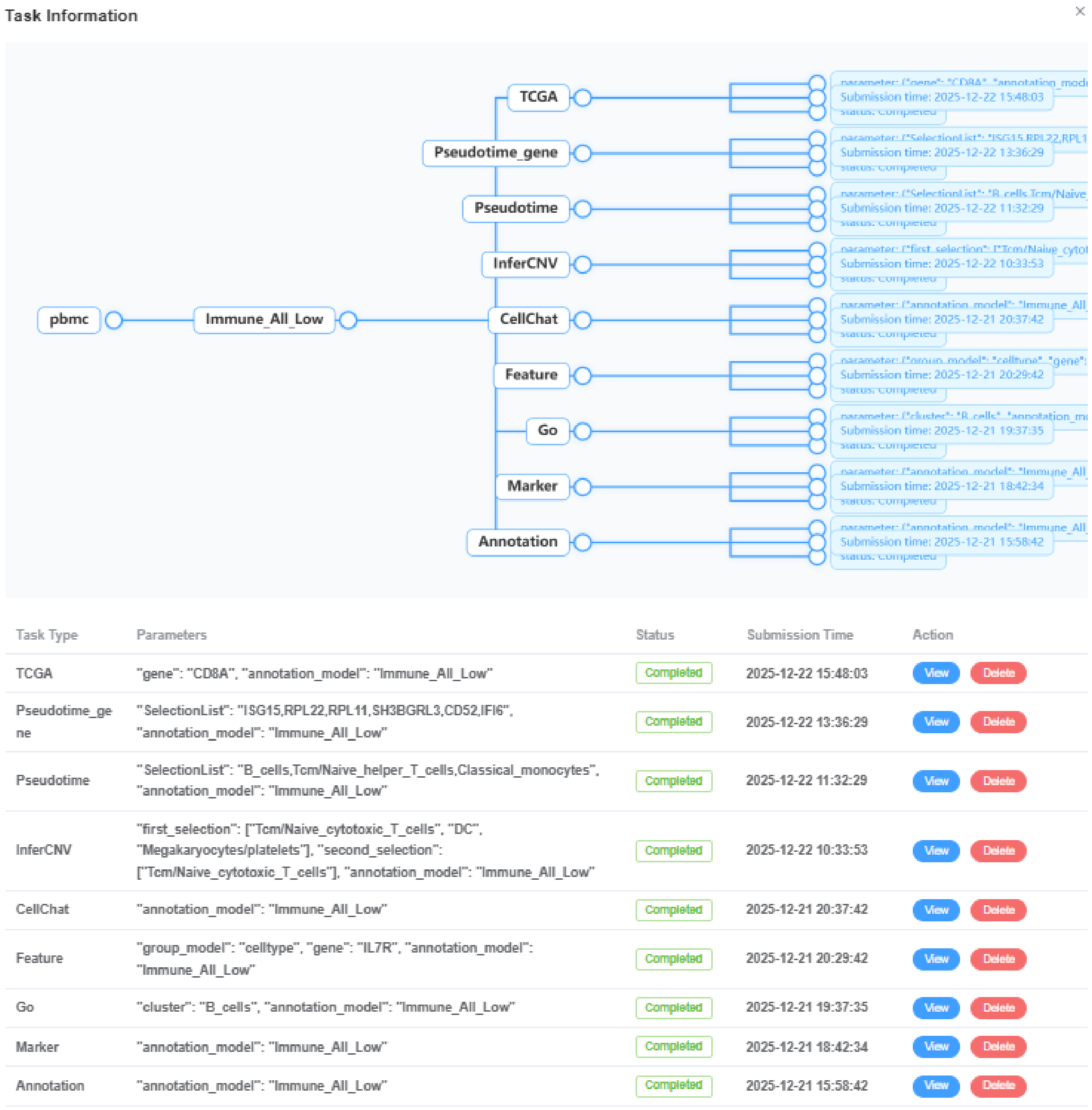

Task Type	Parameters	Status	Submission Time	Action
TCGA	"gene": "CD8A", "annotation_model": "Immune_All_Low"	Completed	2025-12-22 15:48:03	View Delete
Pseudotime_gene	"SelectionList": "ISG15,RPL22,RPL11,SH3BGRL3,CD52,IFI6", "annotation_model": "Immune_All_Low"	Completed	2025-12-22 13:36:29	View Delete
Pseudotime	"SelectionList": "B_cells,Tcm/Naive_helper_T_cells,Classical_monocytes", "annotation_model": "Immune_All_Low"	Completed	2025-12-22 11:32:29	View Delete
InferCNV	"first_selection": ["Tcm/Naive_cytotoxic_T_cells", "DC", "Megakaryocytes/platelets"], "second_selection": ["Tcm/Naive_cytotoxic_T_cells"], "annotation_model": "Immune_All_Low"	Completed	2025-12-22 10:33:53	View Delete
CellChat	"annotation_model": "Immune_All_Low"	Completed	2025-12-21 20:37:42	View Delete
Feature	"group_model": "celltype", "gene": "IL7R", "annotation_model": "Immune_All_Low"	Completed	2025-12-21 20:29:42	View Delete
Go	"cluster": "B_cells", "annotation_model": "Immune_All_Low"	Completed	2025-12-21 19:37:35	View Delete
Marker	"annotation_model": "Immune_All_Low"	Completed	2025-12-21 18:42:34	View Delete
Annotation	"annotation_model": "Immune_All_Low"	Completed	2025-12-21 15:58:42	View Delete

Figure 3. Task Information Dialog. This page displays task information under the current project and build a tree view based on the annotation method.

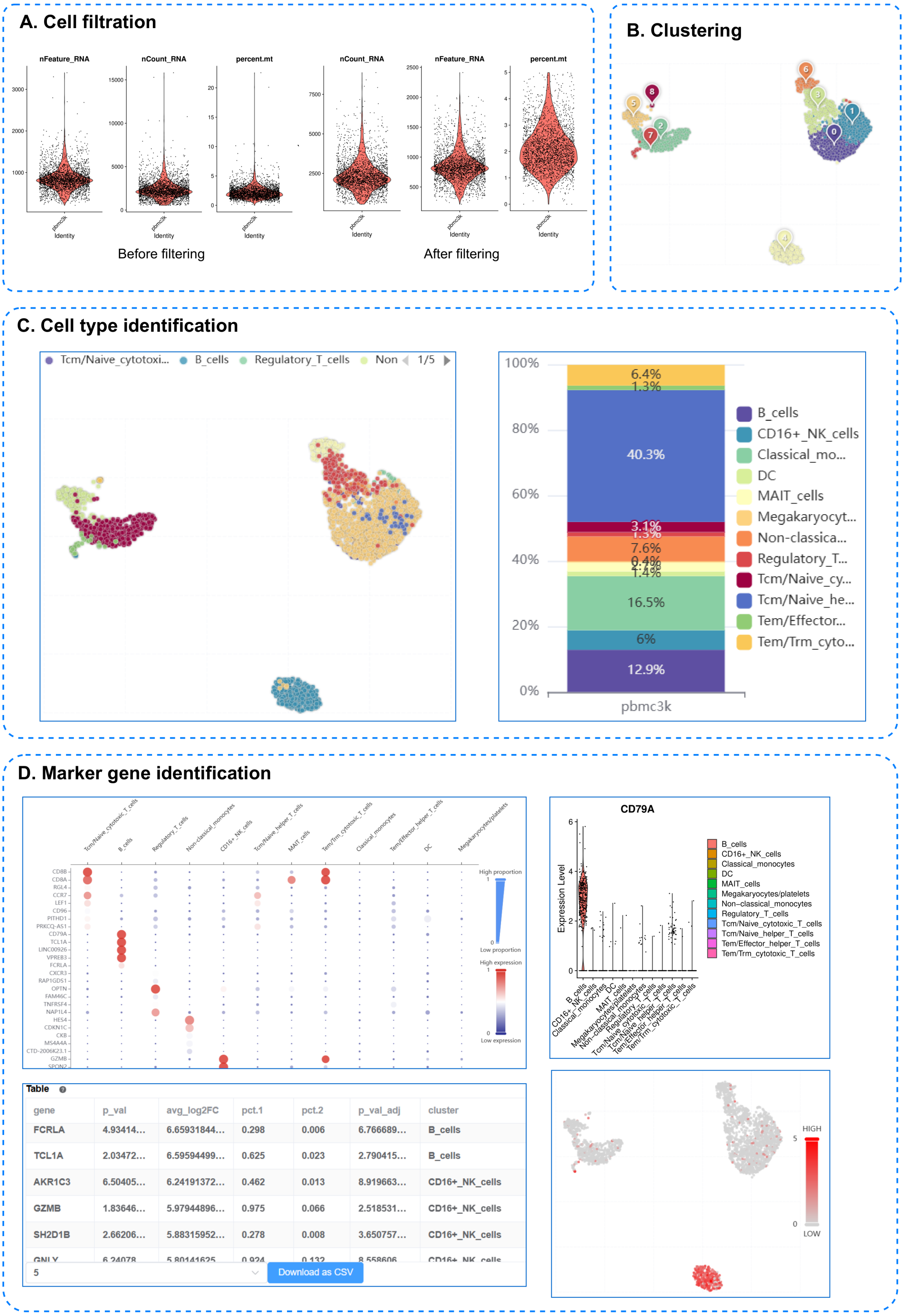

Figure 4. Basic analysis and cell annotation. **(A)** Comparison before and after cell filtration; **(B)** Visualization of clustering results; **(C)** CellTypist annotation results and cell proportion plots; **(D)** Marker gene tables and dotplot visualizations. Distribution of individual genes across all cell populations.

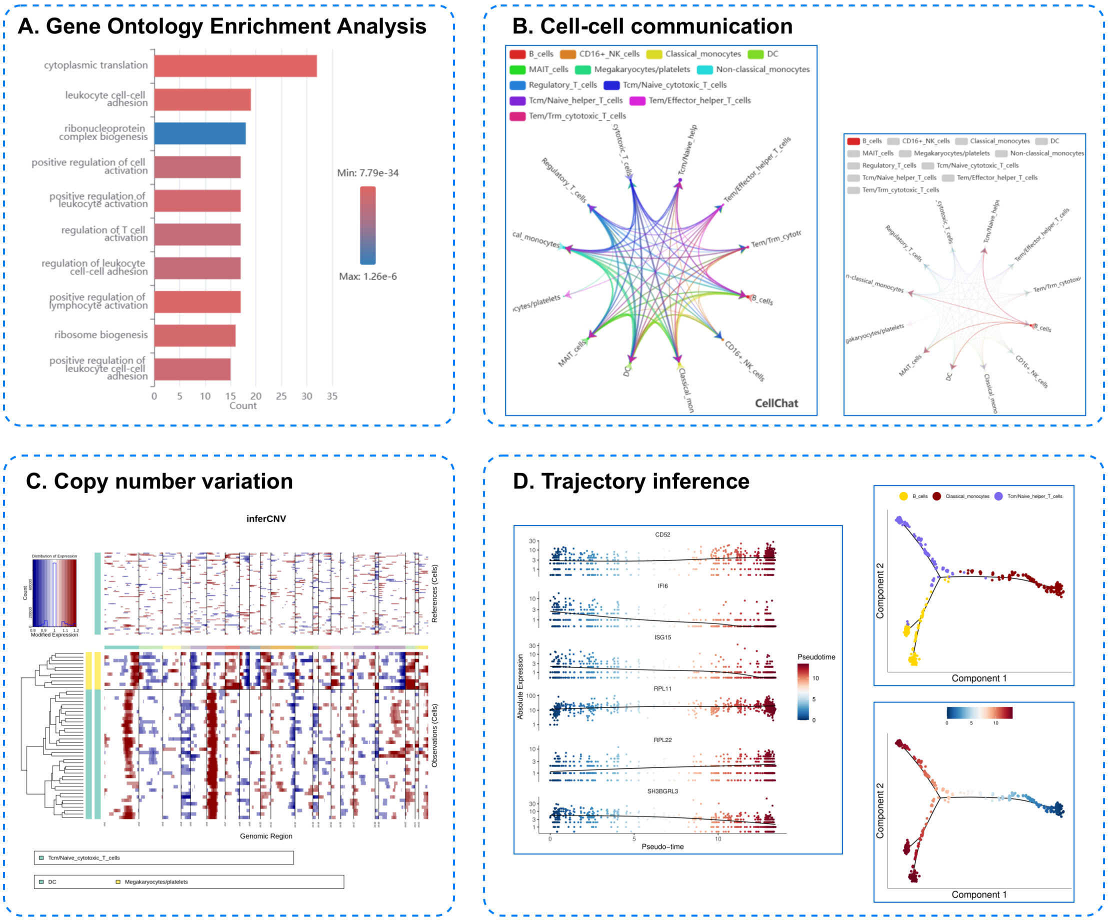

Figure 5. Advanced analysis. (A) GO enrichment results of B cells; **(B)** Cell-cell communication analysis using CellChatDB; **(C)** Copy number variation analysis using InferCNV; **(D)** Cell and gene expression dynamics along trajectories.

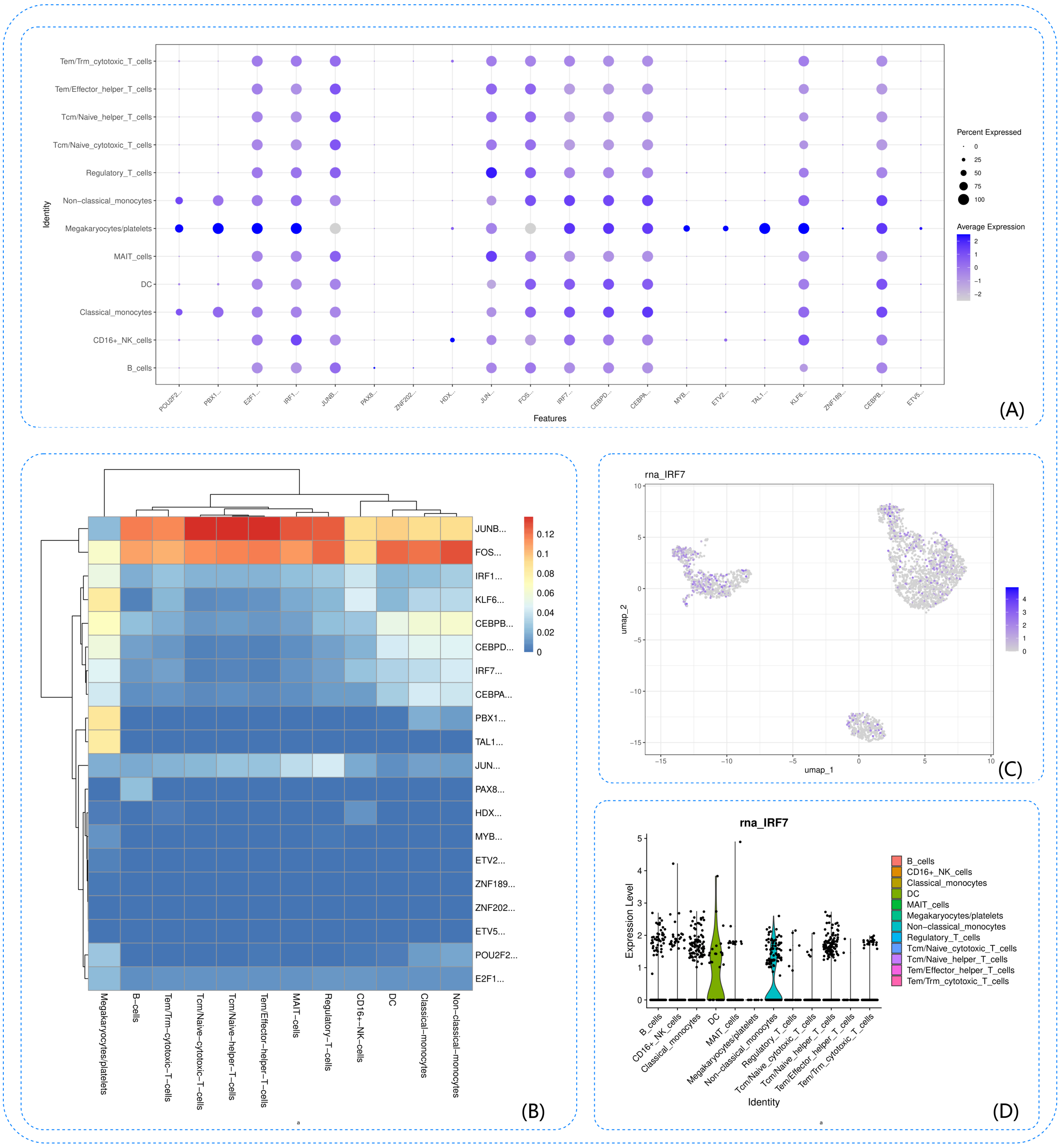

Figure 6. Transcription factor analysis. (A) Dot plot of regulon activity across annotated cell types; **(B)** Heatmap showing average regulon activity among cell populations; **(C)** UMAP visualization of IRF7 regulon activity; **(D)** Violin plot of IRF7 regulon activity across different cell types.

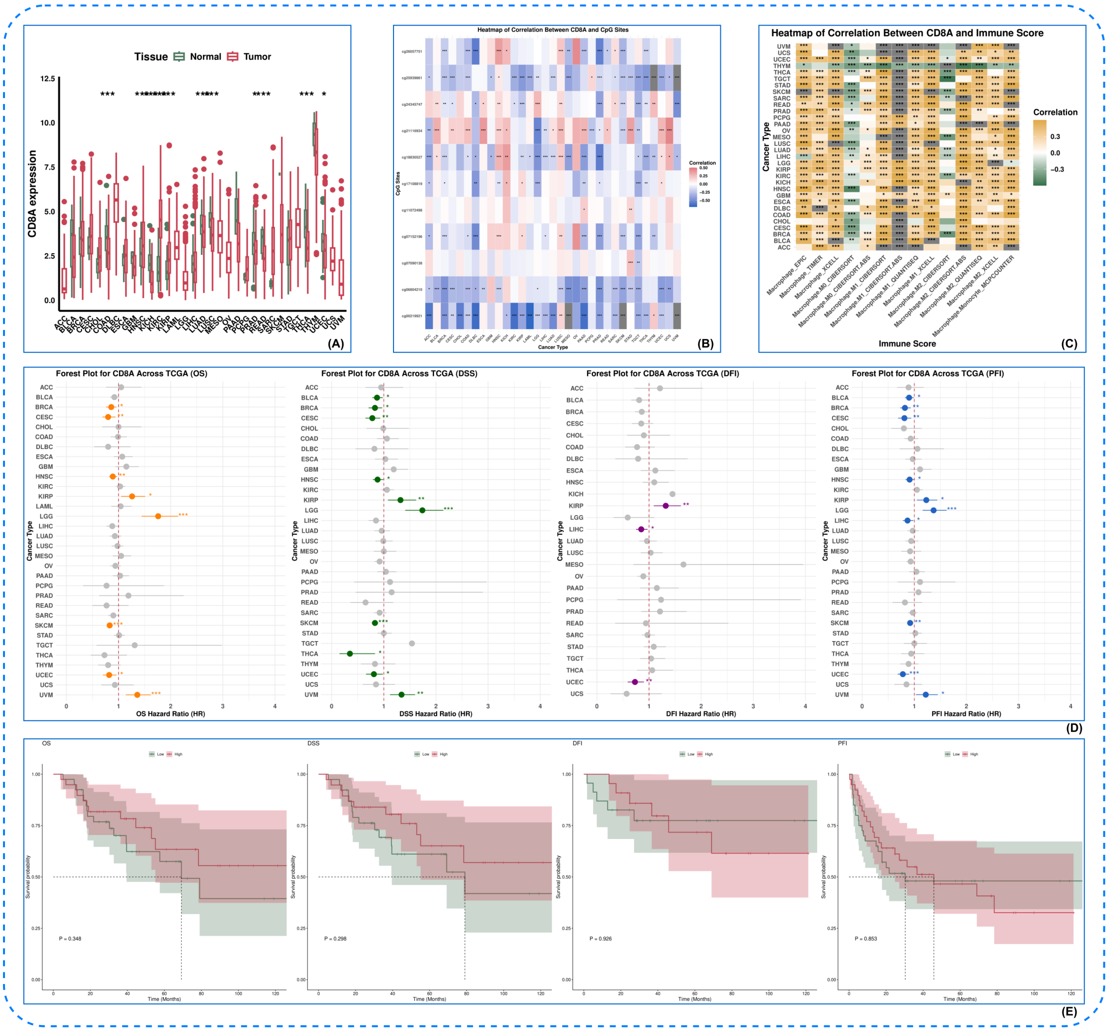

Figure 7. Transcription factor analysis. **(A)** Pan cancer box plot; **(B)** CpG methylation heatmap; **(C)** Gene–Immune Infiltration Correlation Heatmap; **(D)** Pan-cancer univariate Cox regression forest plot; **(E)** Pan-cancer Kaplan–Meier survival analysis.

Conclusion

SCSEQ consolidates an unusually broad slice of single-cell science into one coherent web workspace. Cell-type labelling, gene enrichment analysis, transcription factor analysis, cell-cell communication analysis, copy number variation detection, trajectory inference, and pan-cancer analysis and high-resolution visualisation are no longer scattered across different packages; they are accessible through a single menu, callable with a few clicks and parameter sliders. Behind the interface sits a containerised backend that wraps rigorously benchmarked tools—Seurat, Harmony, CellChat, SCENIC, InferCNV, Monocle, and CellTypist —so that every step inherits the statistical robustness of the original code while gaining the reproducibility of version-tracked workflows. Users can therefore move from raw count matrix to publication-grade figures without installing interpreters, managing dependencies or editing scripts. The platform's parameter logging and real time visualization further transform exploratory analysis into an intuitive feedback loop: adjust parameters, observe results, and export satisfactory images.

Looking forward, the platform's impact on bioinformatics will continue to grow, and we are extending the architecture along two complementary axes. First, our platform currently focuses solely on single-cell RNA sequencing and does not support Spatial Transcriptomics or single-cell Multiomics (scMultiomics), representing one of our key directions for future development. In addition, the artificial intelligence approaches applied in our cell type identification module have demonstrated excellent performance, which inspires us to incorporate more artificial intelligence analysis tools in future updates to further enhance the performance of SCSEQ and contribute to the rapid development of single-cell biology and related disciplines.

Availability of source code and requirements

- Project name: SCSEQ
- Project home page: https://github.com/knight-spc/SCSEQ
- Operating system(s): Platform independent
- Programming language: Python, Vue
- Other requirements: Anaconda (Recommended)
- License: GPL-3.0 license

Data Availability

The dataset used to illustrate SCSEQ's performance consists of 2,700 Peripheral Blood Mononuclear Cells (PBMC) sequenced on the Illumina NextSeq 500. Raw data and the processed count matrix are available from the 10x Genomics [38].

Declaration

Declaration of competing interest

We declare that we have no financial and personal relationships with other people or organizations that can inappropriately influence our work.

Declaration of generative AI and AI-assisted technologies in the manuscript preparation process

During the preparation of this work the author used Kimi K1.5 in order to translate and polish the manuscript text. After using this tool, the author reviewed and edited the content as needed and take full responsibility for the content of the published article.

Acknowledgements

This work was supported by the China University of Petroleum (East China) Discipline Start-up Fund [grant number 1500-05Y23080001].This work was supported by grants from National Key Technologies Research and Development Program of China(2022YFD2101503) of J.H.

References

1. Altschuler SJ, Wu LF. Cellular heterogeneity: Do differences make a difference? Cell 2010;141(4):559–563. Doi: 10.1016/j.cell.2010.04.033.
2. Ushakumary MG, Feng S, Bandyopadhyay G, Olson H, Weitz KK, Huyck HL, et al. Cell population–resolved multiomics atlas of the developing lung. American Journal of Respiratory Cell and Molecular Biology 2025;72(5):484–495. Doi: 10.1165/rcmb.2024-0105OC.
3. Chen G, Ning B, Shi T. Single-cell RNA-seq technologies and related computational data analysis. Front Genet 2019;10:317. Doi: 10.3389/fgene.2019.00317.
4. Haque A, Engel J, Teichmann SA, Lönnberg T. A practical guide to single-cell RNA-sequencing for biomedical research and clinical applications. Genome Med 2017;9(1):75. Doi: 10.1186/s13073-017-0467-4.
5. Tang F, Barbacioru C, Wang Y, Nordman E, Lee C, Xu N, et al. mRNA-Seq whole-transcriptome analysis of a single cell. Nat Methods 2009;6(5):377–382. Doi: 10.1038/nmeth.1315.
6. Zachariadis V, Cheng H, Andrews N, et al. A highly scalable method for joint whole-genome sequencing and gene-expression profiling of single cells. Mol Cell 2020;80(3):541–553.e5. Doi: 10.1016/j.molcel.2020.08.022.
7. Smallwood SA, Lee HJ, Angermueller C, et al. Single-cell genome-wide bisulfite sequencing for assessing epigenetic heterogeneity. Nat Methods 2014;11(8):817–820. Doi: 10.1038/nmeth.3035.
8. Mahat DB, Tippens ND, Martin-Rufino JD, et al. Single-cell nascent RNA sequencing unveils coordinated global transcription. Nature 2024;631:216–223. Doi: 10.1038/s41586-024-07517-7.
9. Zappia L, Theis FJ. Over 1000 tools reveal trends in the single-cell RNA-seq analysis landscape. Genome Biol 2021;22:301. Doi: 10.1186/s13059-021-02519-4.
10. Hao Y, Stuart T, Kowalski MH, et al. Dictionary learning for integrative, multimodal and scalable single-cell analysis. Nature Biotechnology 2024;42(2):293–304. Doi: 10.1038/s41587-023-01967-y.
11. Wolf FA, Angerer P, Theis FJ. SCANPY: Large-scale single-cell gene expression data analysis. Genome Biol 2018;19:15. Doi: 10.1186/s13059-017-1382-0.
12. Luecken MD, Theis FJ. Current best practices in single-cell RNA-seq analysis: A tutorial. Mol Syst Biol 2019;15:e8746. Doi: 10.15252/msb.20188746.
13. Sun Q, Gao R, Lin Y, Zhou X, Wang T, He J. Leveraging single-cell RNA-seq for uncovering naïve B cells associated with better prognosis of hepatocellular carcinoma. MedComm 2024;5(9):e563. Doi: 10.1002/mco2.563.
14. Korsunsky I, Millard N, Fan J, et al. Fast, sensitive and accurate integration of single-cell data with Harmony. Nat Methods 2019;16:1289–1296. Doi: 10.1038/s41592-019-0619-0.
15. Aibar S, González-Blas CB, Moerman T, Imrichova H, Hulselmans G, Rambow F, et al. SCENIC: single-cell regulatory network inference and clustering. Nature Methods 2017;14(11):1083–1086. https://note.org/10.1038/nmeth.4463, 10.1038/nmeth.4463.
16. Jin S, Guerrero-Juarez CF, Zhang L, et al. Inference and analysis of cell-cell communication using CellChat. Nat Commun

2021;12:1088. Doi: 10.1038/s41467-021-21246-9.
17. Tirosh I, Izar B, Prakadan SM, et al. Dissecting the multicellular ecosystem of metastatic melanoma by single-cell RNA-seq. Science 2016;352(6282):189–196. Doi: 10.1126/science.aad0501.
18. Qiu X, Mao Q, Tang Y, et al. Reversed graph embedding resolves complex single-cell trajectories. Nat Methods 2017;14:979–982. Doi: 10.1038/nmeth.4402.
19. Domínguez Conde C, et al. Cross-tissue immune cell analysis reveals tissue-specific features in humans. Science 2022;376:eabl5197. Doi: 10.1126/science.abl5197.
20. Hua Y, Weng L, Zhao F, Rambow F. SeuratExtend: streamlining single-cell RNA-seq analysis through an integrated and intuitive framework. Gigascience 2025;14:giaf076. Doi: 10.1093/gigascience/giaf076.
21. Lin S, Jia P. scGraph2Vec: a deep generative model for gene embedding augmented by graph neural network and single-cell omics data. GigaScience 2024;13:giae108. Doi: 10.1093/gigascience/giae108.
22. LeRoy NJ, Khoroshevskyi O, O'Brien A, Stępień R, Arslan A, Sheffield NC. PEPhub: a database, web interface, and API for editing, sharing, and validating biological sample metadata. GigaScience 2024;13:giae033. Doi: 10.1093/gigascience/giae033.
23. Klamann C, Lau CJ, Ruiz-Ramírez J, Schwartz GW. TooManyCellsInteractive: A visualization tool for dynamic exploration of single-cell data. GigaScience 2024;13:giae056. Doi: 10.1093/gigascience/giae056.
24. Feng S, Calinawan A, Pugliese P, Wang P, Ceccarelli M, Petralia F, et al. Decomprolute is a benchmarking platform designed for multiomics-based tumor deconvolution. Cell reports methods 2024;4(2). Doi: 10.1016/j.crmeth.2024.100713.
25. Gardeux V, David FPA, Shajkofci A, Schwalie PC, Deplancke B. ASAP: A web-based platform for the analysis and interactive visualization of single-cell RNA-seq data. Bioinformatics 2017;33:3123–3125. Doi: 10.1093/bioinformatics/btx406.
26. Jiang A, Lehnert K, You L, Snell RG. ICARUS, an interactive web server for single cell RNA-seq analysis. Nucleic Acids Res 2022;50(W1):W427–W433. Doi: 10.1093/nar/gkac322.
27. Hasanaj E, Wang J, Sarathi A, Ding J, Bar-Joseph Z. Interactive single-cell data analysis using Cellar. Nat Commun 2022;13:1998. Doi: 10.1038/s41467-022-29744-0.
28. Lyu F, Han F, Ge C, Mao W, Chen L, Hu H, et al. OmicStudio: A composable bioinformatics cloud platform with real-time feedback that can generate high-quality graphs for publication. iMeta 2023;2:e85. Doi: 10.1002/imt2.85.
29. Subramanian A, Tamayo P, Mootha VK, et al. Gene set enrichment analysis: A knowledge-based approach for interpreting genome-wide expression profiles. Proc Natl Acad Sci USA 2005;102:15545–15550. Doi: 10.1073/pnas.0506580102.
30. Moreno P, Huang N, Manning JR, et al. User-friendly, scalable tools and workflows for single-cell RNA-seq analysis. Nat Methods 2021;18:327–328. Doi: 10.1038/s41592-021-01102-w.
31. Sethi R, Ang KS, Li M, Long Y, Ling J, Chen J. ezSingleCell: An integrated one-stop single-cell and spatial omics analysis platform for bench scientists. Nat Commun 2024;15:5600. Doi: 10.1038/s41467-024-48034-8.
32. SCSEQ Development Team, SCSEQ: A Web Tool for Analyzing Single-Cell RNA-seq Data; 2025. https://scseq.com.cn.
33. Kim DN, Yin T, Zhang T, Im AK, Cort JR, Rozum JC, et al. Artificial intelligence transforming post-translational modification research. Bioengineering 2024;12(1):26. Doi: 10.3390/bioengineering12010026.
34. Hou W, Ji Z. Assessing GPT-4 for cell type annotation in single-cell RNA-seq analysis. Nature methods 2024;21(8):1462–1465. Doi: 10.1038/s41592-024-02235-4.
35. Feng Y, Wang J, He R, Zhou L, Li Y. A retrieval-augmented knowledge mining method with deep thinking LLMs for biomedical research and clinical support. GigaScience 2025;14:giaf109. Doi: 10.1093/gigascience/giaf109.
36. Franzén O, Gan LM, Björkegren JLM. PanglaoDB: a web server for exploration of mouse and human single-cell RNA sequencing data. Database 2019;2019:baz046. doi:https://note.org/10.1093/database/baz046, 10.1093/database/baz046.
37. Sauro HM, Agmon E, Blinov ML, Gennari JH, Hellerstein J, Heydarabadipour A, et al. From FAIR to CURE: Guidelines for Computational Models of Biological Systems. arXiv 2025;https://arxiv.org/abs/2502.15597, preprint. arXiv:2502.15597.
38. PBMCs from a healthy donor (v3, 3k cells, 150×150);. https://cf.10xgenomics.com/samples/cell/pbmc3k/pbmc3k_filtered_gene_bc_matrices.tar.gz. Accessed: 2025-11-14. 10x Genomics.

Click here to access/download;LaTeX - Figure (eps, ps, etc.);example-grid-100x100pt.png

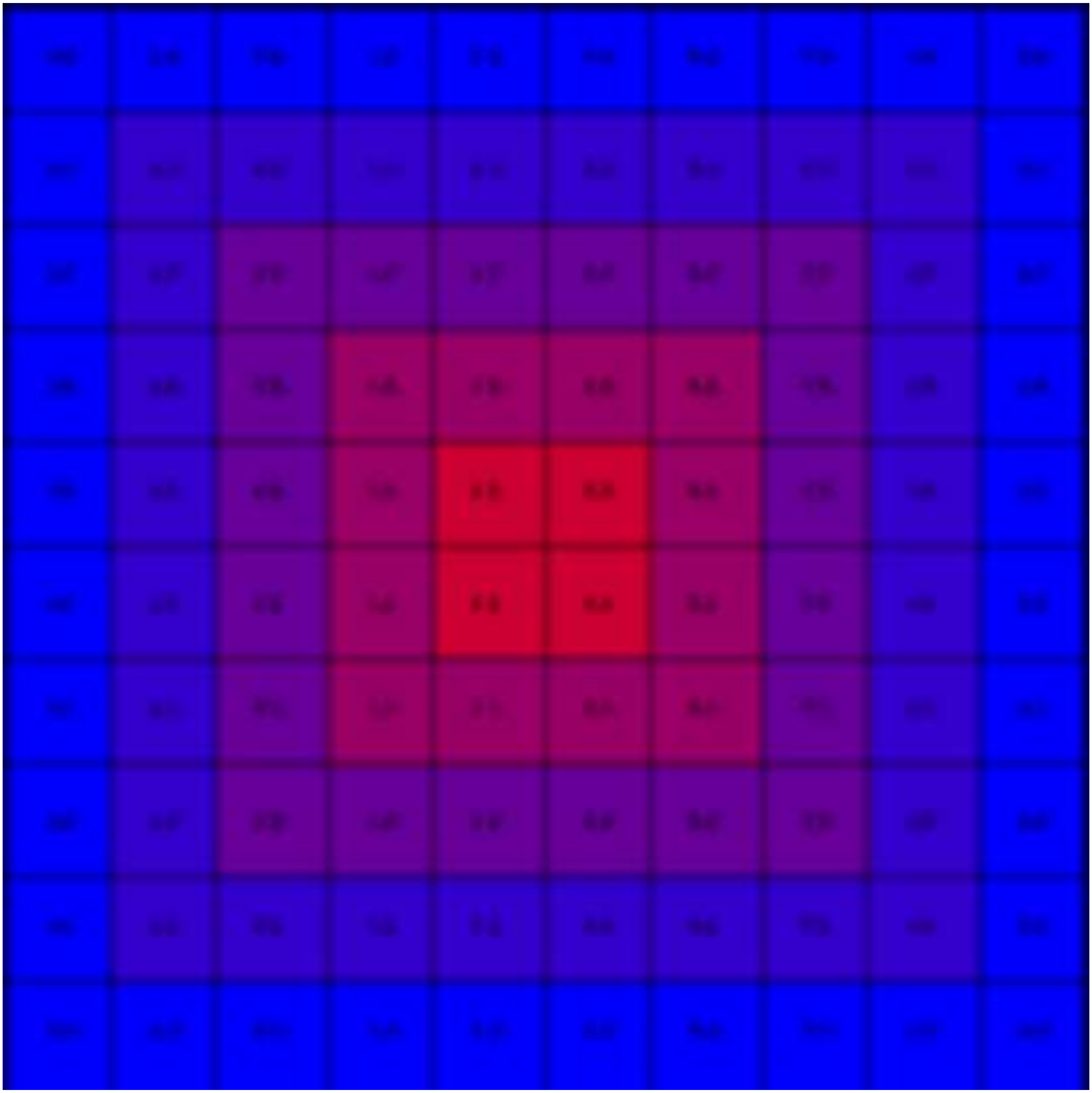

Click here to access/download;LaTeX - Figure (eps, ps, etc.);example-image.png

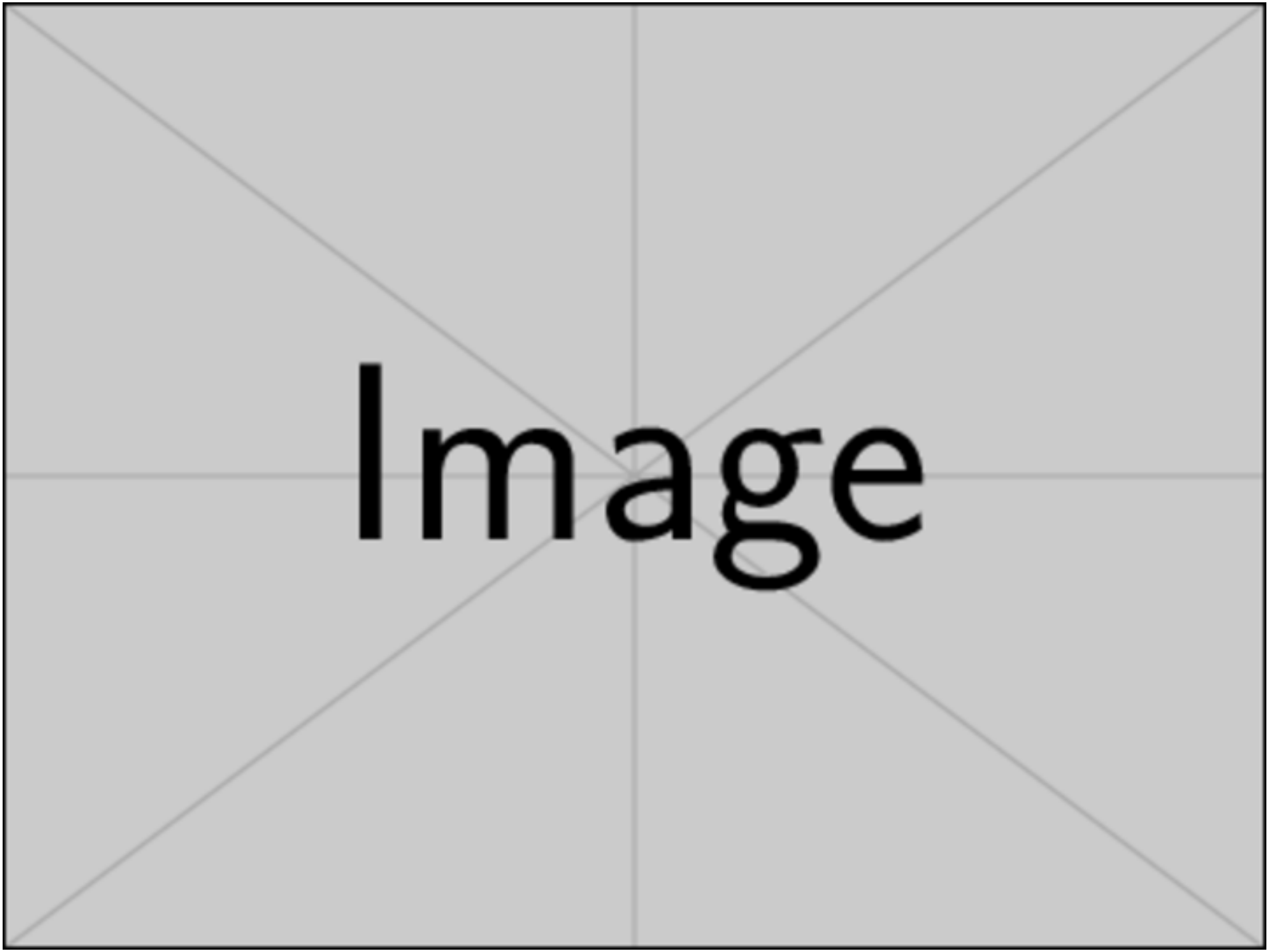

Manuscript Click here to access/download;Manuscript;Manuscript.pdf

Your PDF file "Manuscript.pdf" cannot be opened and processed. Please see the common list of problems, and suggested resolutions below.

Reason:

Other Common Problems When Creating a PDF from a PDF file

You will need to convert your PDF file to another format or fix the current PDF file, then re-submit it.

Cover Letter Click here to access/download;Personal Cover;Cover Letter.docx

Dear Editors,

On behalf of all co-authors, I am pleased to submit our manuscript entitled "SCSEQ: A web tool for analyzing single-cell RNA-seq data" for consideration in GigaScience.

Single-cell RNA-sequencing outputs are growing faster than most wet-lab teams can analyse. Existing platforms either demand expert coding or stop short of advanced tasks. We therefore built SCSEQ, a knowledge-driven, cloud-native pipeline that wraps benchmarked tools into an intuitive Vue/Flask framework. A retrieval-augmented LLM module draws on curated single-cell corpora to suggest cell-type labels, while a MySQL-backed task manager logs every parameter set and result for full reproducibility. Users can launch downstream analyses from the same dashboard, adjust visual aesthetics in real time, and compare outputs across parameter sets without re-uploading data. All plots are downloadable.

The highlights of this manuscript are:

• SCSEQ provides a no-code pipeline for single-cell transcriptome data analysis from raw data to publication-quality visualizations.
• A highly integrated system that enables flexible fine-tuning and real-time interactive visualization guarantees reliable downstream data analysis.
• Supporting cell type annotation with models trained on user datasets.
• Improving cell type annotation with RAG-enhanced large language models.

Our work is a strong fit for GigaScience. SCSEQ integrates a large amount of single-cell transcriptome sequencing data into a unified pipeline, promoting accessibility and reproducibility. Its use artificial Intelligence to enhance annotation accuracy exemplifies the computational workflows GigaScience values. SCSEQ's focus on usability and utility aligns with the journal's criteria for publication.

We would like to recommend the following four reviewers who have profound attainments in this research field and are directly related to our work for your reference:

1. Song Feng (song.feng@pnnl.gov)
Pacific Northwest National Laboratory (DOE)

2. Shuao Wang (shuaowang@suda.edu.cn)
School for Radiological & Interdisciplinary sciences, Soochow University

3. Per Eklund (perek@ifm.liu.sse)
Department of Physics, Linkoping University

4. Jinlong Zhu (jlzhu04@physics.unlv.edu)

High Pressure Science and Engineering Center and Department of Physics and Astronomy, University of Nevada

Neither the manuscript nor the underlying study violates any ethical guidelines; all data are from public repositories. The authors declare no competing interests.

Thank you for considering our work for publication.

Sincerely,

Shiyu Du, Ph.D. (Email:dushiyu@nimte.ac.cn)
School of Materials Science and Engineering,
China University of Petroleum (East China), Qingdao,
Shandong 266580, P.R. China.
School of Computer Science and Technology,
China University of Petroleum (East China), Qingdao,
Shandong 266580, P.R. China.

Jian He, Ph.D. (Email: jih003@sjtu.edu.cn)
Director of Genomics Core, Center for Single-Cell Omics
Associate Professor, State Key Laboratory of Systems Medicine for Cancer
Shanghai Jiao Tong University School of Medicine
Shanghai, PR China
