# Supplementary material for: SCSEQ: A web tool for analyzing single-cell RNA-seq data
Source: Gigascience. 2026 May 5;15:giag029. doi: 10.1093/gigascience/giag029 (PMC13142160; doi:10.1093/gigascience/giag029)
Supplement: giag029_Authors_Response_To_Reviewer_Comments_original_submission [file giag029_authors_response_to_reviewer_comments_original_submission.pdf]

Author's Response To Reviewer Comments

Responds to the reviewer’s comments:

Reviewer #1:

1. Response to comment: Lack of documentation and tutorial resources
Since the platform is intended for non-specialists, accessible documentation is essential. The manuscript does not show how data should be prepared, how tasks are executed, how parameters affect outputs, or how results are interpreted. Please provide a step-by-step tutorial or link to documentation, this would make the platform substantially more usable.
Response:
We thank the reviewer for highlighting the importance of documentation for non-specialist users. We agree that clear and accessible guidance is essential for a web-based analysis platform. In the revised manuscript, we have added a dedicated tutorial that provides a step-by-step walkthrough of a typical analysis workflow, covering data preparation, task execution, parameter configuration, and basic interpretation of results. A permanent link to this documentation has been added to the Methods section describing the platform implementation. To further improve usability, each major operation step in the platform interface is accompanied by a contextual help button, which directs users to the corresponding section of the tutorial. These revisions are intended to make the platform easier to follow for first-time users without requiring prior computational expertise.

2. Response to comment: Lack of Single-Cell Transcription Factor Analysis Module
Transcription factors participate in regulating genomic DNA accessibility, recruiting RNA polymerase for transcription, and assembling cofactors to modulate specific transcription stages during biological processes. They also govern immune responses and developmental processes in organisms. Therefore, integrating a single-cell transcription factor analysis module into cloud platforms is essential—such as one based on SCENIC.
SCENIC: single-cell regulatory network inference and clustering. https://doi.org/10.1038/nmeth.4463
Response:
We thank the reviewer for emphasizing the importance of incorporating transcription factor analysis into single-cell analysis platforms. We fully agree that transcription factors play a central role in regulating chromatin accessibility, coordinating transcriptional programs, and shaping immune and developmental processes. In response to this suggestion, we have integrated a dedicated transcription factor analysis module into SCSEQ based on the SCENIC framework. This module enables the inference of gene regulatory networks and the assessment of transcription factor activity at single-cell resolution. In the revised manuscript, we have added a description of this functionality in both the Downstream Analyses section and the Data Analysis subsection to clarify its implementation and practical usage within the workflow. We have also included the corresponding reference to SCENIC to appropriately acknowledge the methodological foundation. We believe that this addition further strengthens its downstream analytical capabilities.

3. Response to comment: Validation limited to PBMC data
The evaluation is based solely on a 10x PBMC dataset, which is a minimal and well-behaved example. It does not demonstrate performance under realistic biological scenarios (tumors, multi-sample studies, or disease contexts). I suggest adding at least one dataset involving tumor heterogeneity, multi-sample integration, or disease states to show that the platform can handle more complex settings.
Response :
We thank the reviewer for this valuable suggestion. We agree that validation using only a PBMC dataset is insufficient to demonstrate the applicability of the platform in real-world research scenarios.
In the revised manuscript, we have added an additional analysis based on a real-world liver cancer single-cell dataset containing approximately 80,000 cells. This dataset originates from another study conducted by our research group and represents a biologically complex tumor microenvironment with substantial cellular heterogeneity. The analysis was performed strictly following the standard SCSEQ workflow, and key downstream results are presented to illustrate the platform’s performance in a realistic disease context.

For cell annotation, we applied the CellTypist reference model “Healthy_Human_Liver”, which enabled the identification of major liver and immune cell populations. Marker genes for each annotated cell type were calculated and visualized using dot plots, allowing intuitive inspection of cell-type-specific expression patterns. (Related images can be found in the file "Response to Reviewer")

Figure 1. Cell annotation results with the CellTypist reference model “Healthy_Human_Liver”

Figure 2. Visualization of marker gene expression

To demonstrate downstream functional analysis capability, we selected B cells and performed Gene Enrichment Analysis, presenting the top enriched biological processes.

Figure 3. Gene enrichment analysis of B cells showing the top enriched biological processes.

We further conducted cell–cell communication analysis using CellChat, and visualized the interaction network as circular plots, illustrating the communication landscape among major cell populations.

Figure 4. Cell–cell communication network inferred by CellChat and visualized as circular interaction plots among major cell populations.

To assess genomic instability in tumor-related populations, Copy Number Variation (CNV) analysis was performed using InferCNV. Endothelial_cells were used as the reference population, and CNV patterns were evaluated in Hepatocytes and Macrophages, revealing distinct large-scale chromosomal alterations consistent with tumor-associated changes.

Figure 5. Copy number variation analysis using InferCNV with Endothelial_cells as the reference and CNV patterns evaluated in Hepatocytes and Macrophages.

We also performed pseudotime analysis to investigate potential cellular state transitions within the tumor microenvironment. Three representative cell populations (T_cells, B_cells, and Endothelial_cells) were selected, and their developmental trajectories were reconstructed to illustrate dynamic changes along inferred biological progression.

Figure 6. Pseudotime trajectory analysis of T_cells, B_cells, and Endothelial_cells illustrating dynamic cellular state transitions.

In addition, to demonstrate the platform’s clinical analysis capability, we conducted pan-cancer analysis using the gene KRT18. Results include differential expression between tumor and normal tissues across cancer types, correlations with CpG methylation and immune scores, and survival associations (OS, DSS, DFI, and PFI), highlighting the clinical relevance of the analysis.
Together, these results demonstrate that SCSEQ can effectively handle large-scale datasets, tumor heterogeneity, and complex multi-level analyses, supporting its practical utility in disease-oriented single-cell studies.

Figure 7. Pan-cancer analysis of KRT18 across TCGA cohorts showing expression differences, methylation and immune correlations, and survival associations.

4. Response to comment: Data security and multi-user isolation
As SCSEQ is cloud-based, users will reasonably ask how uploaded data are stored, isolated, and protected. The manuscript does not describe project isolation, storage policies or what metadata persist after task deletion. Clarification of these aspects would improve confidence in the platform and support broader adoption.
Response:
We thank the reviewer for highlighting this important point. In the revised manuscript, we have expanded the description of the system architecture to clarify data security and multi-user isolation mechanisms.

Specifically, in the Implementations section describing the system architecture, we now state that each user project is assigned an independent workspace, with strict separation enforced at both the file system and database levels through user and project identifiers. Uploaded data are accessible only within the corresponding project scope, and intermediate files are not shared across projects.
In addition, description of the Data Layer has been updated to explain how user_id and project_id are used jointly to constrain database queries and enforce separation between users and projects. We have also clarified the data storage and deletion policy in the file system management description, specifying that deleting a task removes its associated result files, while deleting a project triggers removal of all files under the corresponding project directory. Together, these revisions clarify how uploaded data are stored, isolated, and removed, and are intended to improve user confidence in the platform.

5. Response to comment: Provide the link or repository for the RAG reference dataset.
Response:
We have added explicit descriptions and corresponding links for the reference datasets used in the RAG module in the revised manuscript (Methods, Cell type identification section), and these resources are now cited to improve transparency and reproducibility.

6. Response to comment: Fix inconsistencies between the citation numbering and the order in which they appear in the text.
Response:
We have reviewed and revised the manuscript to ensure that all references are cited in the correct numerical order.

7. Response to comment: Figure 4 should clearly specify which gene was used to generate the futureplot and violin plot.
Response:
The gene used to generate the FeaturePlot and violin plot in Figure 4 has now been explicitly specified. In the revised manuscript, we state that CD79A was selected, and we clarify in the main text that the same gene was used for both visualizations to ensure consistency.

8. Response to comment: State which LLM model was used and basic deployment configuration.
Response:
The large language model used in the annotation module is now explicitly specified in the manuscript. We used Qwen-Max (Alibaba Tongyi Qianwen series) as the underlying LLM. The model is accessed through the official cloud 1and is used to generate candidate cell-type labels and supporting explanations based on the marker genes and tissue information provided after the RAG retrieval step. Basic implementation details have been added to improve transparency and reproducibility (Methods, Cell type identification).
To minimize data exposure, only the top marker genes and tissue information are sent to the external LLM service during the annotation step. No raw expression matrices, cell-level data, or user-uploaded datasets are transmitted. All primary data processing and analysis are performed locally within the SCSEQ environment.

9. Response to comment: Expand "RAG" on first use as "Retrieval-Augmented Generation".
Response:
The term “Retrieval-Augmented Generation (RAG)” is now defined at its first occurrence in the Introduction.

10. Response to comment: Do not capitalize "User" unless it is defined as a proper entity.
Response:
The inappropriate capitalization of “User” has been corrected at two locations, specifically in the Key Points section and in the Results and Discussion – Data Analysis subsection.

11. Response to comment: Correct the grammar in "posing significant challenge".
Response:
The phrase “posing significant challenge” has been corrected to “posing a significant challenge” in the Abstract section.

Reviewer #2:
1. Response to comment: The most pressing issue is the availability of the web tool. The manuscript lists the platform URL as https://ape-closing-tightly.ngrok-free.app. This is an ngrok tunnel, typically used for local development and debugging. It is not a persistent, production-grade domain. Such a link relies on the author's local machine being turned on and the tunnel being active. For a web tool publication, a stable, persistent server (institutional or cloud-hosted) with a proper domain is a strict requirement. If the authors intend for users to install this locally, the "web tool" framing is slightly misleading, and the focus should shift to the Docker/local deployment ease. However, the manuscript frames it heavily as a "web-based bioinformatics analysis platform".
Response:
We thank the reviewer for raising this important concern regarding the stability and persistence of the web platform. We fully agree that a temporary ngrok tunnel is not suitable for a production-level web tool publication. In response, we have migrated the system from the temporary development tunnel to a permanently deployed cloud server with a dedicated domain.
The platform is now publicly accessible via a stable HTTPS domain: https://scseq.com.cn. The service is hosted on a persistent cloud server and configured with a valid SSL certificate, ensuring secure encrypted communication (HTTPS) and long-term availability. The domain is officially registered and maintained. The manuscript has been updated accordingly to reflect the new permanent URL.

2. Response to comment: Figure 3 (Task Information Dialog) clearly displays User Interface (UI) elements in Chinese (e.g., "任务信息" for Task Information, "运行状态" for Status, "提交时间" for Submission Time, "操作" for Operation). As this is a submission to an international English-language journal, the software interface shown in the manuscript must be in English. If the tool supports multi-language localization, this should be explicitly stated, but the default figures should be in English to ensure readability for the reviewers and readership.
Response:
We thank the reviewer for pointing out this issue. In the revised manuscript, Figure 3 has been replaced with an English-language interface to ensure readability for an international audience. In addition, a language selection button has been added to the top-right corner of the platform interface, allowing users to switch between Chinese and English, with English set as the default language. This functionality has also been clarified in the manuscript to indicate that SCSEQ supports bilingual localization while maintaining English as the primary interface.

3. Response to comment: The authors claim a "Pan-cancer analysis" feature that distinguishes SCSEQ from competitors. However, the description suggests this is merely a lookup tool for The Cancer Genome Atlas (TCGA) bulk data, rather than an analysis of the user's uploaded single-cell data. Figure 5E shows boxplots of CD8A expression in TCGA samples. Does the tool actually project the user's single-cell clusters onto TCGA bulk data (e.g., deconvolution or similarity scoring)? If it simply allows a user to "search" a gene name and see static TCGA plots, it is misleading to list this as a downstream analysis of the scRNA-seq pipeline. This needs clarification.
Response:
We thank the reviewer for raising this important point and agree that the scope of the “Pan-cancer analysis” module requires clearer explanation.
SCSEQ does not project user-uploaded single-cell clusters directly onto The Cancer Genome Atlas (TCGA) bulk RNA-seq data through deconvolution or similarity scoring. Instead, the Pan-cancer module is designed as an integrated translational analysis component that connects genes identified from the user’s single-cell analysis to large-scale TCGA bulk cohorts for downstream validation and clinical relevance assessment.
Specifically, once a user identifies genes of interest from scRNA-seq analyses, SCSEQ enables automated pan-cancer evaluation of those genes across TCGA datasets. This includes: (1) expression comparison between tumor and normal tissues, (2) correlation analysis between gene expression and CpG methylation levels, (3) correlation analysis with immune infiltration scores, and (4) univariate Cox regression across cancer types with forest plot visualization. These analyses are dynamically generated based on the queried gene and are not static images.
In response to the reviewer’s comment, we have further expanded this module by incorporating Kaplan–

Meier survival analysis in selected cancer types (e.g., ACC for CD8A). Patients are stratified into high- and low-expression groups based on the median expression level, and performs log-rank testing, providing statistical evidence of prognostic relevance. This addition strengthens the clinical relevance of the Pan-cancer module.
Therefore, the Pan-cancer module should be interpreted as a gene-level translational extension of the scRNA-seq workflow rather than as a projection of single-cell clusters onto bulk data. Its purpose is to facilitate rapid clinical validation and cross-cancer comparison of candidate genes discovered in single-cell analyses.
To avoid misunderstanding, we have revised the manuscript to (i) clarify that SCSEQ does not perform bulk–single-cell deconvolution or similarity projection, and (ii) explicitly describe all analytical steps included in the TCGA-based module, including methylation, immune correlation, Cox regression, and Kaplan–Meier survival analysis. We believe these clarifications more accurately define the functionality and scope of the Pan-cancer analysis feature.

4. Response to comment: The manuscript mentions using a "RAG-enhanced large language model" for cell annotation, utilizing PanglaoDB. However, critical technical details are missing: Which LLM? (e.g., GPT-4, Llama 3, Claude, or a custom model?). How is "semantic similarity computed in an embedding space"? Which embedding model is used? The authors explicitly state, "accuracy... cannot be guaranteed" and "results should be regarded as advisory". While honesty is good, including an experimental feature with no validation metrics (e.g., F1 score comparison against CellTypist) weakens the paper.
Response:
We thank the reviewer for this insightful comment. In the revised manuscript, we have provided additional technical details to clarify the implementation of the RAG-enhanced annotation module and its intended role.
Specifically, the large language model used in this study is Qwen-Max, accessed via an API service. For semantic retrieval, the external knowledge corpus is first segmented into text chunks and encoded using a SentenceTransformer model (text2vec-base-chinese). The resulting embeddings are stored and indexed in ChromaDB, where similarity between query and corpus entries is computed based on vector similarity in the embedding space. For each query, the top candidate records are retrieved and further re-ranked using a CrossEncoder model (mmarco-mMiniLMv2-L12-H384) to improve relevance. The highest-ranked records are then provided to the LLM as contextual references for generation.
We also clarify the intended role of this module. The RAG-based LLM component is designed as an exploratory and advisory tool to assist users in interpreting cluster identities, rather than as a primary or automated annotation method. The main analytical results in SCSEQ still rely on conventional approaches such as clustering-based marker identification and user validation. In our workflow, cells are first grouped into clusters, and the top marker genes (top 10) for each cluster, together with the tissue context, are provided to the LLM to infer a representative cell-type label at the cluster level. This strategy is conceptually similar to recently reported tools such as GPTCelltype and AICelltype, and is intended to provide interpretable suggestions based on biologically meaningful marker patterns.
Because the annotation is performed at the cluster level rather than the individual cell level, each predicted label affects a large group of cells simultaneously. As a result, performance metrics calculated at the cell level (e.g., F1 score) can show large fluctuations and may not accurately reflect the practical usefulness of the method. Moreover, the outcome is highly dependent on clustering quality, cell-type composition, and dataset characteristics. Given the auxiliary nature of this functionality and the variability across biological datasets, we do not position LLM outputs as definitive annotations, and therefore standalone benchmarking metrics against dedicated annotation tools are not included. This intended usage and limitation have now been explicitly stated in the manuscript.

5. Response to additional concern: The backend uses Flask and stores "Raw Files" which are "storage-heavy". The manuscript does not address how the server handles multiple concurrent users uploading large scRNA-seq matrices (which can be gigabytes in size). The demo uses a very small PBMC dataset (2,700 cells). To prove the tool is robust, the authors should benchmark it against a larger dataset (e.g., 50k+ cells) to demonstrate that the web server (and the R backend) does not time out or crash during clustering/integration.
Response:
We agree that demonstrating SCSEQ’s scalability requires more than small PBMC datasets. In response, we

performed additional testing on a larger, internal dataset containing over 80,000 cells. While the computation time is longer, the platform completes the workflow stably without timeouts or task failures, demonstrating robust performance under heavier data loads.
Furthermore, we have clarified the system's design for handling multiple concurrent users and tasks. SCSEQ supports asynchronous background execution for all analysis jobs, allowing multiple tasks from different users or projects to run independently. Each project has an isolated workspace with strict data separation at both user and project levels. Project-level task scheduling ensures that analyses are executed independently, preventing interference between concurrent tasks. These improvements are described in the revised Implementation section, highlighting multi-task handling, project isolation, and concurrency control.

6. Response to additional concern: In Table 1, the checkmarks for "Pan-cancer analysis" and "Copy number variation" favor SCSEQ. However, without clarification on how these are implemented (especially Pan-cancer), this comparison may be unfair to other established platforms like ezSingleCell which may have different scopes.
Response:
We appreciate the reviewer's comment regarding Table 1. To make the comparison fairer, we have clarified how SCSEQ implements features such as Pan-cancer analysis and Copy Number Variation analysis, including the TCGA-related processing steps described in our previous third response. At the same time, we acknowledge that SCSEQ currently does not include modules for spatial transcriptomics or other multi-omics analyses; our platform is focused specifically on single-cell transcriptomic data analysis. These clarifications have been added to the manuscript to ensure that readers understand the scope of SCSEQ and can interpret the comparison with other platforms, such as ezSingleCell, in the proper context.

7. Response to additional concern: Figure 4D (Marker gene identification) is quite small and dense; the dotplot text is barely legible.
Response:
We appreciate the reviewer's comment. The marker gene dotplot in Figure 4D has been resized and its layout adjusted to improve readability. Font spacing have been optimized so that both gene names and values are clearly legible, ensuring that the key information is easily interpretable.

8. Response to additional concern: "Looking forward,," (double comma).
Response:
All identified typographical issues, including the duplicated panel label in Figure 5 and the double comma in "Looking forward, ,", have been corrected.

Responds to the editor's comments:
Thank you for your letter and for the reviewers' comments concerning our manuscript. Those comments are all valuable for revising and improving our paper and the important guiding significance to our research. We have studied comments and advice carefully and have made corrections.
Following your suggestion, we have carefully reviewed and incorporated the five cited articles into the Related Work and Discussion sections of the revised manuscript. Meanwhile, in the 'marked manuscript', the revision are highlighted in yellow. We believe that the inclusion of these studies strengthens the scholarly context of our work and better aligns the manuscript with current methodological developments in the field. We appreciate the guidance, which has helped improve the overall quality and positioning of the manuscript.

Cookie Preference Center

We use cookies which are necessary to make our site work. We may also use additional cookies to analyze, improve and personalize our content and your digital experience. For more information, see our Cookie Policy.

You may choose not to allow some types of cookies. However, blocking some types may impact your experience of our site and the services we are able to offer. See the different category headings below to find out more or change your settings.

Allow all

Manage Consent Preferences

Strictly Necessary Cookies

Always active

These cookies are necessary for the website to function and cannot be switched off in our systems. They are usually only set in response to actions made by you which amount to a request for services, such as setting your privacy preferences, logging in or filling in forms. You can set your browser to block or alert you about these cookies, but some parts of the site will not then work.

Functional Cookies

These cookies enable the website to provide enhanced functionality and personalisation. They may be set by us or by third party providers whose services we have added to our pages. If you do not allow these cookies then some or all of these services may not function properly.

Performance Cookies

These cookies allow us to count visits and traffic sources so we can measure and improve the performance of our site. They help us to know which pages are the most and least popular and see how visitors move around the site.

Targeting Cookies

These cookies may be set through our site by our advertising partners. They may be used by those companies to build a profile of your interests and show you relevant adverts on other sites. If you do not allow these cookies, you will experience less targeted advertising.

Cookie List

Clear

Apply Cancel

Consent Leg.Interest

Confirm my choices

Power
