# Supplementary material for: SCSEQ: A web tool for analyzing single-cell RNA-seq data
Source: Gigascience. 2026 May 5;15:giag029. doi: 10.1093/gigascience/giag029 (PMC13142160; doi:10.1093/gigascience/giag029)
Supplement: giag029_Reviewer_1_Report_original_submission — Reviewer 1 -- 1/18/2026 [file giag029_reviewer_1_report_original_submission.pdf]

Reviewer Report

Title: SCSEQ: A web tool for analyzing single-cell RNA-seq data

Version: Original Submission **Date:** 1/18/2026

Reviewer name: Reviewer 1

Reviewer Comments to Author:

The manuscript presents SCSEQ, a web-based platform that integrates multiple scRNA-seq analysis modules (preprocessing, annotation, CNV, trajectory inference, and pan-cancer analysis). The tool is positioned for users without programming experience and provides graphical workflows and interactive outputs. The concept is useful and potentially valuable to the community, but several issues should be addressed before publication.

Major Comments:

1. Lack of documentation and tutorial resources

Since the platform is intended for non-specialists, accessible documentation is essential. The manuscript does not show how data should be prepared, how tasks are executed, how parameters affect outputs, or how results are interpreted. Please provide a step-by-step tutorial or link to documentation, this would make the platform substantially more usable.

2. Lack of Single-Cell Transcription Factor Analysis Module

Transcription factors participate in regulating genomic DNA accessibility, recruiting RNA polymerase for transcription, and assembling cofactors to modulate specific transcription stages during biological processes. They also govern immune responses and developmental processes in organisms. Therefore, integrating a single-cell transcription factor analysis module into cloud platforms is essentialâ€”such as one based on SCENIC.

SCENIC: single-cell regulatory network inference and clustering. https://doi.org/10.1038/nmeth.4463

3. Validation limited to PBMC data

The evaluation is based solely on a 10x PBMC dataset, which is a minimal and well-behaved example. It does not demonstrate performance under realistic biological scenarios (tumors, multi-sample studies, or disease contexts). I suggest adding at least one dataset involving tumor heterogeneity, multi-sample integration, or disease states to show that the platform can handle more complex settings.

4. Data security and multi-user isolation

As SCSEQ is cloud-based, users will reasonably ask how uploaded data are stored, isolated, and protected. The manuscript does not describe project isolation, storage policies or what metadata persist after task deletion. Clarification of these aspects would improve confidence in the platform and support broader adoption.

Minor Comments:

1. Provide the link or repository for the RAG reference dataset.
2. Fix inconsistencies between the citation numbering and the order in which they appear in the text.
3. Figure 4 should clearly specify which gene was used to generate the futureplot and violin plot.
4. State which LLM model was used and basic deployment configuration.

5. Expand "RAG" on first use as "Retrieval-Augmented Generation".
6. Do not capitalize "User" unless it is defined as a proper entity.
7. Correct the grammar in "posing significant challenge".

Methods

Are the methods appropriate to the aims of the study, are they well described, and are necessary controls included? Choose an item.

Conclusions

Are the conclusions adequately supported by the data shown? Choose an item.

Reporting Standards

Does the manuscript adhere to the journal's guidelines on [minimum standards of reporting?] Choose an item.

Choose an item.

Statistics

Are you able to assess all statistics in the manuscript, including the appropriateness of statistical tests used? Choose an item.

Quality of Written English

Please indicate the quality of language in the manuscript: Choose an item.

Declaration of Competing Interests

Please complete a declaration of competing interests, considering the following questions:

- Have you in the past five years received reimbursements, fees, funding, or salary from an organisation that may in any way gain or lose financially from the publication of this manuscript, either now or in the future?
- Do you hold any stocks or shares in an organisation that may in any way gain or lose financially from the publication of this manuscript, either now or in the future?
- Do you hold or are you currently applying for any patents relating to the content of the manuscript?
- Have you received reimbursements, fees, funding, or salary from an organization that holds or has applied for patents relating to the content of the manuscript?
- Do you have any other financial competing interests?
- Do you have any non-financial competing interests in relation to this paper?

If you can answer no to all of the above, write 'I declare that I have no competing interests' below. If your reply is yes to any, please give details below.

I declare that I have no competing interests

I agree to the open peer review policy of the journal. I understand that my name will be included on my report to the authors and, if the manuscript is accepted for publication, my named report including any attachments I upload will be posted on the website along with the authors' responses. I agree for my report to be made available under an Open Access Creative Commons CC-BY license (http://creativecommons.org/licenses/by/4.0/). I understand that any comments which I do not wish to be included in my named report can be included as confidential comments to the editors, which will not be published.

Choose an item.

To further support our reviewers, we have joined with Publons, where you can gain additional credit to further highlight your hard work (see: https://publons.com/journal/530/gigascience). On publication of this paper, your review will be automatically added to Publons, you can then choose whether or not to claim your Publons credit. I understand this statement.

Yes Choose an item.
