# Supplementary material for: SCSEQ: A web tool for analyzing single-cell RNA-seq data
Source: Gigascience. 2026 May 5;15:giag029. doi: 10.1093/gigascience/giag029 (PMC13142160; doi:10.1093/gigascience/giag029)
Supplement: giag029_Reviewer_2_Report_original_submission — Reviewer 2 -- 1/21/2026 [file giag029_reviewer_2_report_original_submission.pdf]

Reviewer Report

Title: SCSEQ: A web tool for analyzing single-cell RNA-seq data

Version: Original Submission **Date:** 1/21/2026

Reviewer name: Reviewer 2

Reviewer Comments to Author:

The manuscript introduces SCSEQ, a web-based bioinformatics platform designed to facilitate single-cell RNA sequencing (scRNA-seq) analysis for researchers without programming expertise. The platform integrates established tools (Seurat, Monocle, InferCNV, CellTypist) into a graphical user interface (GUI) built with Vue.js and Flask. The authors claim SCSEQ supports a comprehensive workflow ranging from quality control to advanced downstream tasks like trajectory inference, copy number variation (CNV) detection, and a unique "pan-cancer analysis" module.
While the goal of democratizing scRNA-seq analysis is commendable and fits the scope of GigaScience, the manuscript suffers from significant technical and presentation flawsâ€”most notably the use of a temporary, unstable web address for the server and non-English localization in the interface figures. These issues must be addressed to meet the standards of a software publication.
The most pressing issue is the availability of the web tool. The manuscript lists the platform URL as https://ape-closing-tightly.ngrok-free.app. This is an ngrok tunnel, typically used for local development and debugging. It is not a persistent, production-grade domain. Such a link relies on the author's local machine being turned on and the tunnel being active. For a web tool publication, a stable, persistent server (institutional or cloud-hosted) with a proper domain is a strict requirement. If the authors intend for users to install this locally, the "web tool" framing is slightly misleading, and the focus should shift to the Docker/local deployment ease. However, the manuscript frames it heavily as a "web-based bioinformatics analysis platform".
Figure 3 (Task Information Dialog) clearly displays User Interface (UI) elements in Chinese (e.g., "ä»»åŠ¡ä¿¡æ�¯" for Task Information, "è¿�è¡ŒçŠ¶æ€�" for Status, "æ��äº¤æ—¶é—´" for Submission Time, "æ“�ä½œ" for Operation). As this is a submission to an international English-language journal, the software interface shown in the manuscript must be in English. If the tool supports multi-language localization, this should be explicitly stated, but the default figures should be in English to ensure readability for the reviewers and readership.
The authors claim a "Pan-cancer analysis" feature that distinguishes SCSEQ from competitors. However, the description suggests this is merely a lookup tool for The Cancer Genome Atlas (TCGA) bulk data, rather than an analysis of the user's uploaded single-cell data. Figure 5E shows boxplots of CD8A expression in TCGA samples. Does the tool actually project the user's single-cell clusters onto TCGA bulk data (e.g., deconvolution or similarity scoring)? If it simply allows a user to "search" a gene name and see static TCGA plots, it is misleading to list this as a downstream analysis of the scRNA-seq pipeline. This needs clarification.
The manuscript mentions using a "RAG-enhanced large language model" for cell annotation, utilizing PanglaoDB. However, critical technical details are missing: Which LLM? (e.g., GPT-4, Llama 3, Claude, or

a custom model?). How is "semantic similarity computed in an embedding space"? Which embedding model is used? The authors explicitly state, "accuracy... cannot be guaranteed" and "results should be regarded as advisory". While honesty is good, including an experimental feature with no validation metrics (e.g., F1 score comparison against CellTypist) weakens the paper.

The backend uses Flask and stores "Raw Files" which are "storage-heavy". The manuscript does not address how the server handles multiple concurrent users uploading large scRNA-seq matrices (which can be gigabytes in size). The demo uses a very small PBMC dataset (2,700 cells). To prove the tool is robust, the authors should benchmark it against a larger dataset (e.g., 50k+ cells) to demonstrate that the web server (and the R backend) does not time out or crash during clustering/integration.

In Table 1, the checkmarks for "Pan-cancer analysis" and "Copy number variation" favor SCSEQ. However, without clarification on how these are implemented (especially Pan-cancer), this comparison may be unfair to other established platforms like ezSingleCell which may have different scopes.

Figure 4D (Marker gene identification) is quite small and dense; the dotplot text is barely legible.

"Looking forward,," (double comma).

In summary, SCSEQ shows promise as an integrator of standard scRNA-seq tools into a no-code environment. However, the manuscript describes a tool that appears to be in a "beta" or "local development" stage (indicated by the ngrok URL and non-localized UI) rather than a finished product ready for publication. The authors must deploy the tool to a stable server, translate the UI for the figures, and provide rigorous benchmarking and technical details for the AI and Pan-cancer modules before acceptance.

Methods

Are the methods appropriate to the aims of the study, are they well described, and are necessary controls included? Choose an item.

Conclusions

Are the conclusions adequately supported by the data shown? Choose an item.

Reporting Standards

Does the manuscript adhere to the journal's guidelines on [minimum standards of reporting?]() Choose an item.

Choose an item.

Statistics

Are you able to assess all statistics in the manuscript, including the appropriateness of statistical tests used? Choose an item.

Quality of Written English

Please indicate the quality of language in the manuscript: Choose an item.

Declaration of Competing Interests

Please complete a declaration of competing interests, considering the following questions:

- Have you in the past five years received reimbursements, fees, funding, or salary from an organisation that may in any way gain or lose financially from the publication of this manuscript, either now or in the future?
- Do you hold any stocks or shares in an organisation that may in any way gain or lose financially from the publication of this manuscript, either now or in the future?
- Do you hold or are you currently applying for any patents relating to the content of the manuscript?
- Have you received reimbursements, fees, funding, or salary from an organization that holds or has applied for patents relating to the content of the manuscript?
- Do you have any other financial competing interests?
- Do you have any non-financial competing interests in relation to this paper?

If you can answer no to all of the above, write 'I declare that I have no competing interests' below. If your reply is yes to any, please give details below.

I declare that I have no competing interests

I agree to the open peer review policy of the journal. I understand that my name will be included on my report to the authors and, if the manuscript is accepted for publication, my named report including any attachments I upload will be posted on the website along with the authors' responses. I agree for my report to be made available under an Open Access Creative Commons CC-BY license (http://creativecommons.org/licenses/by/4.0/). I understand that any comments which I do not wish to be included in my named report can be included as confidential comments to the editors, which will not be published.

Choose an item.

To further support our reviewers, we have joined with Publons, where you can gain additional credit to further highlight your hard work (see: https://publons.com/journal/530/gigascience). On publication of this paper, your review will be automatically added to Publons, you can then choose whether or not to claim your Publons credit. I understand this statement.

Yes Choose an item.
